# 4-Anilinoquinazoline-based benzenesulfonamides as nanomolar inhibitors of carbonic anhydrase isoforms I, II, IX, and XII: design, synthesis, *in-vitro*, and *in-silico* biological studies

**DOI:** 10.1080/14756366.2022.2055553

**Published:** 2022-03-30

**Authors:** Hossam Nada, Ahmed Elkamhawy, Magda H. Abdellattif, Andrea Angeli, Chang Hoon Lee, Claudiu T. Supuran, Kyeong Lee

**Affiliations:** a BK21 FOUR Team and Integrated Research Institute for Drug Development, College of Pharmacy, Dongguk University-Seoul, Goyang, Republic of Korea; b Department of Pharmaceutical Organic Chemistry, Faculty of Pharmacy, Mansoura University, Mansoura, Egypt; c Department of Chemistry, College of Science, Taif University, Taif, Saudi Arabia; d NEUROFARBA Department, Sezione di Scienze Farmaceutiche, University of Florence, Sesto Fiorentino, Florence, Italy

**Keywords:** Quinazoline-benzenesulfonamide hybrids, Suzuki coupling, Carbonic anhydrase inhibitors, Molecular docking

## Abstract

Human carbonic anhydrase inhibitors (hCAIs) are a key therapeutic class with a multitude of novel applications such as anticonvulsants, topically acting antiglaucoma, and anticancer drugs. Herein, a new series of 4-anilinoquinazoline-based benzenesulfonamides were designed, synthesised, and biologically assessed as potential hCAIs. The target compounds are based on the well-tolerated kinase scaffold (4-anilinoquinazoline). Compounds **3a** (89.4 nM), **4e** (91.2 nM), and **4f** (60.9 nM) exhibited 2.8, 2.7, and 4 folds higher potency against hCA I when compared to the standard (AAZ, **V**), respectively. A single digit nanomolar activity was elicited by compounds **3a** (8.7 nM), **4a** (2.4 nM), and **4e** (4.6 nM) with 1.4, 5, and 2.6 folds of potency compared to AAZ (12.1 nM) against isoform hCA II, respectively. Structure-activity relationship (SAR) and molecular docking studies validated our design approach that revealed highly potent hCAIs.

## 1. Introduction

**Figure 1.**
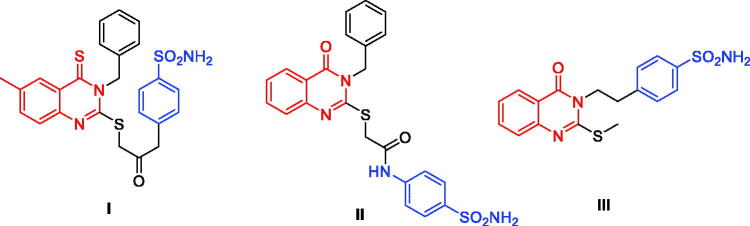
Chemical structures of potent quinazoline-based analogues as carbonic anhydrase inhibitors possessing primary sulphonamide (ZBG) moiety.

Carbonic anhydrases (CAs) are metalloenzymes responsible for converting CO_2_ to bicarbonate and proton in living organisms^1^. Human CAs of the α-class (α-CA) can be additionally grouped into sixteen isoforms that vary in cellular localisation, oligomeric structure, allocation in organs and tissues, kinetic properties, expression levels, and molecular characteristics^2^. Each isoform exhibits a specific subcellular localisation; CA I, II, III, VII, and XIII are cytosolic, CA IV, IX, XII, XIV, and XV are membrane-associated, while CA VA and VB are mitochondrial, and CA VI is secreted^3–5^. CAs are involved in a number of physiological activities such as breathing, pH regulation, bone resorption, ion transport, and gastric fluid secretion^6^. Consequently, CA inhibitors have become a leading therapeutic class in recent decades. Anticancer agents, anticonvulsants and topically acting antiglaucoma are some of the novel applications of CA inhibitors that have been reported^2^^,^^7^. Due to its expression in the endothelium of neovessels in melanoma, renal, lung, and oesophageal cancers, CA II is the most physiologically relevant isoform which was linked with various tumours including melanoma, oesophageal, renal, and lung cancers^8^. Inhibition of CA II also decreases angiogenesis *via* inhibition of vascular endothelial growth factor receptor (VEGFR) signaling^9^. The most common approach in designing CA inhibitors has been through the modulation of the ring directly bound to the zinc binding group (ZBG) or by attaching different “tails” to the aromatic ring as demonstrated by compounds **I**‒**III** (Figure 1)^10–13^.

**Figure 2.**
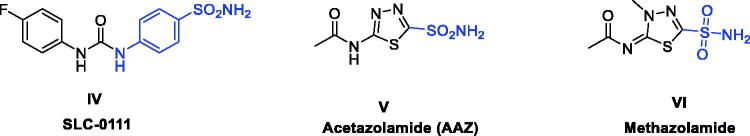
Chemical structure of some of the small molecule inhibitors currently in clinical trials.

Sulphonamides are a well-known ZBG moieties possessing effective carbonic anhydrase inhibitory activities, compounds **I**–**III** (Figure 1)^12^. Aromatic sulphonamides are reported to be strong and specific inhibitors of CA isoforms and deemed to be promising leads for further modifications and development of more potent inhibitors^13^. However, the lack of selectivity in blocking distinct isoforms is a key downside of CAIs, which is caused by structural similarity and identical subcellular location of these isoforms, resulting in adverse side effects^14^. Among the studied sulphonamides, the primary sulphonamide is one of the most effective ZBG for designing carbonic anhydrases inhibitors. This is owing to structural features that make them optimal for binding to the Zn^2+^ ion in the active site cavity and surrounding residues^15–17^. The negatively charged nitrogen of SO_2_NH^‒^ binds to the positively charged metal ion substituting the physiological zinc-bound nucleophile, where the proton found on the coordinated nitrogen atom acting as an acceptor via being at H-bond distance to Thr199 OG1 atom^16^. Several sulphonamide-based carbonic anhydrase inhibitors are currently in clinical trial targeting various diseases, such as SLC-0111 (**IV**, Figure 2) which is currently in phase I clinical trials as for patients with advanced tumours^18^. Acetazolamide (**V**, Figure 2) and Methazolamide (**VI**, Figure 2) are two examples of sulphonamide-based drugs which are currently undergoing phase IV clinical trials as carbonic anhydrase I inhibitors^19^^,^^20^.

**Figure 3.**
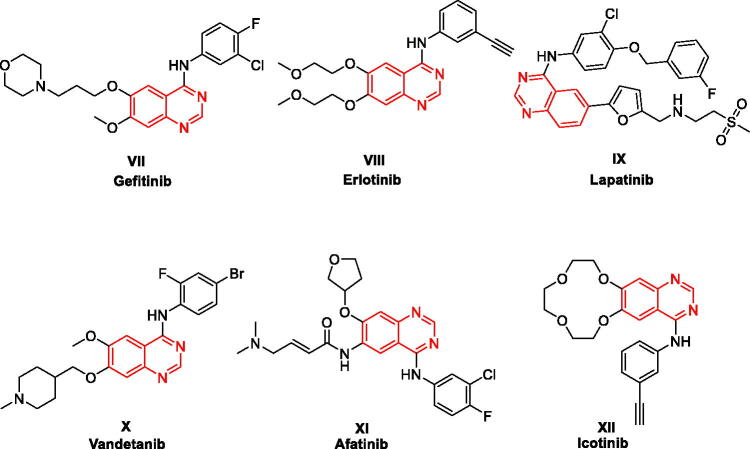
FDA approved 4-Anilinoquinazolines tyrosine kinase inhibitors.

4-Anilinoquinazolines have been frequently explored as anticancer agents due to their reported inhibitory activity on various receptor tyrosine kinases, such as VEGFR-2 (Vascular endothelial growth factor receptor 2), EGFR (epidermal growth factor receptor), or receptor tyrosine-protein kinase erbB-2 (HER2^21^^,^^22^. As illustrated in Figure 3, this heterocyclic class includes several approved drugs such as Gefitinib (Iressa^®^, **VII**), Erlotinib (Tarceva^®^, **VIII**), Lapatinib (Tykreb^®^, **IX**), Vandetanib (Caprelsa^®^, **X**), Afatinib (Gilotrif^®^, **XI**), and Icotinib (**XII**). Gefitinib (EGFR inhibitor) was approved by the FDA on May 2003 as a monotherapy to treat patients suffering from locally advanced or metastatic non-small cell lung cancer (NSCLC) following the failure of both platinum-based and docetaxel chemotherapies^23^. One year later, Erlotinib (EGFR inhibitor) was approved for treating NSCLC and in conjunction with gemcitabine for treatment of locally advanced, unrespectable, or metastatic pancreatic cancer^24^. In 2010, Lapatinib was approved for breast cancer treatment^25^. Lapatinib acts via inhibiting both HER2 and EGFR pathways and is utilised in combination therapies for HER2-positive breast cancer^26^. A year later, Vandetanib (a multi-kinase inhibitor) was approved as a treatment of metastatic medullary thyroid cancer^27^. Vandetanib exerts action through the inhibition of VEGFR, EGFR, and the RET-tyrosine kinase. Afatinib (EGFR/HER2 dual inhibitor) and Icotinib (EGFR inhibitor) were also approved for treatment of NSCLC^28^^,^^29^. These discoveries validate that 4-anilinoquinazoline is a privileged scaffold for developing anticancer drugs. Furthermore, the quinazoline ring was proved to be well-tolerated in the biological systems in terms of safety and potency^30^^,^^31^.

In the recent decade, some trials to develop quinazoline-based sulphonamides as CA inhibitors have been conducted. In 2014, a novel series of 2-substituted-mercapto-3-substituted-4(3*H*)-quinazolinones (**I**, Figure 1) was reported as α-carbonic anhydrases inhibitors from *Vibrio cholerae*^32^. Despite the high potency of these derivatives against *Vibrio cholerae*, they exhibited low inhibitory activity for the hCA I isoform (K_i_ range: 0.793–4.55 μM) and modest activity against the hCA II isoform (K_i_ range: 65.3 nM–0.114 μM). In the same year, another series of 4-oxoquinazoline containing a benzenesulfonamide moiety (**II**, Figure 1) was reported as a novel CA inhibitor against the protozoan enzyme of *Trypanosoma cruzi*^33^. The Oxoquinazoline (**II**) demonstrated moderate inhibition when tested against hCA I (K_i_ range: 86.5 nM– 5.43 μM) and a higher potency over hCA II isoforms. However, they have not been tested against the cancer-related hCA IX and XII isoforms.

**Figure 4.**
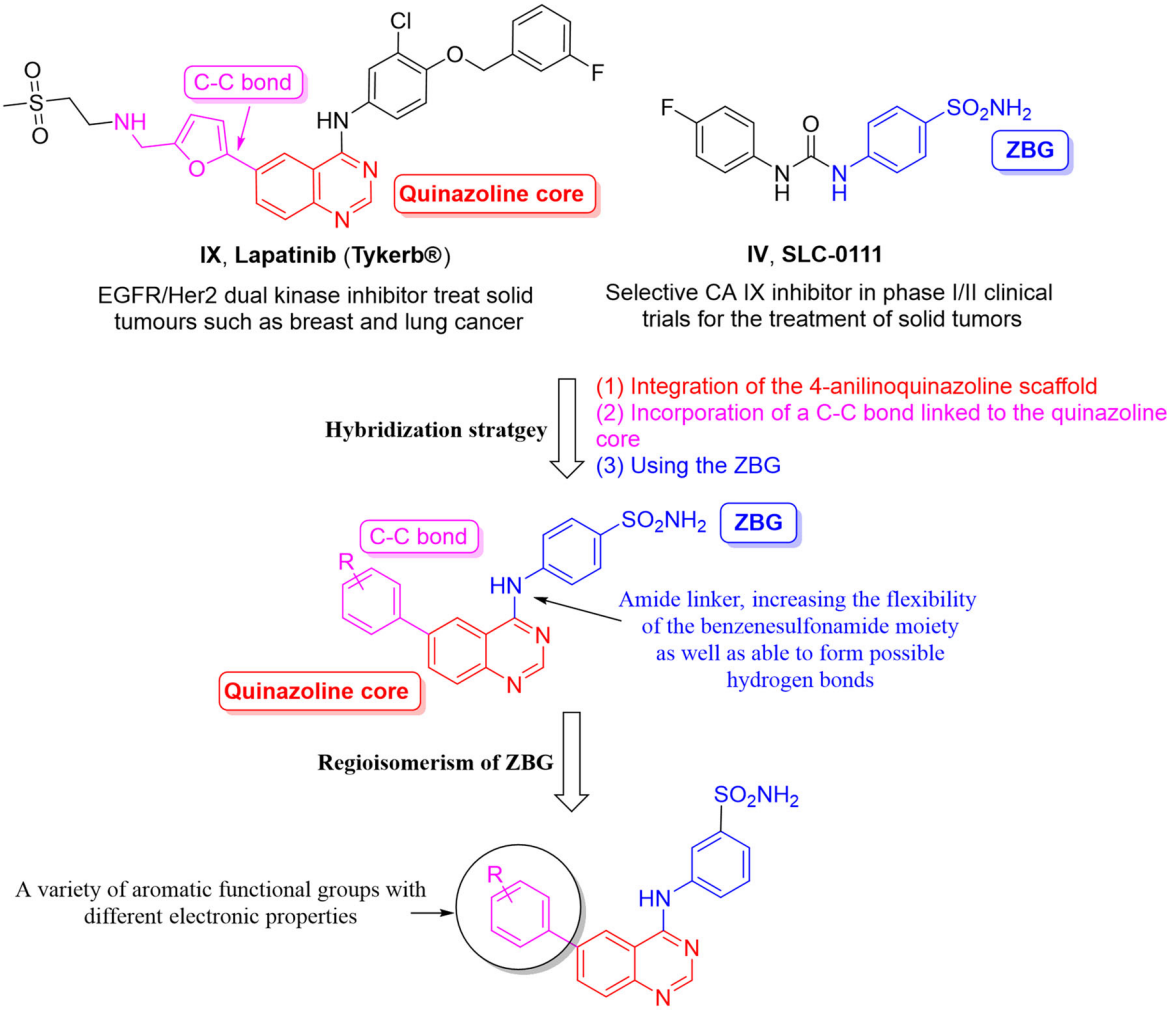
Design of the target hCAIs.

Resuming the efforts of developing effective quinazoline-based hCA inhibitors, a new series of novel 4-anilinoquinazoline-based sulphonamides were designed and synthesised (Figure 4). The effect of the aromatic substitutions at positions 4, 6 and 7 of the quinazoline core, as hCA inhibitor, is largely undiscovered. Therefore, a structural hybridisation strategy (Figure 4) was utilised through incorporating the benzenesulfonamide moiety of SLC-0111 (**IV**) at position 4 of the quinazoline ring. We have also incorporated various aromatic substitutions at positions 6 and 7 of the quinazoline scaffold including **3a**, **4a**‒**f**, **4h** and **4j**. These compounds tested the effects of different hydrophilic substitutions as in compounds **4b**, **4d**, **4h** and **4j** as well as the effect of hydrophobic substitutions as in compounds **3a**, **4a**, **4c**, **4e** and **4f**. In addition to the difference in hydrophilicity and size of the substitutions, the substitutions chosen also possessed different electronic properties to establish key interactions within the roomy hCA binding sites. Thereafter, a regioisomerism strategy was applied to afford the *meta*-sulfamoyl ZBG derivatives, regioisomers **3b**, **4i** and **4k**‒**l**. The hybridisation strategy incorporated the C-C bond from Lapatinib (**IX**) to attach the aromatic substitutions to increase the rigidity of the tail region in a similar manner to the Lapatinib. On the other hand, a flexible amino linker was used to link the quinazoline moiety with the sulphonamide moiety to increase the possibility of hydrogen bond formation as well as grant suitable flexibility to the sulphonamide region to bind easily with Zn metal of the active site.

The target compounds designed herein were synthesised, characterised, and assessed for their hCA inhibitory activity against its four isoforms I, II, IX and XII. Then, the structure-activity relationship (SAR) was discussed. A molecular docking study was carried out to increase the understanding of the hCA inhibitory action of the target compounds.

## 2. Materials and methods

### 2.1. Chemistry

#### 2.1.1. General

In the conducted experiments all acquired solvents and reagents were applied without additional purification. Varian 400 MHz spectrometer (Varian Medical Systems, Inc., Palo Alto, CA, USA) was utilised to calculate the ^1^H NMR spectra with chemical shifts being measured in parts per million (ppm) and coupling constants in Hz. The high-resolution electrospray ionisation mass spectrometry (HR-ESIMS) data were assessed utilising a JMS–700 mass spectrometer (Jeol, Japan) or by HR-ESIMS data obtained via a G2 QTOF mass spectrometer. Reaction monitoring was carried out using TLC on 0.25 mm silica plates (E. Merck; silica gel 60 F_254_). Reverse-phase high performance liquid chromatography (RP-HPLC) was employed to determine the purity of the products, with the UV detector of the HPLC being set at 254 nm. The mobile phases employed were: (A) H_2_O containing 0.05% TFA and (B) CH_3_CN. The purity of the final compound was determined using a gradient of 75% B or 100% B in 30 min. The melting points were measured using a Fisherbrand digital melting point apparatus. Compounds **2a**–**b** and **3a**–**d** were synthesised as reported earlier^34^. The final target compounds were synthesised following the reported procedure of Suzuki cross-coupling reaction^34^.

##### 4-((6-phenylquinazolin-4-yl)amino)benzenesulfonamide (4a)

Off-white solid, Yield: 35%, mp: 310‒312 °C, HPLC purity: 15 min, 98.56%, ^1^H NMR (400 MHz, DMSO-*d*_6_) δ 10.14 (s, 1H), 8.85 (s, 1H), 8.66 (s, 1H), 8.21 (d, *J* = 12.0 Hz, 1H), 8.08 (d, *J* = 8.0 Hz, 2H), 7.91–7.80 (m, 5H), 7.55 (t, *J* = 8.0 Hz, 2H), 7.43 (t, *J* = 4.0 Hz, 1H), 7.29 (s, 2H). ^13^C NMR (100 MHz, DMSO-*d*_6_) δ 158.09, 154.66, 149.76, 149.56, 142.71, 139.53, 138.85, 132.58, 129.50, 128.97, 128.47, 127.66, 126.75, 122.08, 120.95, 115.87. HRMS (ESI) *m/z* calculated for C_20_H_17_N_4_O_2_S [M + H]^+^: 377.1067, found: 377.1072.

##### 4-((6–(4-formylphenyl)quinazolin-4-yl)amino)benzenesulfonamide (4b)

Off-white solid, Yield: 46%, mp: 265‒267 °C, HPLC purity: 17 min, 94.26%, ^1^H NMR (400 MHz, DMSO-*d*_6_) δ 10.20 (s, 1H), 10.08 (s, 1H), 8.97 (s, 1H), 8.68 (s, 1H), 8.30 (d, *J* = 8.0 Hz, 1H), 8.13 (d, *J* = 8.0 Hz, 2H), 8.10–8.04 (m, 4H), 7.92 (d, *J* = 8.0 Hz, 1H), 7.84 (d, *J* = 8.0 Hz, 2H), 7.28 (s, 2H). ^13 ^C NMR (100 MHz, DMSO-*d*_6_) δ 193.22, 158.23, 155.18, 150.20, 145.11, 142.58, 139.11, 137.24, 135.85, 132.56, 130.65, 129.19, 128.28, 126.78, 122.21, 115.88. HRMS (ESI) *m/z* calculated for C_21_H_17_N_4_O_3_S [M + H]^+^: 405.1016, found: 405.1021.

##### 4-((6–(4-fluorophenyl)quinazolin-4-yl)amino)benzenesulfonamide (4c)

White solid, Yield: 26%, mp: 325‒327 °C, HPLC purity: 17 min, 95.19%, ^1^H NMR (400 MHz, DMSO-*d*_6_) δ 10.13 (s, 1H), 8.82 (s, 1H), 8.66 (s, 1H), 8.19 (d, *J* = 8.0 Hz, 1H), 8.07 (d, *J* = 8.0 Hz, 2H), 7.95–7.79 (m, 5H), 7.39 (t, *J* = 12.0 Hz, 2H), 7.28 (s, 2H). ^13 ^C NMR (100 MHz, DMSO-*d*_6_) δ 163.87, 161.44, 158.07, 154.70, 149.55, 142.68, 138.98, 137.73, 136.04, 132.48, 129.74, 126.76, 122.08, 120.89, 116.46, 115.84. HRMS (ESI) *m/z* calculated for C_20_H_16_FN_4_O_2_S [M + H]^+^: 395.0973, found: 395.0977.

##### 4-((6–(3-nitrophenyl)quinazolin-4-yl)amino)benzenesulfonamide (4d)

Off-white solid, Yield: 47%, mp: 335‒337 °C, HPLC purity: 17 min, 95.08%, ^1^H NMR (400 MHz, DMSO-*d*_6_) δ 10.21 (s, 1H), 8.96 (s, 1H), 8.68 (s, 2H), 8.34–8.30 (m, 8.7 Hz, 3H), 8.07 (d, *J* = 8.0 Hz, 2H), 7.93 (d, *J* = 8.0 Hz, 1H), 7.87–7.81 (m, 3H), 7.29 (s, 2H). HRMS (ESI) *m/z* calculated for C_20_H_16_N_5_O_4_S [M + H]^+^: 422.0918, found: 422.0909.

##### 4-((6–(4-methoxyphenyl)quinazolin-4-yl)amino)benzenesulfonamide (4e)

Off-white solid, Yield: 40%, mp: 327‒329 °C, HPLC purity: 17 min, 96.57%, ^1^H NMR (400 MHz, DMSO-*d*_6_) δ 10.10 (s, 1H), 8.78 (s, 1H), 8.64 (s, 1H), 8.17 (d, *J* = 8.0 Hz, 1H), 8.07 (d, *J* = 12.0 Hz, 2H), 7.87‒7.80 (m, 5H), 7.27 (s, 2H), 7.10 (d, *J* = 8.0 Hz, 2H), 3.82 (s, 3H). ^13 ^C NMR (100 MHz, DMSO-*d*_6_) δ 159.81, 157.97, 154.33, 149.23, 142.77, 138.90, 138.51, 132.25, 131.81, 128.81, 126.74, 122.04, 119.89, 115.91, 114.94, 55.74. HRMS (ESI) *m/z* calculated for C_21_H_19_N_4_O_3_S [M + H]^+^: 407.1172, found: 407.1171.

##### 4-((6-(thiophen-3-yl)quinazolin-4-yl)amino)benzenesulfonamide (4f)

Off-white solid, Yield: 40%, mp: 259‒262 °C, HPLC purity: 15 min, 97.18%, ^1^H NMR (400 MHz, DMSO-*d*_6_) δ 10.04 (s, 1H), 8.84 (s, 1H), 8.63 (s, 1H), 8.27 (d, *J* = 8.0 Hz, 1H), 8.07 (d, *J* = 8.0 Hz, 3H), 7.87–7.72 (m, 5H), 7.28 (s, 2H). ^13 ^C NMR (100 MHz, DMSO-*d*_6_) δ 157.96, 154.44, 149.43, 142.68, 140.98, 138.97, 133.82, 132.13, 128.92, 128.01, 126.86, 122.73, 122.08, 119.76, 115.88. HRMS (ESI) *m/z* calculated for C_18_H_15_N_4_O_2_S_2_ [M + H]^+^: 383.0631, found: 383.0626.

##### 3-((6–(4-fluorophenyl)quinazolin-4-yl)amino)benzenesulfonamide (4g)

White solid, Yield: 54%, mp: 277‒279 °C, HPLC purity: 16 min, 100%, ^1^H NMR (400 MHz, DMSO-*d*_6_) δ 10.16 (s, 1H), 8.86 (s, 1H), 8.65 (s, 1H), 8.37 (s, 1H), 8.24–8.17 (m, 2H), 7.99–7.86 (m, 3H), 7.65–7.57 (m, 2H), 7.42 (t, *J* = 8.0 Hz, 4H). ^13 ^C NMR (100 MHz, DMSO-*d*_6_) δ 163.86, 161.42, 158.16, 154.75, 149.49, 144.89, 140.03, 137.67, 136.06, 132.38, 129.76, 128.96, 121.10, 120.87, 119.49, 116.46, 116.24, 115.75. HRMS (ESI) *m/z* calculated for C_20_H_16_FN_4_O_2_S [M + H]^+^: 395.0973, found: 395.0971.

##### 4-((6–(4-morpholinophenyl)quinazolin-4-yl)amino)benzenesulfonamide (4h)

Off- white solid, Yield: 42%, mp: 227‒229 °C, HPLC purity: 15 min, 95.27%, ^1^H NMR (400 MHz, DMSO-*d*_6_) δ 10.12 (s, 1H), 8.78 (s, 1H), 8.65 (s, 1H), 8.20 (d, *J* = 12.0 Hz, 1H), 8.11 (d, *J* = 12.0 Hz, 2H), 7.87–7.81 (m, 5H), 7.30 (s, 2H), 7.12 (d, *J* = 8.0 Hz, 2H), 3.79–3.77 (m, 4H), 3.23–3.21 (s, 4H).^13^C NMR (100 MHz, DMSO- *d*_6_) δ 157.90, 154.12, 151.50, 149.07, 142.81, 138.86, 131.96, 129.71, 128.81, 128.16, 126.73, 122.03, 119.20, 115.79, 66.46, 48.48. HRMS (ESI) *m/z* calculated for C_24_H_24_N_5_O_3_S [M + H]^+^: 462.1594, found: 462.1592.

##### 3-((6–(5-formylfuran-2-yl)quinazolin-4-yl)amino)benzenesulfonamide (4i)

Off-white solid, Yield: 28%, mp: 260‒262 °C, HPLC purity: 13 min, 95.09%, ^1^H NMR (400 MHz, DMSO-*d*_6_) δ 10.38 (s, 1H), 9.69 (s, 1H), 9.08 (s, 1H), 8.67 (s, 1H), 8.35 (s, 2H), 8.19 (s, 1H), 7.92 (d, *J* = 12.0 Hz, 1H), 7.77 (d, *J* = 4.0 Hz, 1H), 7.62 (s, 2H), 7.48–7.39 (m, 3H). HRMS (ESI) *m/z* calculated for C_19_H_15_N_4_O_4_S [M + H]^+^: 395.0809, found: 395.0804.

##### 4-((7–(3-hydroxyphenyl)quinazolin-4-yl)amino)benzenesulfonamide (4j)

Yellow solid, Yield: 22%, mp: 312‒314 °C, HPLC purity: 13 min, 96.82%, ^1^H NMR (400 MHz, DMSO-*d*_6_) δ 10.10 (s, 1H), 9.67 (s, 1H), 8.72–8.64 (m, 2H), 8.14 (d, *J* = 12.0 Hz, 2H), 7.97 (d, *J* = 12.0 Hz, 2H), 7.85 (d, *J* = 8.0 Hz, 2H), 7.37–7.20 (m, 5H), 6.88 (d, *J* = 8.0 Hz, 1H). ^13 ^C NMR (100 MHz, DMSO-*d*_6_) δ 158.44, 157.87, 155.14, 152.27, 145.32, 145.12, 142.84, 140.48, 140.28, 138.75, 136.71, 136.5, 130.55, 121.68, 118.48, 114.73, 114.22. HRMS (ESI) *m/z* calculated for C_20_H_17_N_4_O_3_S [M + H]^+^: 393.1016, found: 393.1009.

##### 3-((7–(3-hydroxyphenyl)quinazolin-4-yl)amino)benzenesulfonamide (4k)

Off-white solid, Yield: 18%, mp: 302‒304 °C, HPLC purity: 15 min, 99.32%, ^1^H NMR (400 MHz, DMSO-*d*_6_) δ 10.08 (s, 1H), 9.68 (s, 1H), 8.69–8.66 (m, 2H), 8.43 (s, 1H), 8.20 (s, 1H), 7.96 (d, *J* = 8.0 Hz, 2H), 7.62–7.58 (m, 2H), 7.44–7.28 (m, 4H), 7.22 (s, 1H), 6.87 (d, *J* = 8.0 Hz, 1H). HRMS (ESI) *m/z* calculated for C_20_H_17_N_4_O_3_S [M + H]^+^: 393.1016, found: 393.1010.

##### 3-((6-phenylquinazolin-4-yl)amino)benzenesulfonamide (4l)

Off-white powder, Yield: 24%, mp: 327–329 °C, HPLC purity: 14 min, 99.81%, ^1^H NMR (400 MHz, DMSO-*d*_6_) δ 10.16 (s, 1H), 8.89 (s, 1H), 8.65 (s, 1H), 8.37 (s, 1H), 8.24–8.21 (m, 2H), 7.91 (d, *J* = 8.0 Hz, 3H), 7.60–7.57 (m, 4H), 7.50–7.40 (m, 3H). HRMS (ESI) *m/z* calculated for C_20_H_17_N_4_O_2_S [M + H]^+^: 377.1067, found: 377.1066.

### 2.2. Carbonic anhydrase inhibition study

The activity of the CA-catalyzed CO_2_ hydration reaction was evaluated employing an applied photophysics stopped-flow instrument. The indicator, Phenol red (at a concentration of 0.2 mM) was added to the CA-catalyzed CO_2_ hydration reaction after a period of 10–100 s. Phenol red functioned at an absorbance maximum of 557 nm, with 20 mM Hepes (pH 7.4) and 10 mM NaClO_4_ (to retain constant ionic strength)^35^. A CO_2_ concentration ranging between 1.7 and 17 mM was employed to evaluate the kinetic parameters and inhibition constants^33^. A minimum of six traces of the starting 5–10% of the reaction were analysed to measure the initial velocity for each inhibitor. In a similar manner, the uncatalyzed rates were evaluated and subtracted from the overall calculated rates^36^. Stock inhibitor solutions (10 mM) were prepared in distilled–deionized water, and dilutions up to 0.01 nM were prepared with distilled–deionized water after that. Preincubation of the inhibitor enzyme solutions at room temperature to enable the formation of the E-I complex was carried out for 15 min before starting the experiment. The inhibition constants were determined employing PRISM 3, while the kinetic parameters for the uninhibited enzymes were calculated using Lineweaver-Burk plots that comprise the mean of at least three separate measurements with errors ranging to ±5–10% of the stated values^37^^,^^38^.

### 2.3. Molecular docking study

The crystal structures of carbonic anhydrase isoforms hCA I (PDB code: 6Y00, resolution 1.37 Å)^39^, hCA II (PDB ID: 4BF1, resolution 1.35 Å)^40^, hCA IX (PDB code: 4Z0Q, resolution 1.45 Å)^41^ and hCA XII (PDB code: 1JD0, resolution 1.50 Å)^42^ were obtained from the Protein Data Bank (www.pdb.org). The downloaded crystal structure was prepared using the Schrodinger 2021 suite package’s preparation wizard with the default settings and pH value of 7.4. ChemDraw Professional 17.0 was employed to sketch the ligands, which were then exported in the structure data file format (SDF) and sent to the Ligprep module. The Schrodinger Ligprep module was then utilised in the ligand preparation process to further optimise the geometry. Glide’s extra precision module was used to dock the minimised ligands inside the binding cavity of the appropriate crystal structure, generating 10 poses per docked ligand. The pose with the most negative docking score was chosen to display.

## 3. Results and discussion

### 3.1. Chemical synthesis

**Scheme 1.**
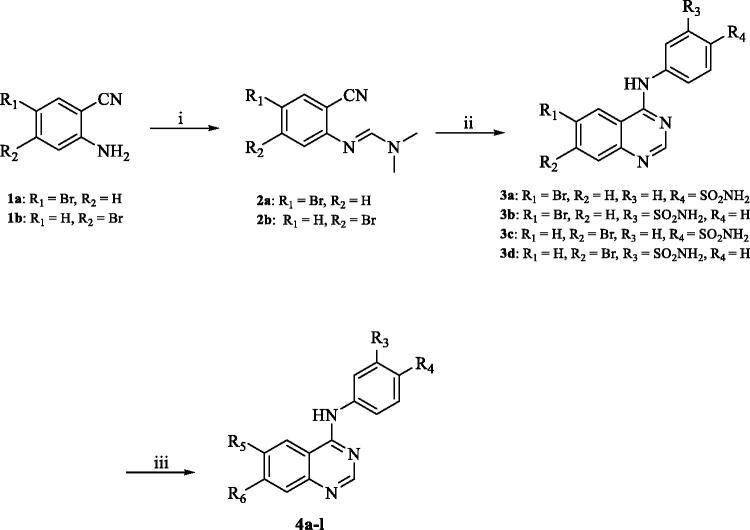
Reagents and conditions: (i) DMF-DMA, 100 °C, 1.5 h; (ii) appropriate aniline, GAA, reflux, 2 h; (iii) suitable boronic acid derivative, Pd(amphos)C12, T3P, dioxane, 80 °C, 2 h (For **4a**–**l**, R_5_ and R_6_ are described in detail in Table 1).

Scheme 1 depicts the synthetic route utilised to produce the target compounds **4a**–**l**. Refluxing of 2-amino-5-bromobenzonitrile (**1a**) or 2-amino-4-bromobenzonitrile (**1b**) with N,N-dimethylformamide dimethyl acetal (DMF-DMA) yielded intermediates **2a**–**b**. Compounds **3a**–**d** were synthesised by reacting the appropriate sulphonamide with the intermediates **2a**–**b** in glacial acetic acid (GAA) under reflux. Using Suzuki reaction compounds **3a**–**d** were subsequently reacted with the suitable boronic acid derivative employing the coupling catalyst Pd(amphos)C12 and the inorganic base T3P (propylphosphonic anhydride) dissolved in dioxane and water at 80 °C under pressure in the presence of nitrogen to produce compounds **4a**–**l**. The chemical structures and purity of the synthesised target compounds were determined using NMR, HRMS, and HPLC analytical techniques^43–45^. The ^1^H NMR spectra of compounds **3a**‒**d** exhibited a characteristic peak at the range of 7.20–7.50 ppm which can be attributed to the sulphonamide NH_2_ and a second peak at 10.10–10.30 ppm attributable to the NH of the amide group verifying the successful synthesis of the intermediates **3a**–**d** from their parent compounds **2a**–**b**. Suzuki cross-coupling reactions success was verified by the presence of the appropriate characteristic peaks in the ^1^H NMR spectra of compounds **4a**–**l**. For example, compound **4b** ^1^H NMR spectra was characterised by the appearance of a new singlet peak at 10.20 ppm attributable to the carbonyl moiety. Similarly, compound **4i** was characterised by the appearance of new singlet peak at 10.38 ppm due to the carbonyl group of the tetrahydrofuran-2-carbaldehyde moiety. In a similar manner, the synthesis of compounds **4j** and **4k** was confirmed by the appearance of a singlet peak at 10.10 and 10.14 ppm, respectively, attributable to the hydroxy group of the phenol moiety. Compound **4e** synthesis was confirmed by the presence of a singlet peak at 3.82 ppm attributable to the three hydrogens of the methoxy group. Likewise, the appearance of two multiple peaks in the aliphatic region of the ^1^H NMR spectrum of compound **4h** attributable to the eight hydrogens of the morpholine ring confirmed its successful synthesis.

### 3.2. Carbonic anhydrase inhibition study

**Figure 5.**
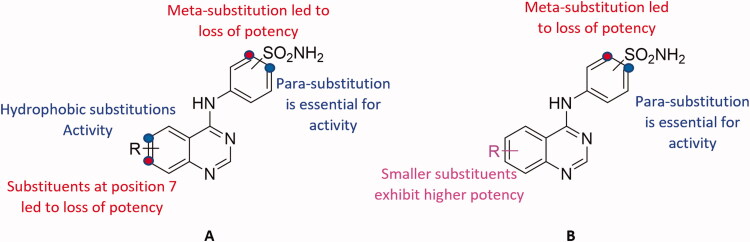
The predicted SAR of the synthesised hybrids against hCA I (A) and hCA II (B) isoforms.

As indicated in Table 1, the biological evaluation of CA inhibitory activities of compounds **3a**–**b**, **4a**–**l** and AAZ as a standard inhibitor, was performed on a panel of four hCA isoforms by a stopped flow CO_2_ hydrase assay. This panel comprised the two ubiquitously expressed cytosolic hCA I and II isoforms, along with the two tumour-related transmembrane hCA IX and XII isoforms. With respect to the first cytosolic isoform (hCA I) the structural hybrid compounds **3a**, **4e**, **4f** showed greater inhibitory activities when compared with the standard AAZ, which possessed an inhibition constant (K_i_) of 250.0 nM, exhibiting inhibitory activity of 89.4, 91.2 and 60.9 nM, respectively. In all three compounds the substitution at the 6- position of the aromatic quinazoline ring was occupied by a hydrophobic substituent and the sulphonamide moiety was in the para position. Interestingly, compound **3a** analogue, Compound **3b,** with a meta-substituted sulphonamide, lost all activity against the first cytosolic isoform hCA I. Similarly, other quinazolines with a hydrophilic “tail” or a meta-substituted sulphonamides lost all inhibitory activity against hCA I. As such the SAR of the synthesised hybrids against the first cytosolic isoform (hCA I) can be summarised in Figure 5(A).

**Table 1.** The inhibitory activity (K_i_) of the synthesised hybrids against hCA I, II, IX and XII isoforms

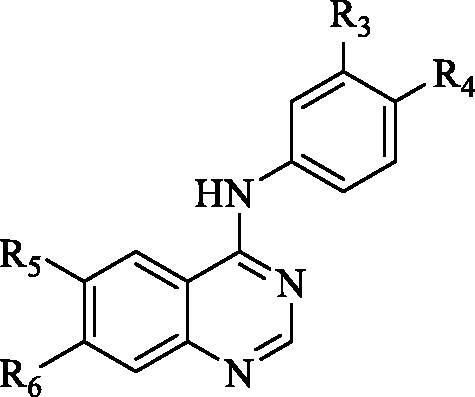								
Cpd	R_3_	R_4_	R_5_	R_6_	K_i_ (nM)			
					hCA I	hCA II	hCA IX	hCA XII
**3a**	H	SO_2_NH_2_	Br	H	89.4	8.7	174.0	46.5
**3b**	SO_2_NH_2_	H	Br	H	7102	5758	95.3	39.4
**4a**	H	SO_2_NH_2_	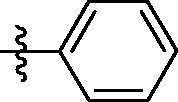	H	600.0	2.4	180.3	65.7
**4b**	H	SO_2_NH_2_	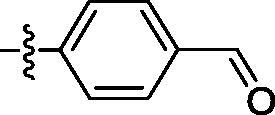	H	650.7	303.8	86.9	67.4
**4c**	H	SO_2_NH_2_	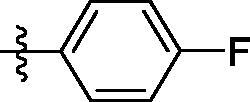	H	931.4	47.1	559.6	93.0
**4d**	H	SO_2_NH_2_	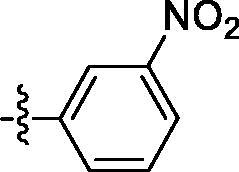	H	588.2	55.7	265.3	90.7
**4e**	H	SO_2_NH_2_	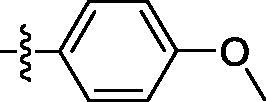	H	91.2	4.6	86.9	77.0
**4f**	H	SO_2_NH_2_	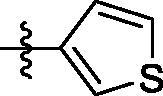	H	60.9	37.1	86.5	89.5
**4g**	SO_2_NH_2_	H	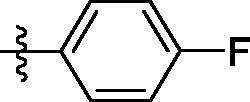	H	3125	231.0	229.0	30.5
**4h**	H	SO_2_NH_2_	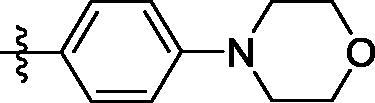	H	548.4	112.3	336.8	49.8
**4i**	SO_2_NH_2_	H	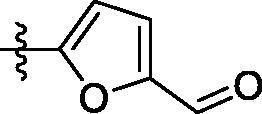	H	5583	2419	487.9	52.7
**4j**	H	SO_2_NH_2_	H	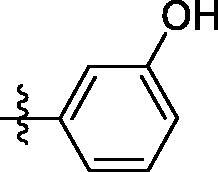	361.3	66.3	244.8	66.7
**4k**	SO_2_NH_2_	H	H	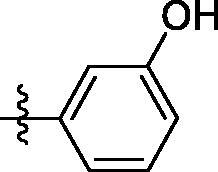	4680	2525	90.3	69.7
**4l**	SO_2_NH_2_	SO_2_NH_2_	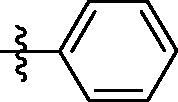	H	6565	678.3	88.8	89.4
AAZ	–	–	–	–	250.0	12.1	25.7	5.7

Similarly, compounds **3a**, **4e** and **4f** showed potent inhibition for the physiologically dominant isoform hCA II with inhibitory concentrations of 8.7, 4.6 and 37.1 nM, respectively. Furthermore, compounds **4a**, **4c** and **4j** exhibited inhibitory potent activities of 2.4, 47.1 and 66.3 nM, respectively. These results indicate that the positioning of the sulphonamide group is essential for determining the activity, with para-substituted sulphonamides being essential for activity. This conclusion was further verified by the loss of activity of compounds **3b**, **4k** and **4l** when compared to their respective potent analogues **3a**, **4a** and **4j**, respectively. Conversely, the nature of the aromatic substitution at positions 6 and 7 of the quinazoline ring, did not affect activity, with both hydrophilic and hydrophobic substitutions showing promising activity against hCA II isoform. However, the smaller substituents exhibited higher potency when compared to larger ones. The predicted SAR for the synthesised hybrids against hCA II isoform is illustrated in Figure 5(B).

All synthesised hybrid compounds demonstrated significant inhibitory activity for the tumour associated isoforms hCA IX and XII. Among the synthesised compounds, **4f** exhibited the highest potency against hCA IX (K_i_ of 86.5 nM), while compound **3b** was the most potent of the synthesised hybrids against hCA XII, with an inhibitory concentration of 39.4 nM.

### 3.3. Molecular docking study

In computer-aided drug design research, the prediction of binding modes of a ligand and its proposed target, in addition to the correlation of the obtained scores with prospective activity, are all useful applications of molecular docking^46^. Another benefit of molecular docking studies is the ability to predict the effect of specific amino acid mutations on the activity profile of the ligand^47^. Furthermore, visualising the docking study’s consequent interactions assists future ligand modification, leading to compounds possessing improved binding characteristics^48^. Hence, the synthesised hybrids were subjected to a molecular docking study against hCA I, II, IX and XII isozymes to correlate the structural characteristic features with the reported inhibitory activity and examine the binding profile of the synthesised hybrids. Generally, the co-crystalized ligands in hCA isoforms I and II and IX exhibited the typical sulphonamide moiety pattern, where the sulphonamide moiety binds to the zinc(II) ion following the displacement of the metal-bound water molecule to establish the tetrahedral adduct with the zinc atom^49^.

**Figure 6.**
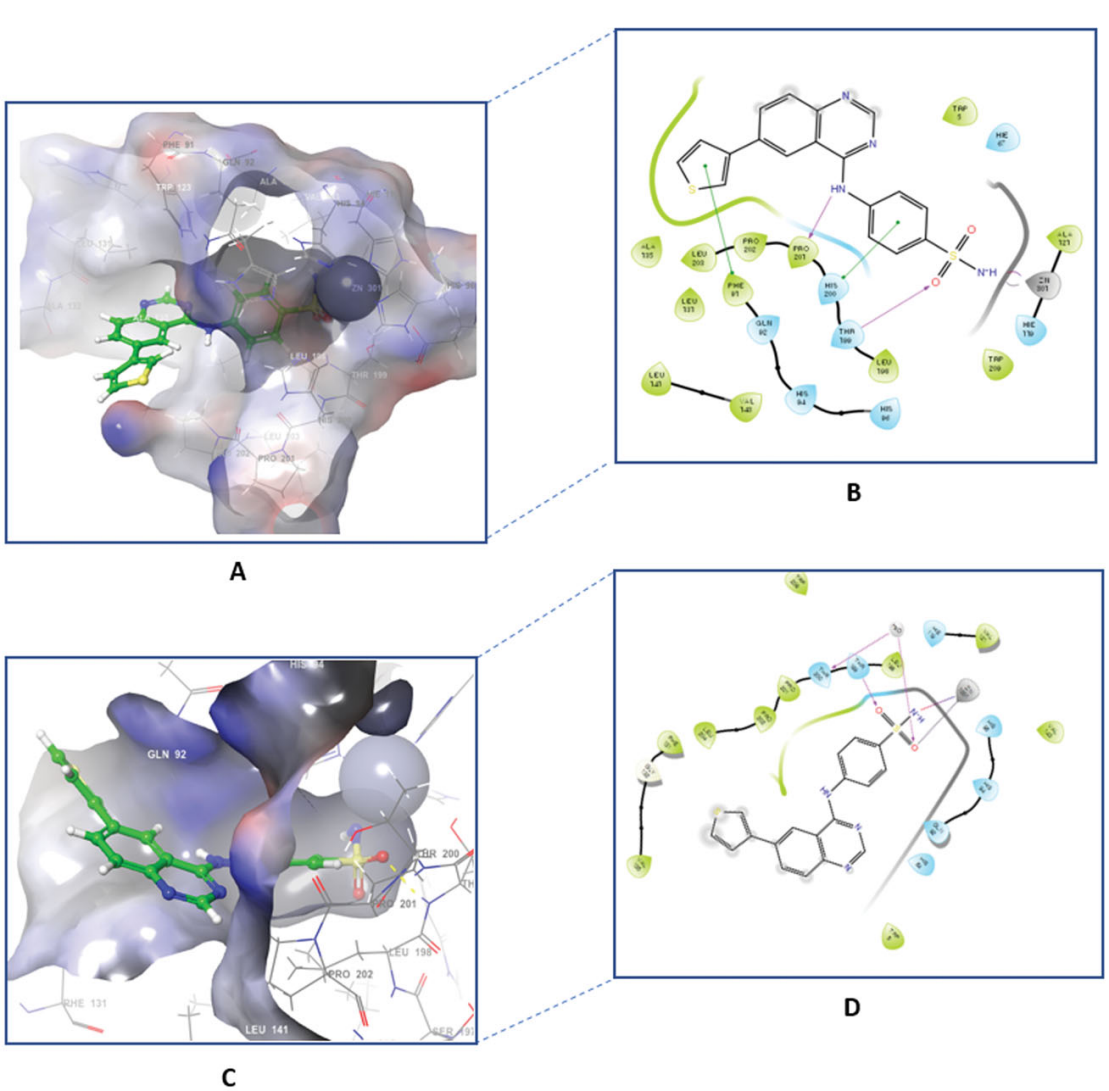
The binding patterns of compound **4f** inside the binding cavity of hCAI and IX. (A) 3D model of the crystal structure of hCA I with compound **4f**. (B) 2D interaction pattern of compound **4f** with hCA I binding cavity. (C) 3D model of the crystal structure of hCA IX with compound **4f**. (D) 2D interaction pattern of compound **4f** with hCA IX binding cavity.

Out of the synthesised hybrids the compounds exhibiting the highest activity, compounds **4a** and **4f,** were subjected to a molecular docking study. Among the synthesised hybrids, compound **4f** exhibited the highest potency against both the hCA I and IX isoforms with inhibitory activity of 60.9 nM and 86.5 nM, respectively. Compound **4f** (60.9 nM), adhered to the general pattern of the benzenesulfonamide ring fitting deeply within the shallow CA active site through the anchoring the zinc atom in a characteristic manner for sulphonamide CAIs through an NH^-^ and Zn^2+^ bond. In addition to the typical zinc metal-ligand interactions compound **4f** formed a hydrogen bond between the carbonyl moiety of the sulphonamide moiety and THR199 of the active site (Figure 6(B)). Compound **4f** exhibited another hydrogen bond between the amide moiety (NH) and PRO201 amino acid residues. Moreover, compound **4f** established two π‒π interactions with PHE91 and HIS200 amino acid residues, further increasing the compound’s affinity to the active site. Compound **4f** was equally potent against hCA IX, which was due to the formation of two metal interactions between the sulphonamide group and the zinc atom of the active site. Furthermore, compound **4f** established a hydrogen bond with THR199 and weak hydrophobic interactions with the amino acid residues of the active site (Figure 6(D)).

**Figure 7.**
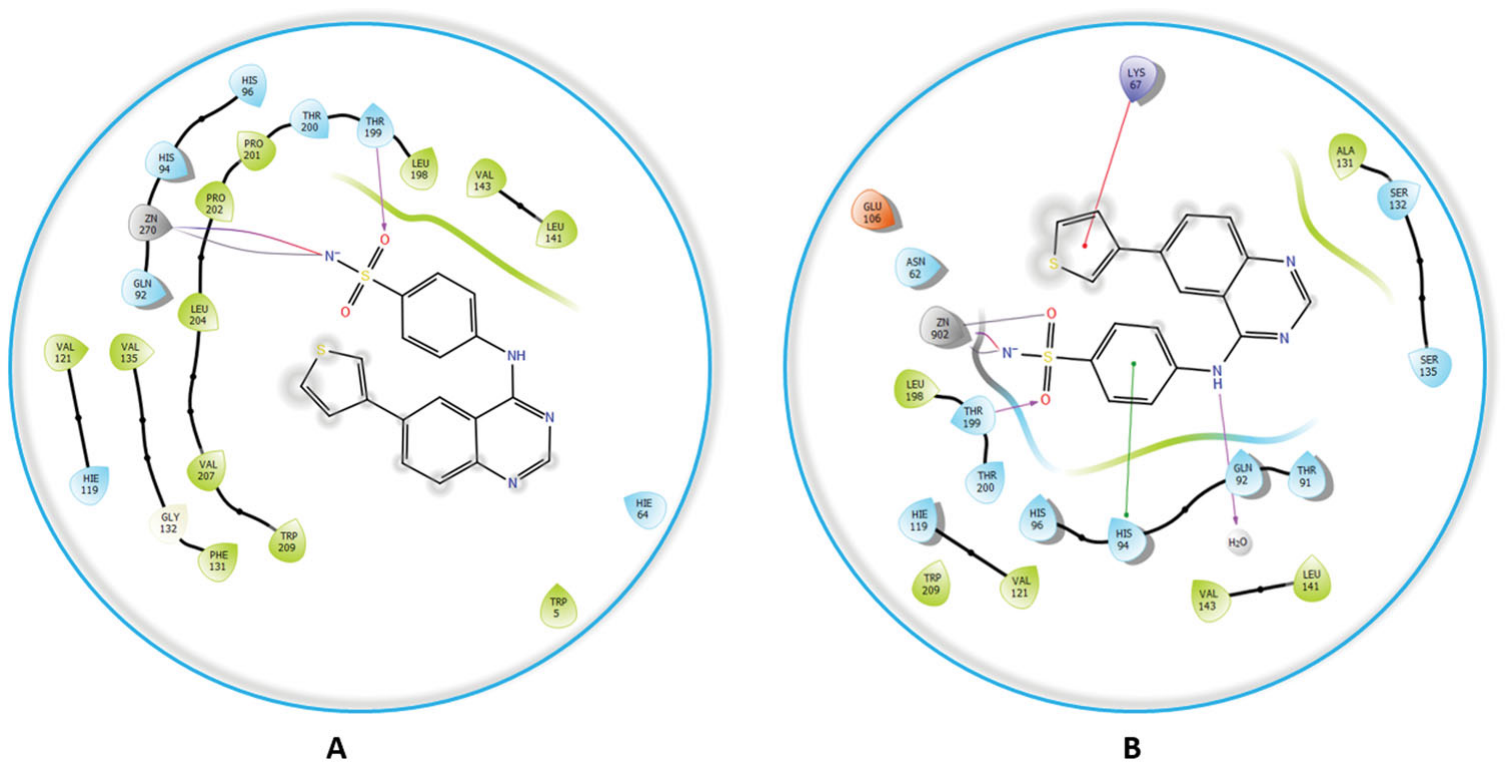
The 2D interaction patterns of compound **4f** with hCA II (A) and XII (B). Favourable interactions are colour coded as follows: purple‒hydrogen bond, grey‒metal bond, red‒π-cation interaction, green‒π‒π stacking interactions. The light green lines represent weak van der Waals interactions.

The docking of compound **4f** against hCA II and XII isoforms revealed a similar interaction pattern, with the sulphonamide moiety fitted deeply inside the binding site groove. Furthermore, compound **4f** established a hydrogen bond interaction with THR199 amino acid residue of hCA II and XII active sites (Figure 7), indicating that the formation of a hydrogen bond with THR199 is essential for the activity of carbonic anhydrase inhibitors. The potent inhibitory activity of compound **4f** (K_i_ = 89.5 nM) can be further explained through the compound’s ability to establish an additional π-cation bond with LYS67 as well as a π-π stacking interaction with HIS94 (Figure 7(B)). The 2D interaction pattern of compound **4f** with hCA II and XII is illuminated in Figure 7, while the corresponding 3D models are demonstrated in the Supplementary file (Figure S4.1‒2).

**Figure 8.**
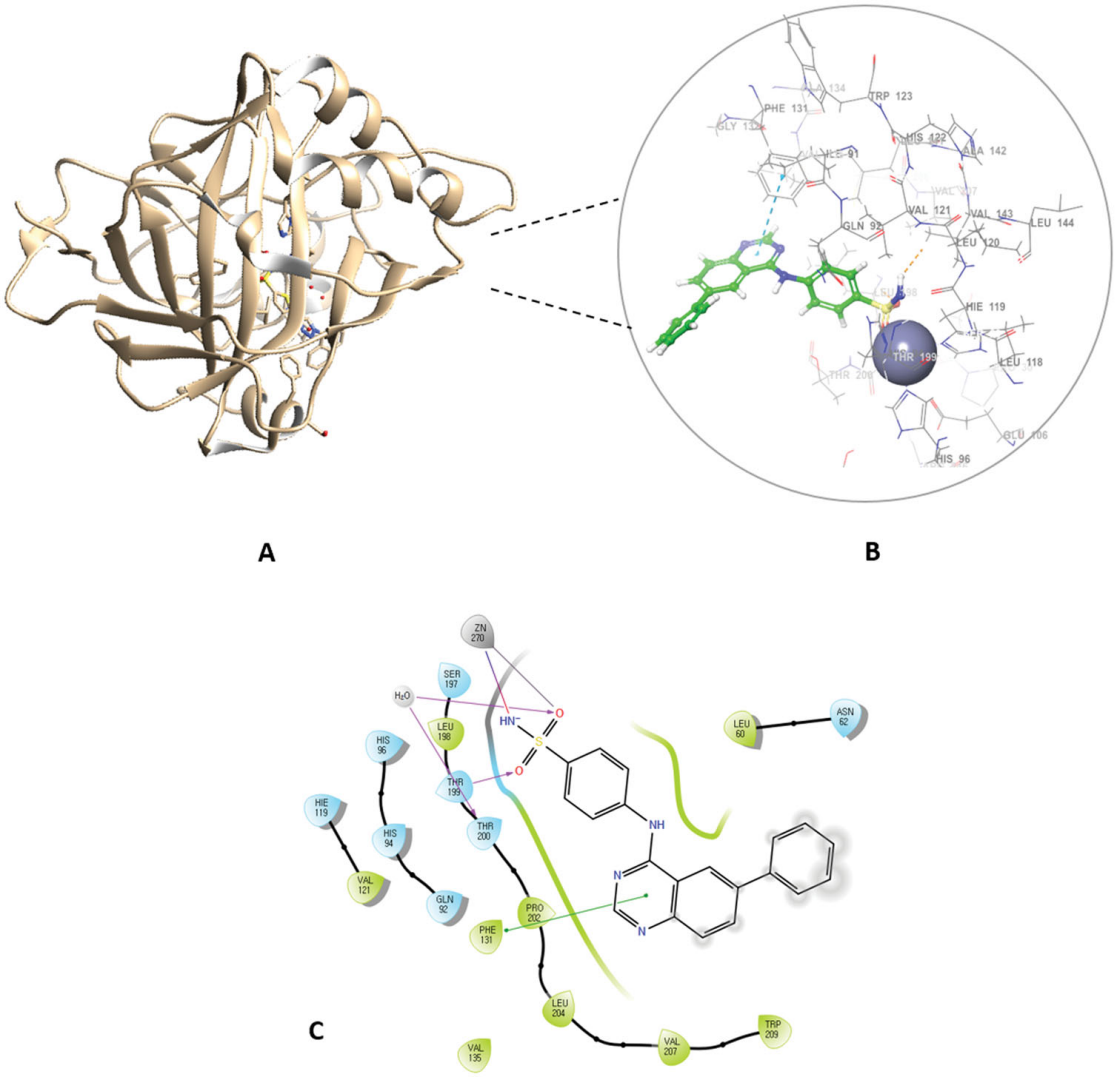
The docked complex of compound **4a** with hCA II isoform. (A) 3D model of the crystal structure of hCA II with compound **4a**, (B) 3D docking pose of compound **4a,** favourable interactions are exhibited as dashed lines: orange‒hydrogen bonds, blue‒π-π stacking, (C) 2D interaction pattern of compound **4a** with hCA II binding cavity, favourable interactions are colour coded as follows: green‒π-π stacking, purple‒hydrogen bonds, grey‒metal interactions and light green‒weak van der Waals interactions.

Comparably, compound **4a,** which possessed the highest potency against the hCA II isoform (K_i_ = 2.4 nM) when docked in the active site hCA II, formed the typical metal interaction of the sulphonamide group with the Zn metal as well as a hydrogen bond with THR199 and π-π stacking interaction between the quinazoline ring and PHE131 (Figure 8). The interaction of compound **4a** against the remaining isoforms is illustrated in the Supplementary file.

## 4. Conclusion

Herein, the design, synthesis, and characterisation of a novel series of quinazoline-based sulphonamides were reported and their activities against hCA I, II, IX and XII isoforms were assessed. Most of the newly synthesised quinazoline sulphonamide hybrids efficiently inhibited the investigated hCA I, II, IX and XII, with inhibitory activity in the single digit nanomolar range. Among the synthesised compounds, compound **4f** possessed the highest potency against hCA I and IX isoforms with a K_i_ of 60.9 nM and 86.5 nM, respectively. Against the hCA II isoform, compound **4a** displayed the highest inhibitory activity of 2.4 nM. Compound **4g** was the most potent inhibitor against hCA XII with K_i_ of 30.5 nM. Additionally, compounds **3a** and **4b** possessed single digit inhibitory activity against hCA II of 8.7 and 4.6 nM, respectively. Compounds **3a** and **4e** were able to inhibit hCA I at concentrations of 89.4 and 91.2 nM, which is more potent when compared to the inhibitory concentration of the standard Acetazolamide (250 nM). Furthermore, all tested compounds displayed potent activity against the hCA XII isoform, with inhibitory activities ranging from 30.5‒93 nM. The SAR of the synthesised hybrids were predicted based on the obtained biological data. Finally, a molecular docking study was conducted to provide insights on the binding interactions of the most potent compound for each tested hCA isoform.

## Supplementary Material

Supplemental Material
