# Supplementary material for: 4-Anilinoquinazoline-based benzenesulfonamides as nanomolar inhibitors of carbonic anhydrase isoforms I, II, IX, and XII: design, synthesis, in-vitro, and in-silico biological studies
Source: J Enzyme Inhib Med Chem. 2022 Mar 30;37(1):994–1004. doi: 10.1080/14756366.2022.2055553 (PMC8973350; doi:10.1080/14756366.2022.2055553)
Supplement: Supplemental Material [file IENZ_A_2055553_SM0917.pdf]

4-Anilinoquinazoline-based Benzenesulfonamides as Nanomolar Inhibitors of Carbonic Anhydrase Isoforms I, II, IX, and XII: Design, Synthesis, *In-vitro*, and *In-silico* Biological Studies

Contents

1. ^{1}HNMR and ^{13}CNMR data of compounds **3a**–**d** and **4a**–**l**

2. Representative HPLC purity data of the compounds **3a**–**d** and **4a**–**l**

3. Representative HRMS data of the compounds **3a**–**d** and **4a**–**l**

4. Supplementary docking figures

1. ^{1}HNMR and ^{13}CNMR data of compounds 3a–d and 4a–f

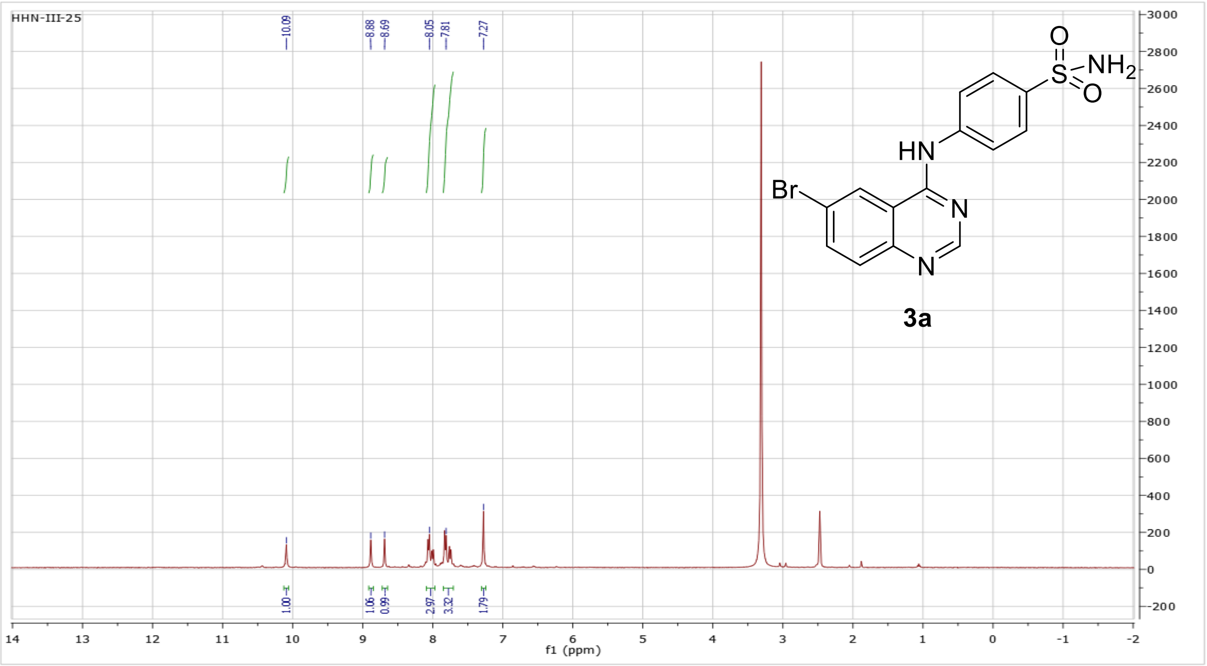

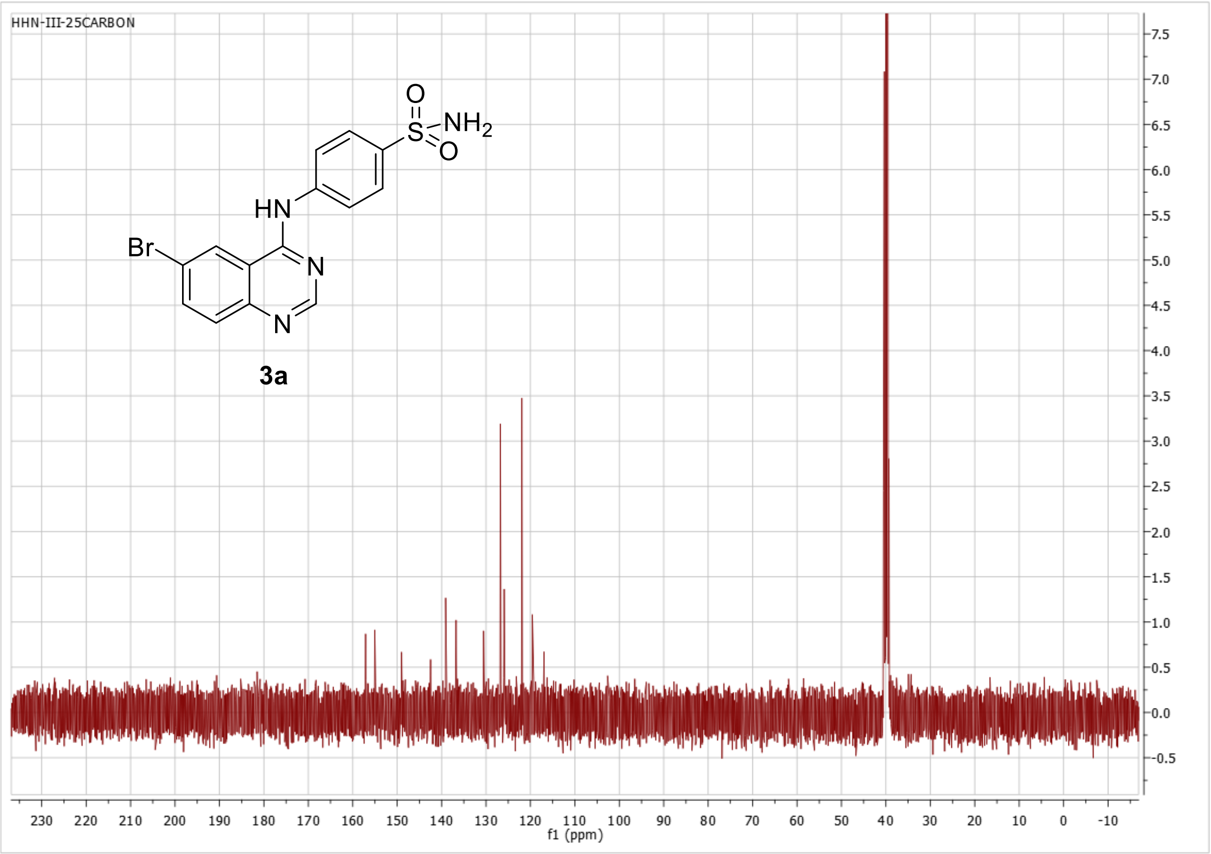

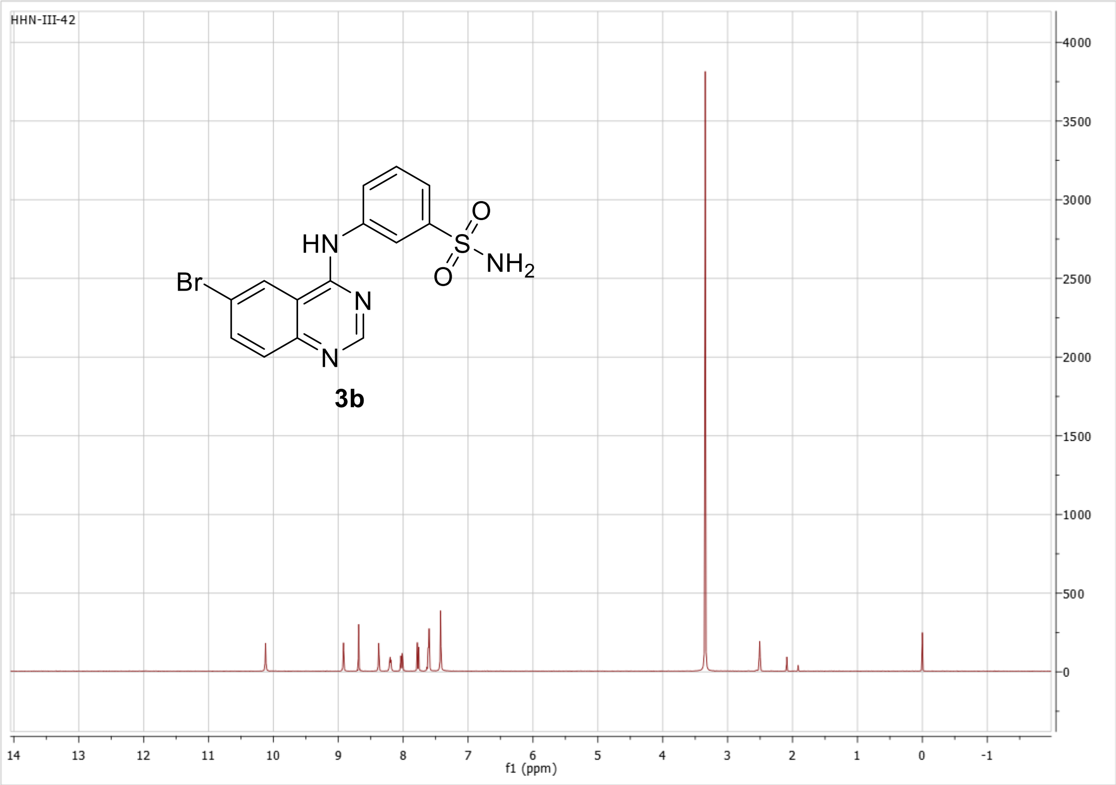
HHN-III-42
3b
Br
HN
N
N
O
S
O
NH2
f1 (ppm)

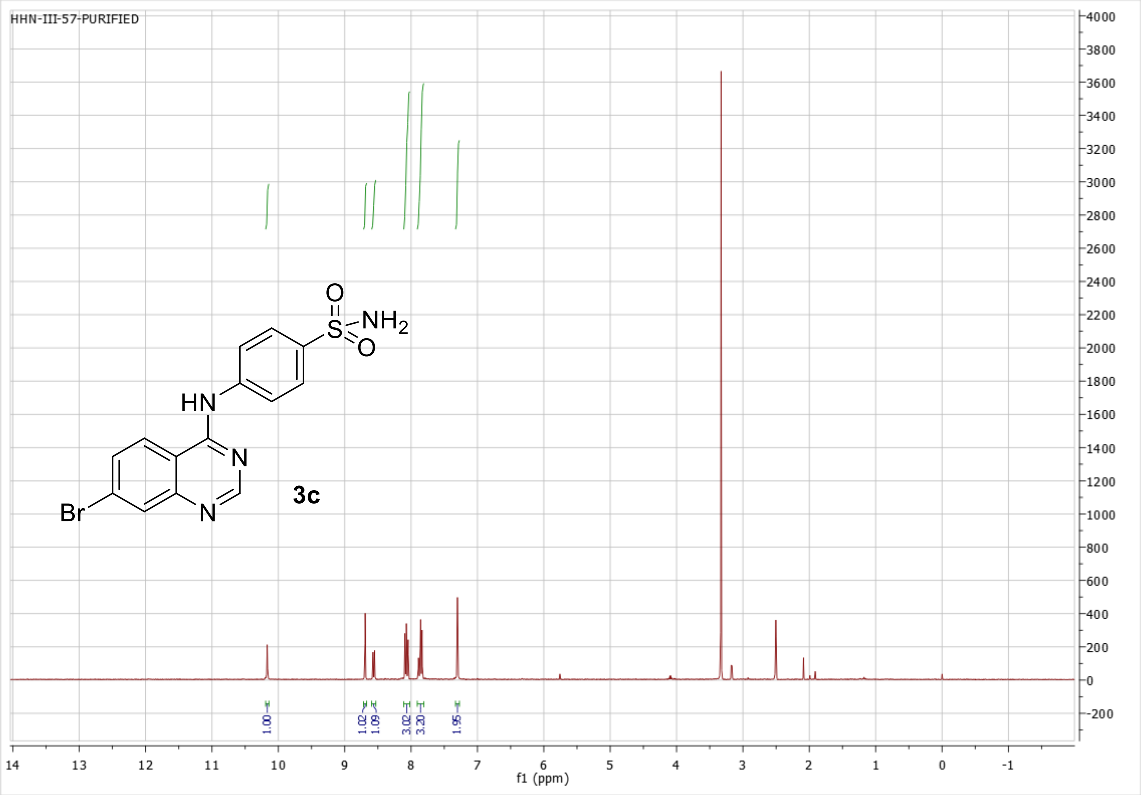
HHN-III-57-PURIFIED
3c
Br
HN
N
N
O
S
O
NH2
1.00
1.02
1.09
3.02
3.20
1.95
f1 (ppm)

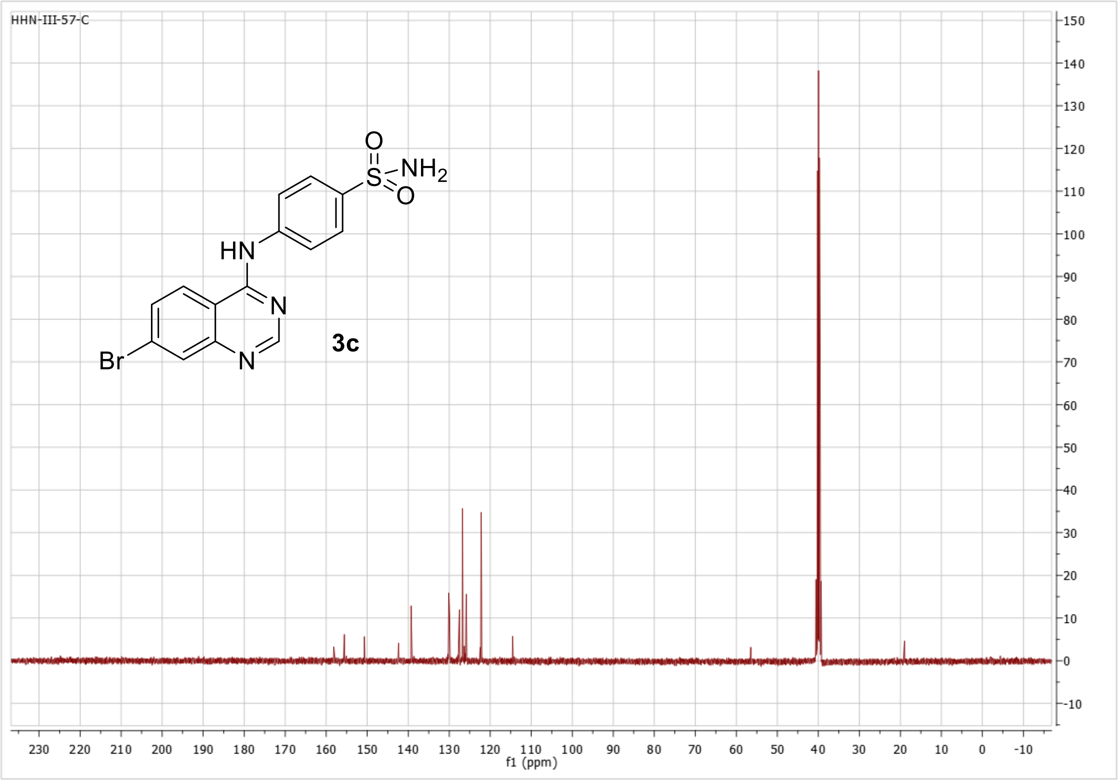
HHN-III-57-C
3c
Br
HN
N
NH2
O
S
f1 (ppm)
230 220 210 200 190 180 170 160 150 140 130 120 110 100 90 80 70 60 50 40 30 20 10 0 -10
150 140 130 120 110 100 90 80 70 60 50 40 30 20 10 0 -10

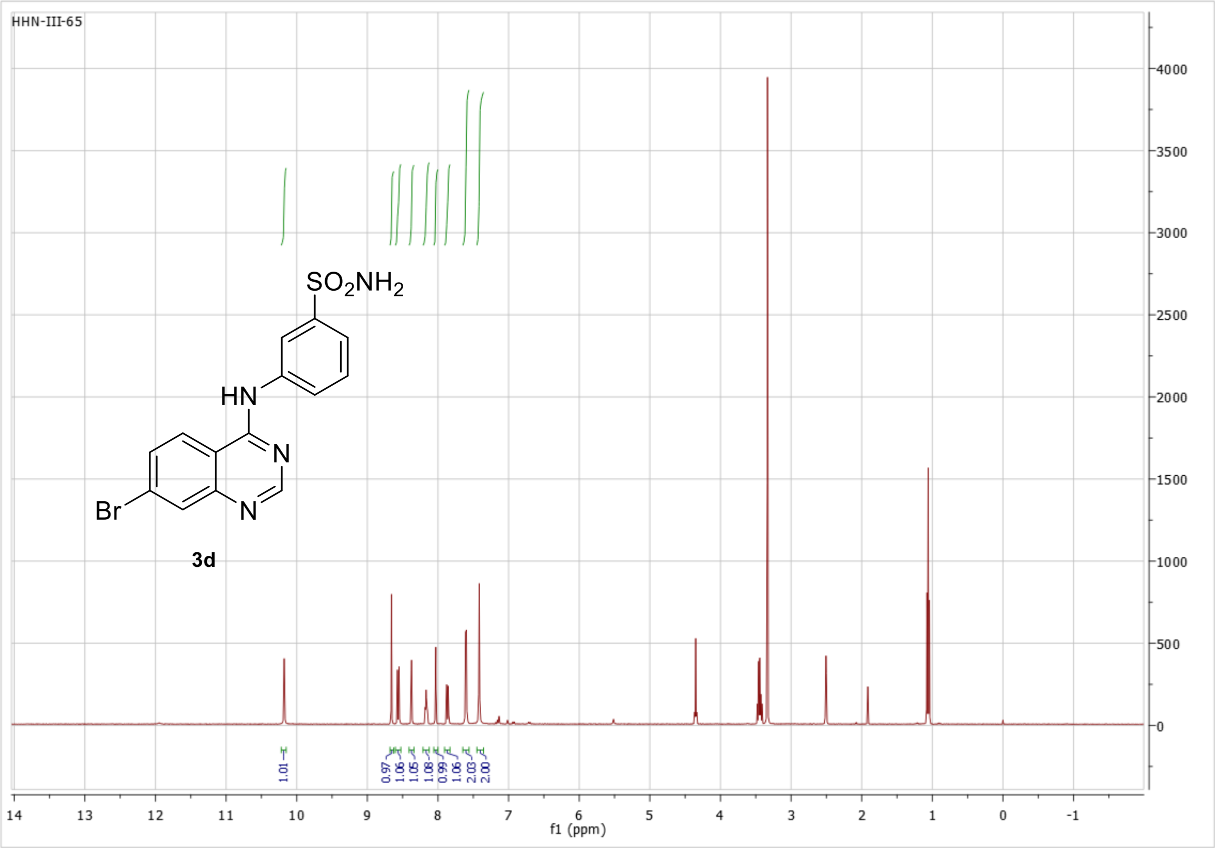
HHN-III-65
SO2NH2
HN
N
Br
3d
1.01
0.97 1.06 1.05 1.08 0.99 1.06 2.03 2.00
f1 (ppm)
14 13 12 11 10 9 8 7 6 5 4 3 2 1 0 -1
4000 3500 3000 2500 2000 1500 1000 500 0

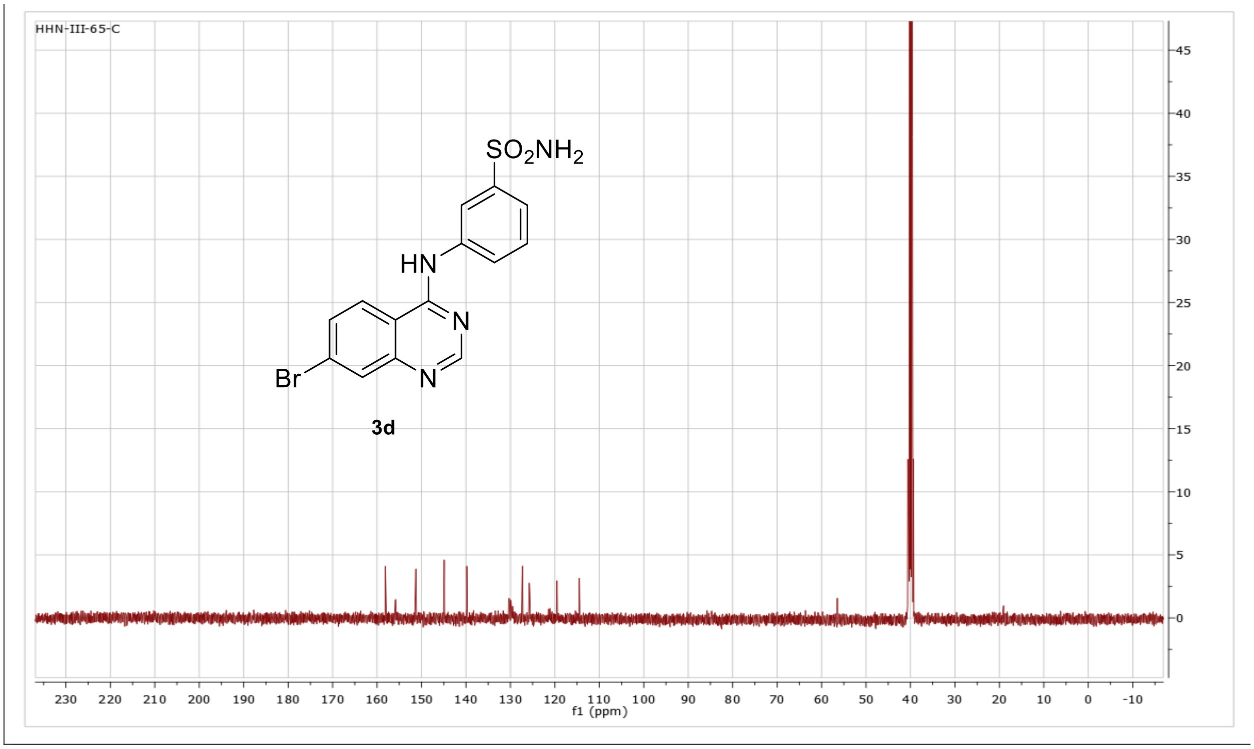
HHN-III-65-C
SO2NH2
HN
N
Br
N
3d
f1 (ppm)

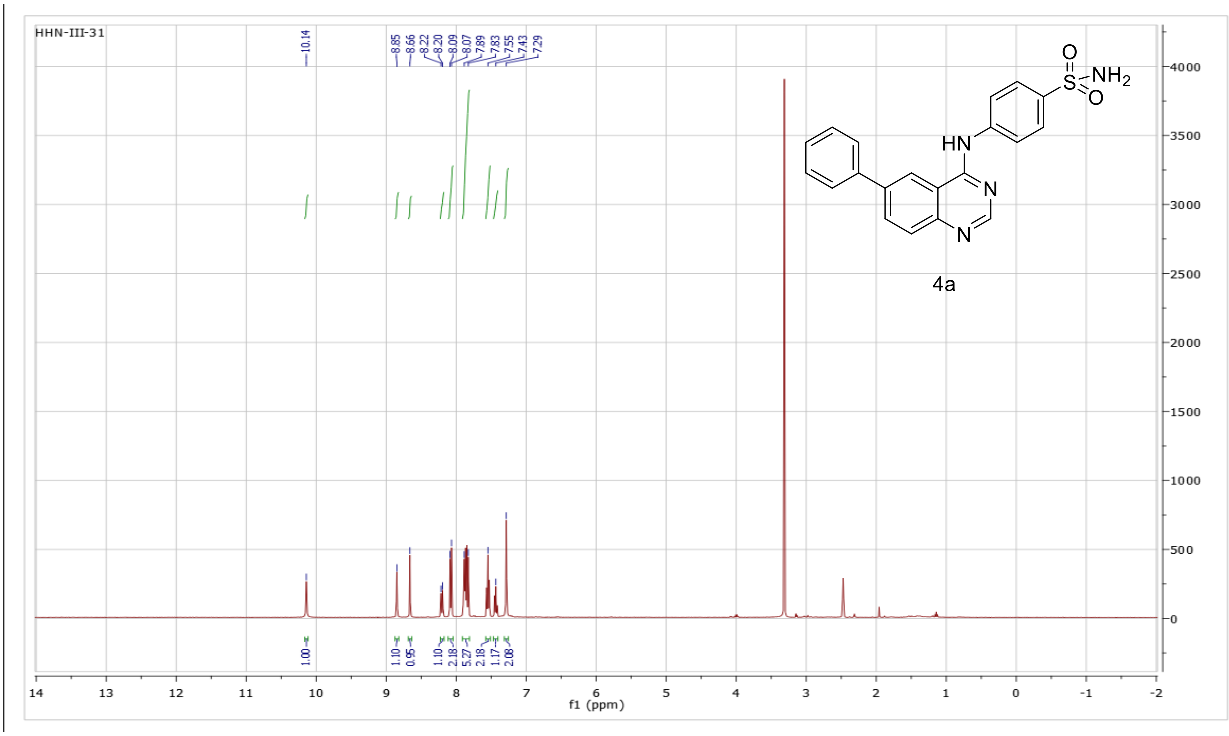
HHN-III-31
10.14
8.85
8.66
8.22
8.20
8.09
8.07
7.89
7.83
7.55
7.43
7.29
1.00
1.10
0.95
1.10
2.18
5.27
2.18
1.17
2.08
O
S
NH2
O
HN
N
N
4a
f1 (ppm)

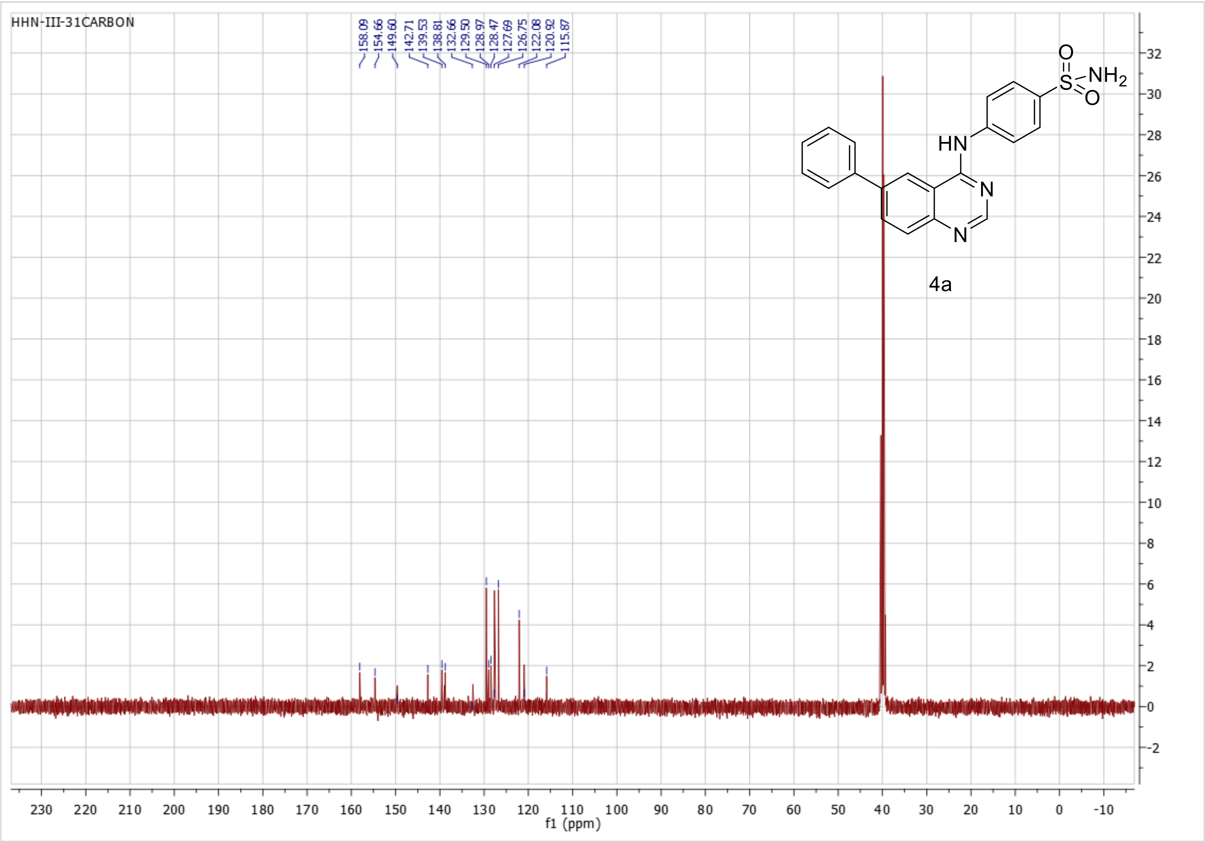
HHN-III-31CARBON
158.09
154.66
149.60
142.71
139.53
138.81
132.66
129.50
128.97
128.47
127.69
126.75
122.08
120.92
115.87
HN
N
N
O
S
NH2
O
4a
f1 (ppm)

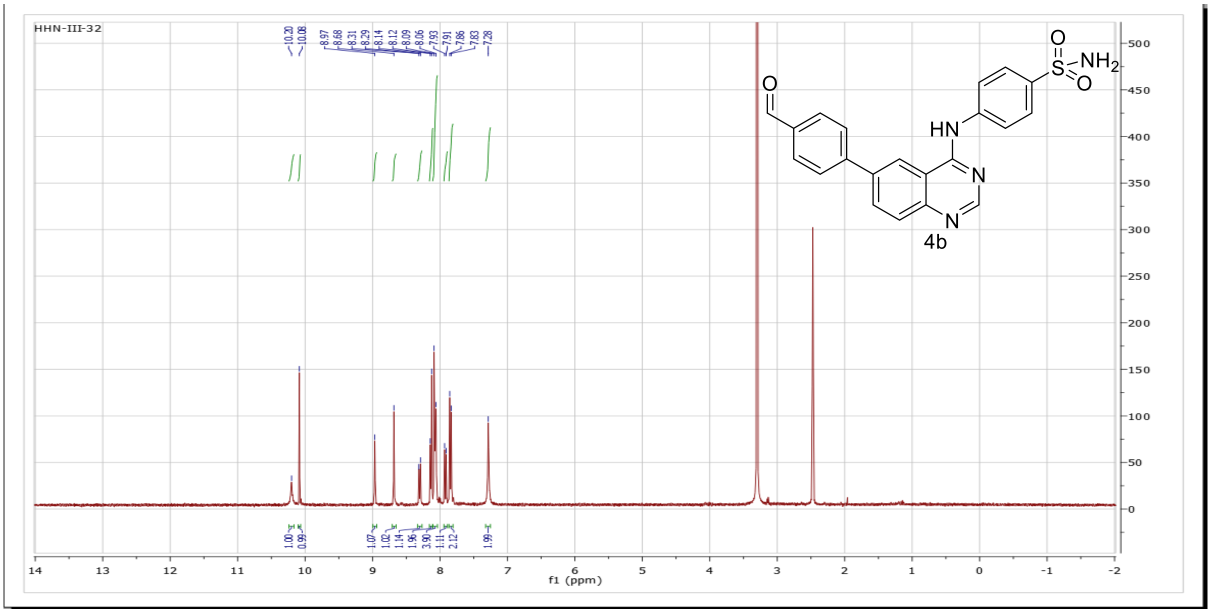
HHN-III-32
10.20
10.08
8.97
8.68
8.31
8.29
8.14
8.12
8.09
8.06
7.93
7.91
7.86
7.83
7.28
O
HN
N
N
O
S
NH2
O
4b
f1 (ppm)

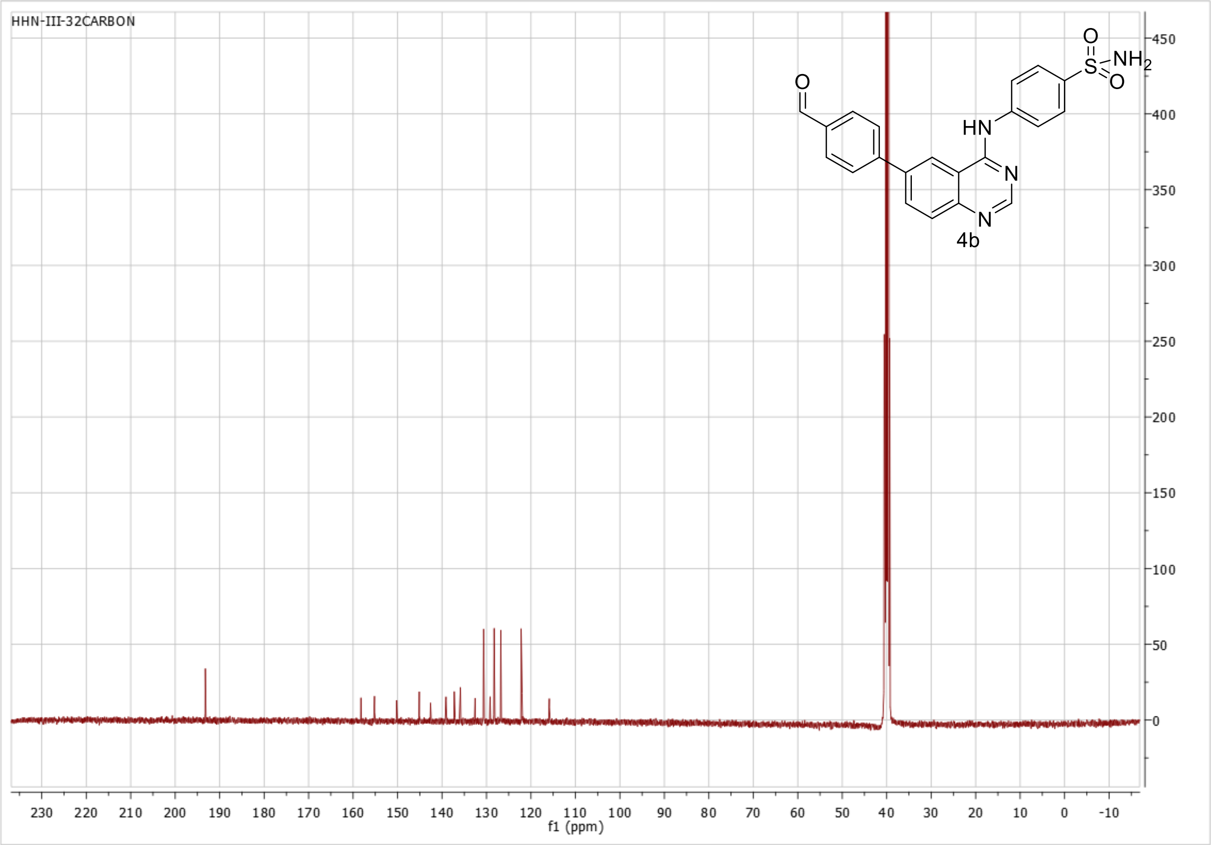
HHN-III-32CARBON
4b
f1 (ppm)

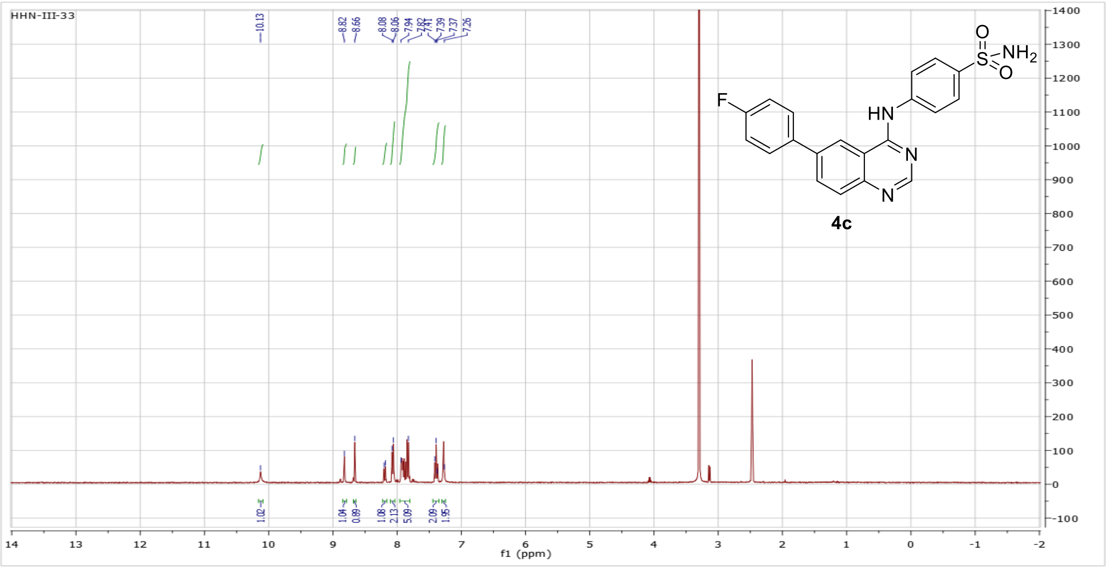
HHN-III-33
10.13
8.82
8.66
8.08
8.06
7.94
7.82
7.41
7.39
7.37
7.26
4c
1.02
1.04
0.99
1.08
2.13
5.09
2.09
1.95
f1 (ppm)

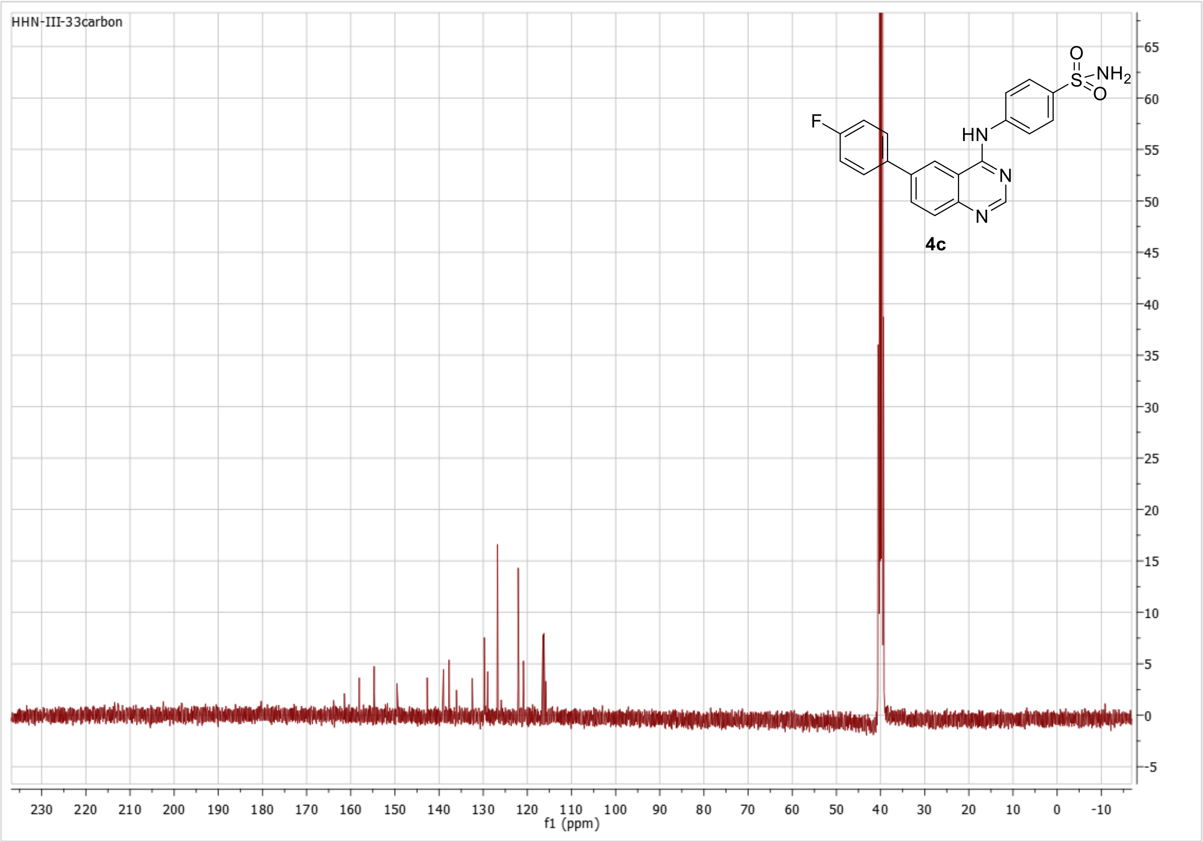
HHN-III-33carbon
4c
f1 (ppm)

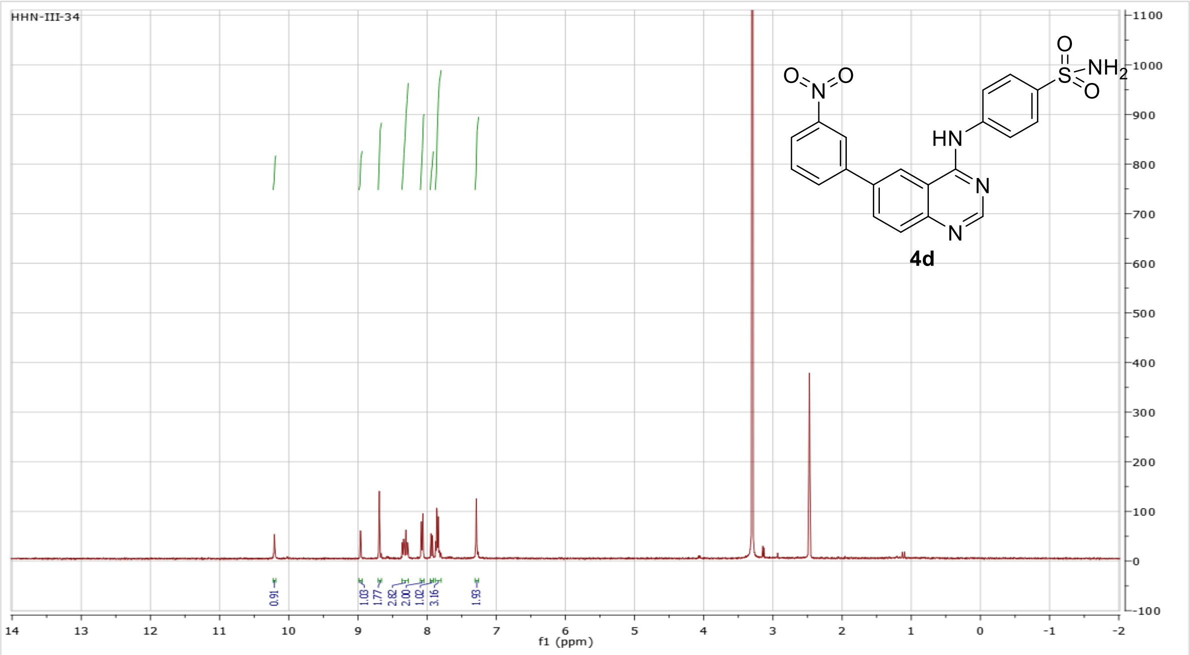
HHN-III-34
4d
0.91
1.03
1.77
2.82
2.00
1.02
3.16
1.93
f1 (ppm)

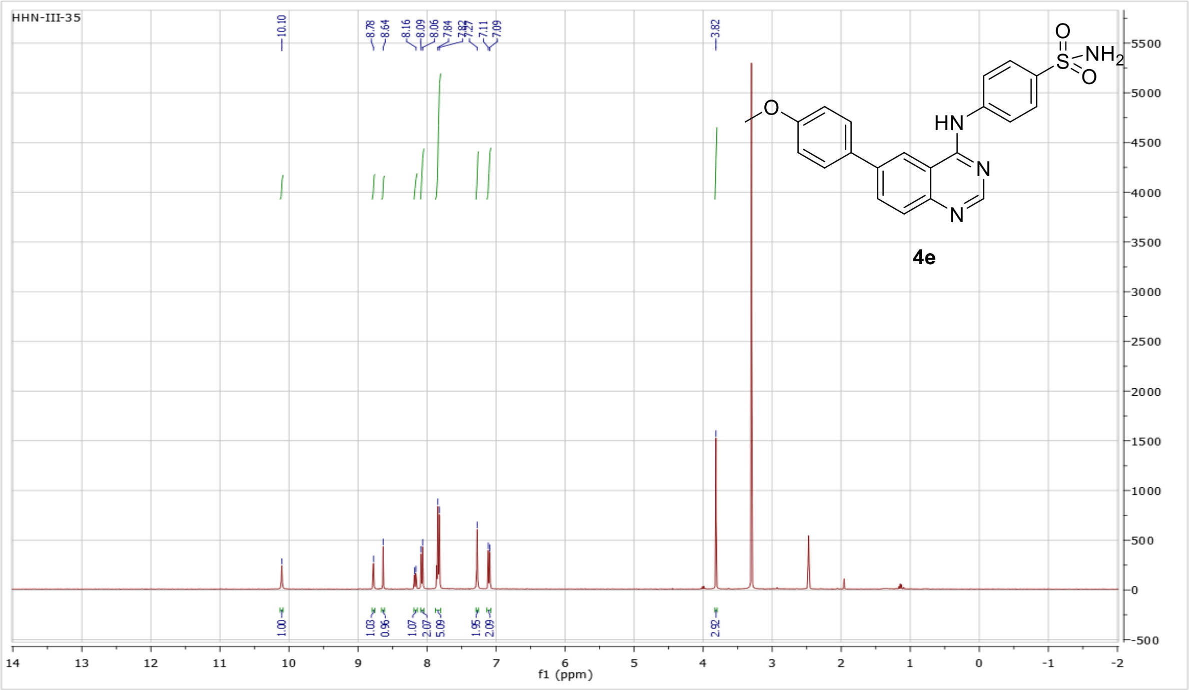
HHN-III-35
10.10
8.78
8.64
8.16
8.09
8.06
7.84
7.82
7.27
7.11
7.09
3.82
1.00
1.03
0.96
1.07
2.07
5.09
1.95
2.09
2.92
f1 (ppm)
4e

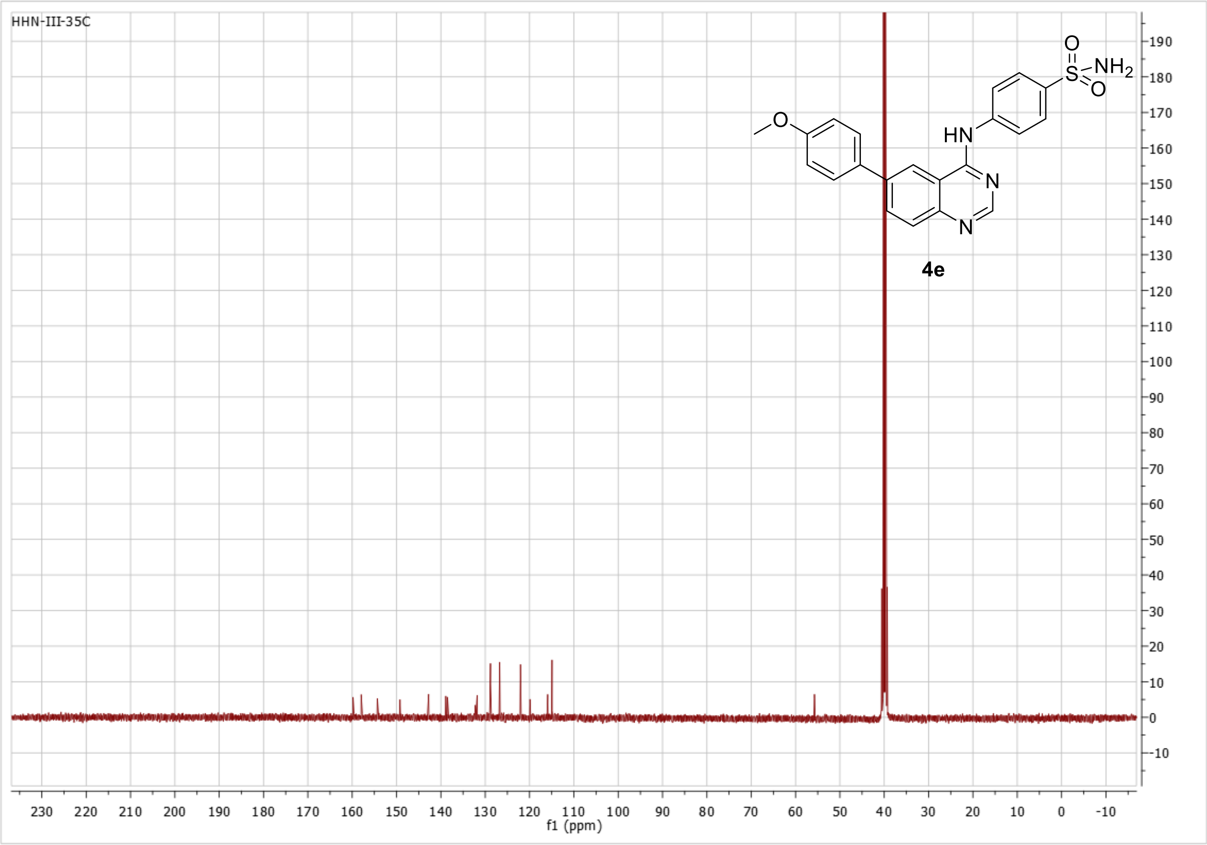
HHN-III-35C
f1 (ppm)
4e

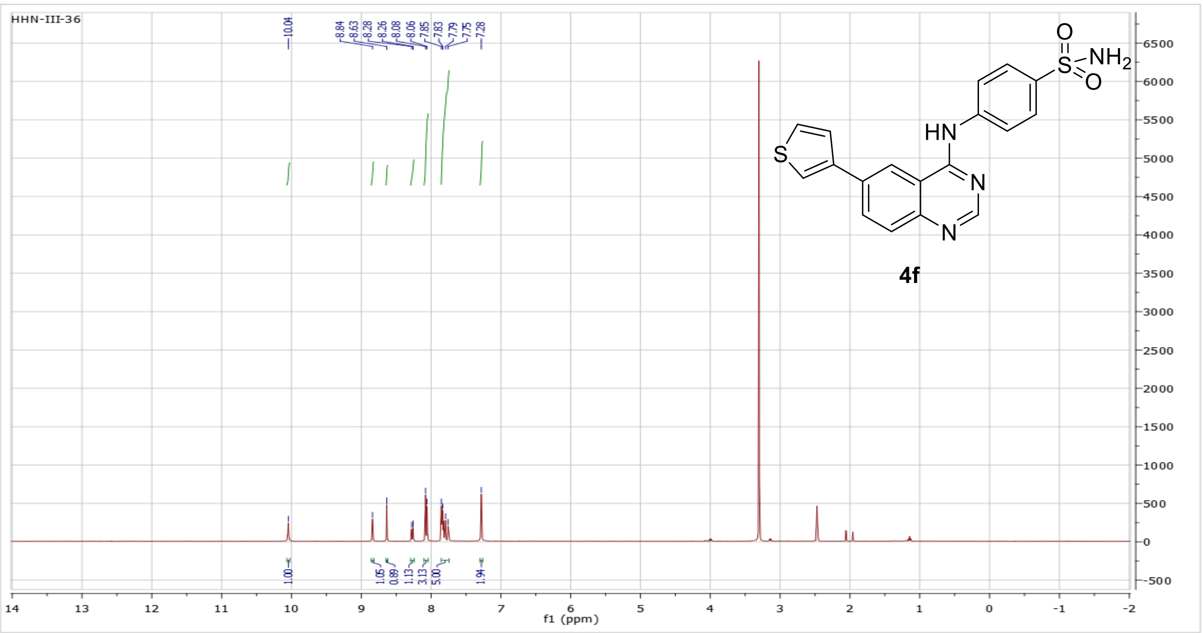
HHN-III-36
10.04
8.84
8.63
8.28
8.26
8.08
8.06
7.85
7.83
7.79
7.75
7.28
1.00
1.05
0.89
1.13
3.13
5.00
1.94
S
HN
N
N
O
S
NH2
O
4f
f1 (ppm)

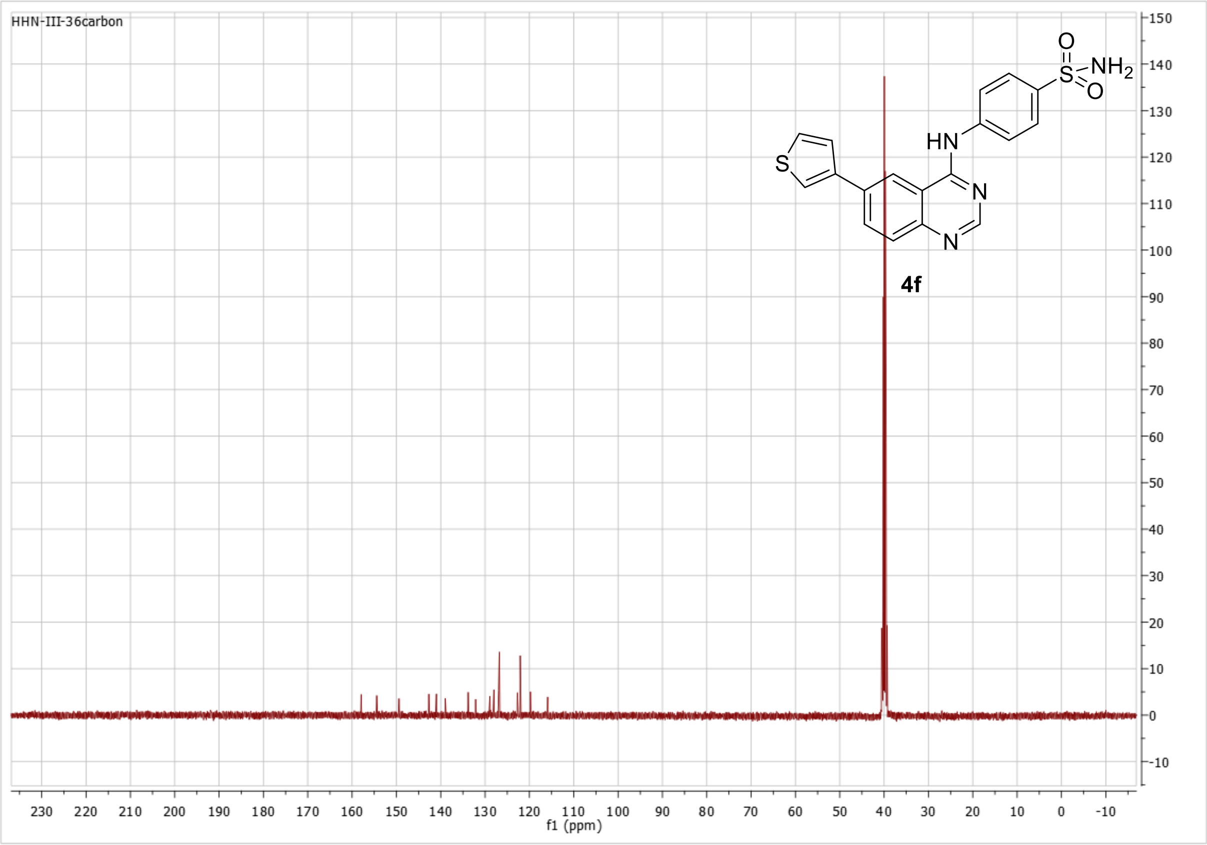
HHN-III-36carbon
S
HN
N
N
O
S
NH2
O
4f
f1 (ppm)

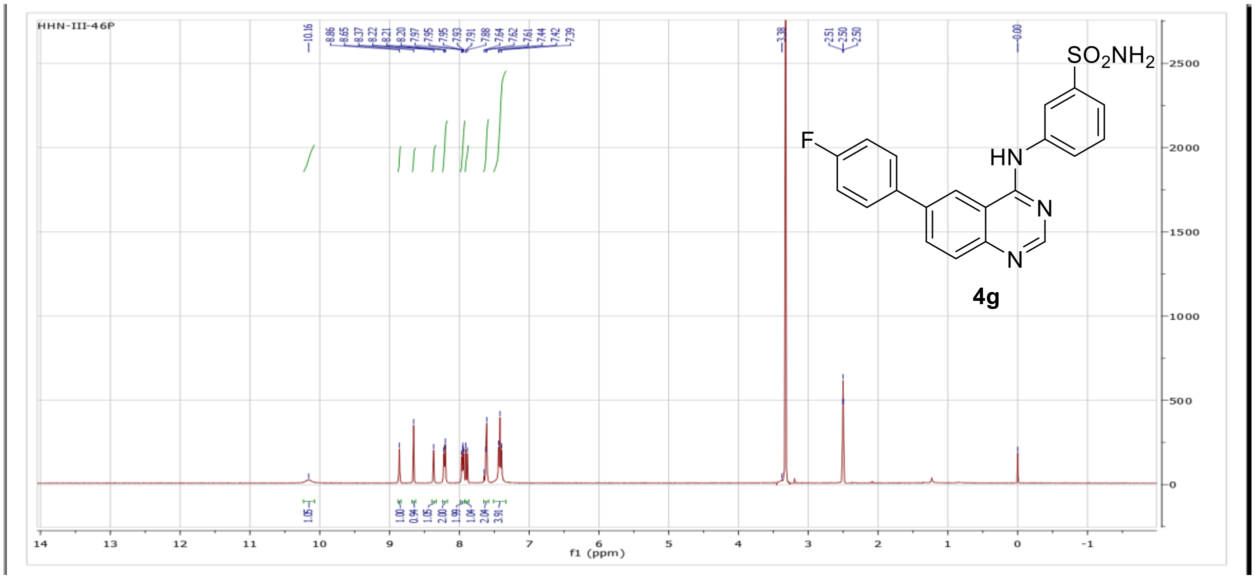
HHN-III-46P
10.16
8.86
8.65
8.37
8.22
8.21
8.20
7.97
7.95
7.95
7.93
7.91
7.88
7.64
7.62
7.61
7.44
7.42
7.39
3.38
2.51
2.50
2.50
0.00
1.05
1.00
0.94
1.05
2.00
1.99
1.04
2.04
3.91
f1 (ppm)
SO2NH2
F
HN
N
N
4g

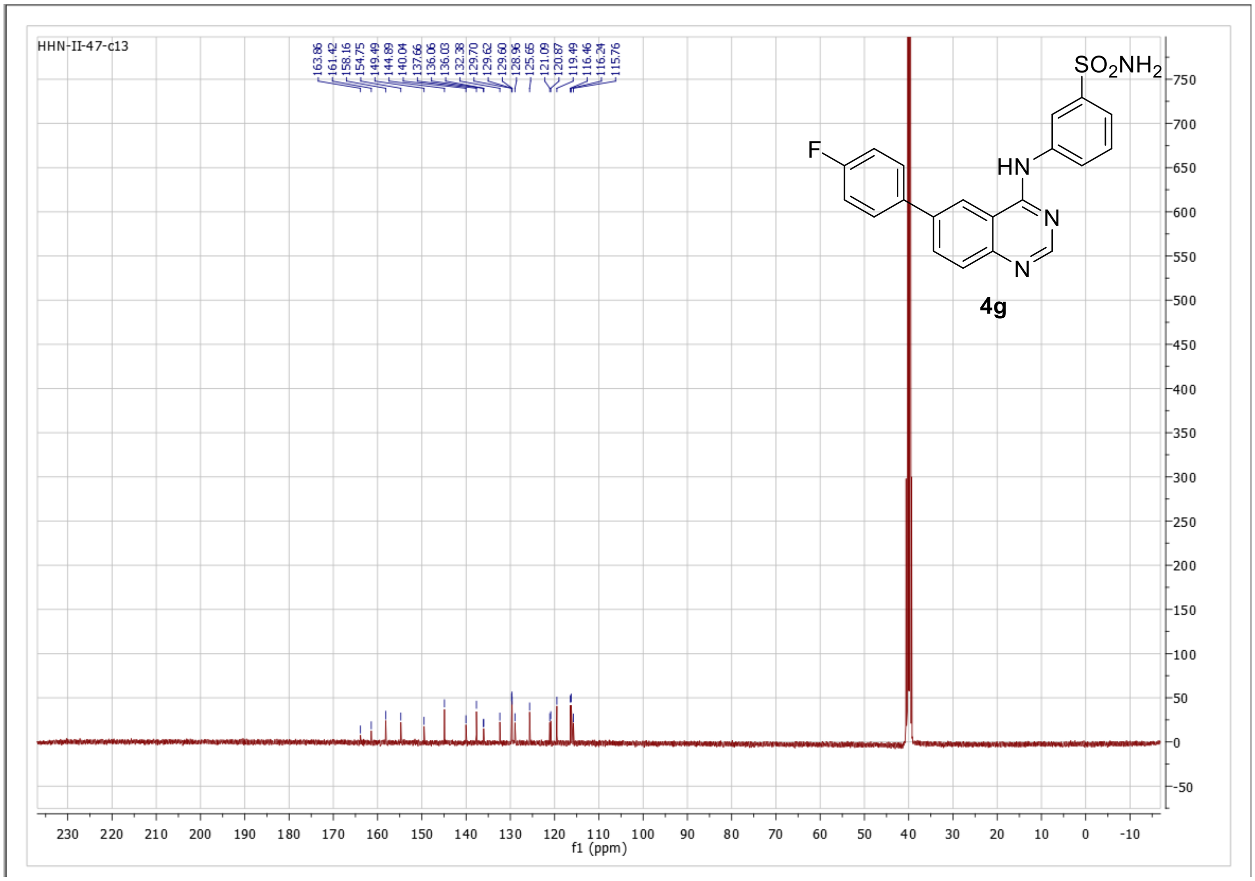
HHN-II-47-c13
163.86
161.42
158.16
154.75
149.49
144.89
140.04
137.66
136.06
136.03
132.38
129.70
129.62
129.60
128.96
125.65
121.09
120.87
119.49
116.46
116.24
115.76
f1 (ppm)
SO2NH2
F
HN
N
N
4g

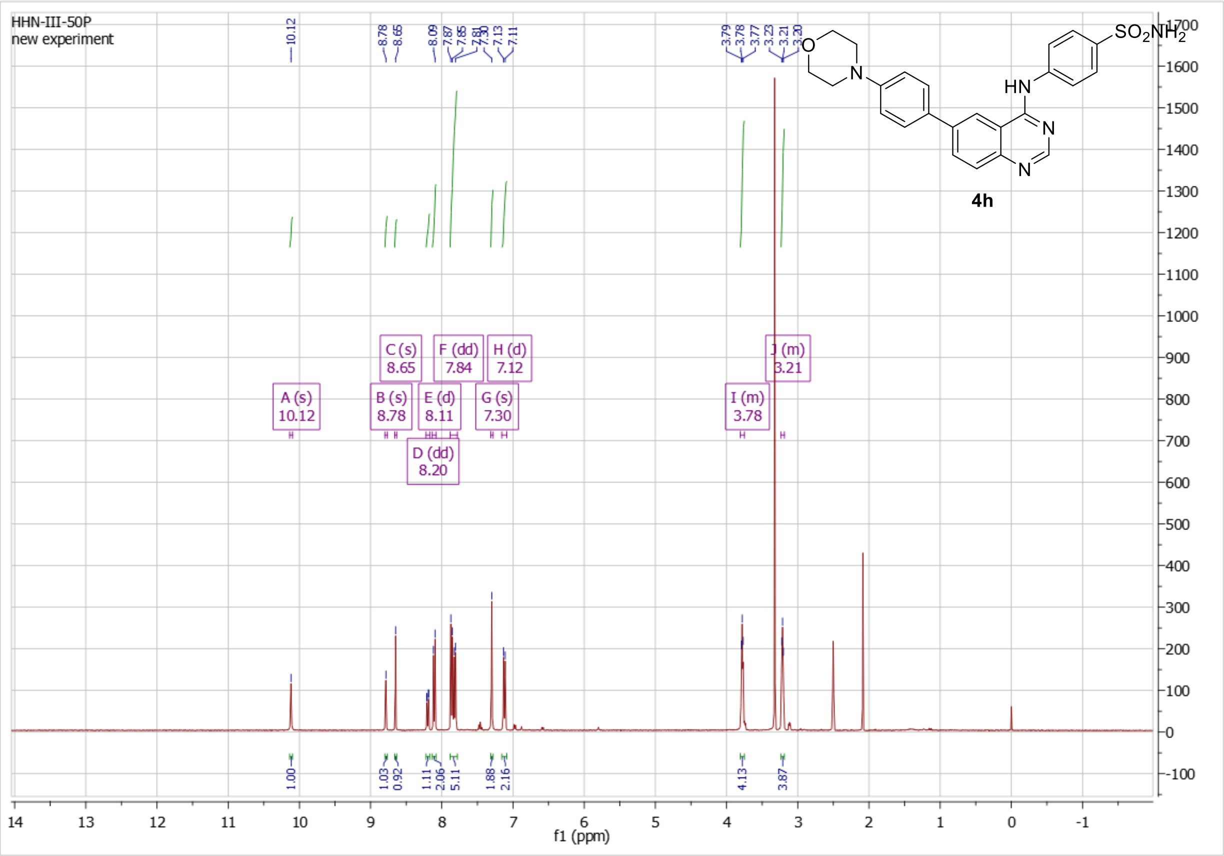

HHN-III-50P
new experiment
A (s) 10.12
B (s) 8.78
C (s) 8.65
D (dd) 8.20
E (d) 8.11
F (dd) 7.84
G (s) 7.30
H (d) 7.12
I (m) 3.78
J (m) 3.21
f1 (ppm)
4h

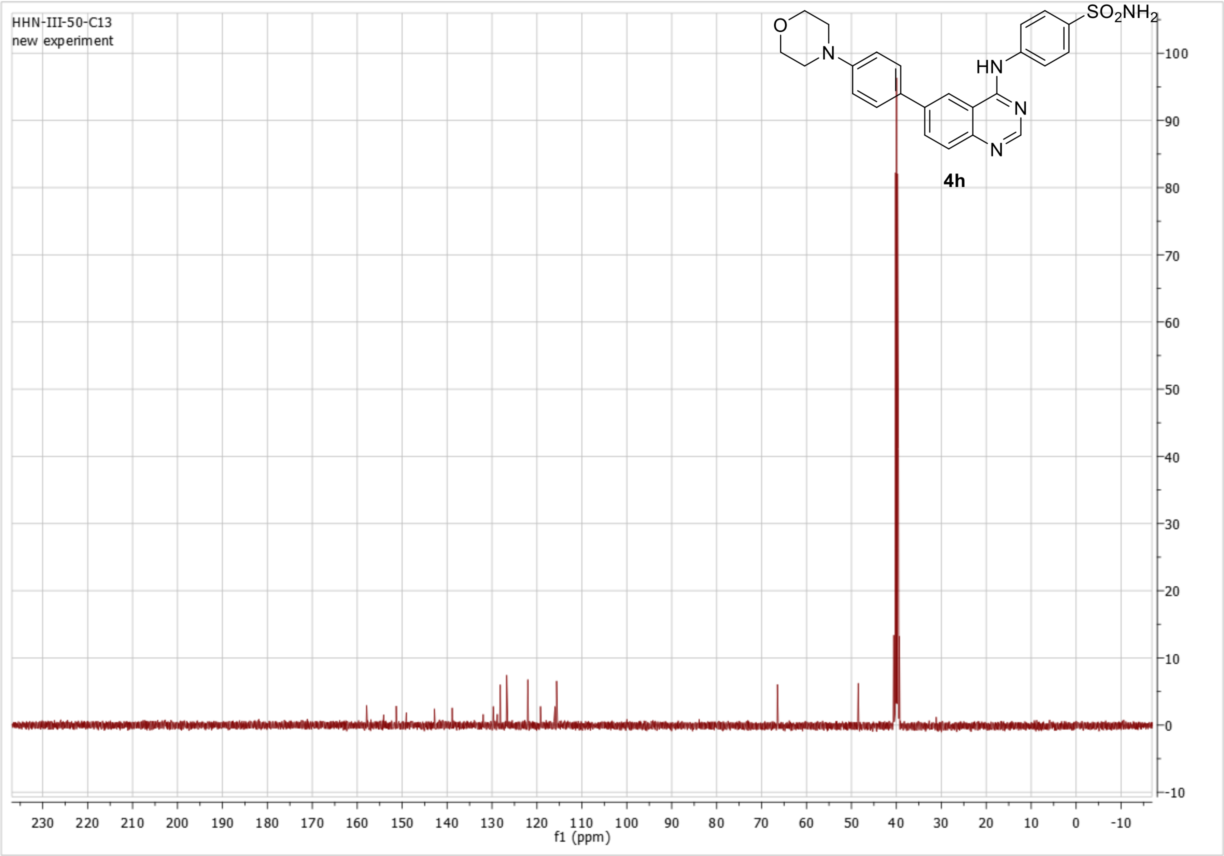
HHN-III-50-C13
new experiment
SO2NH2
HN
N
N
O
N
4h
f1 (ppm)

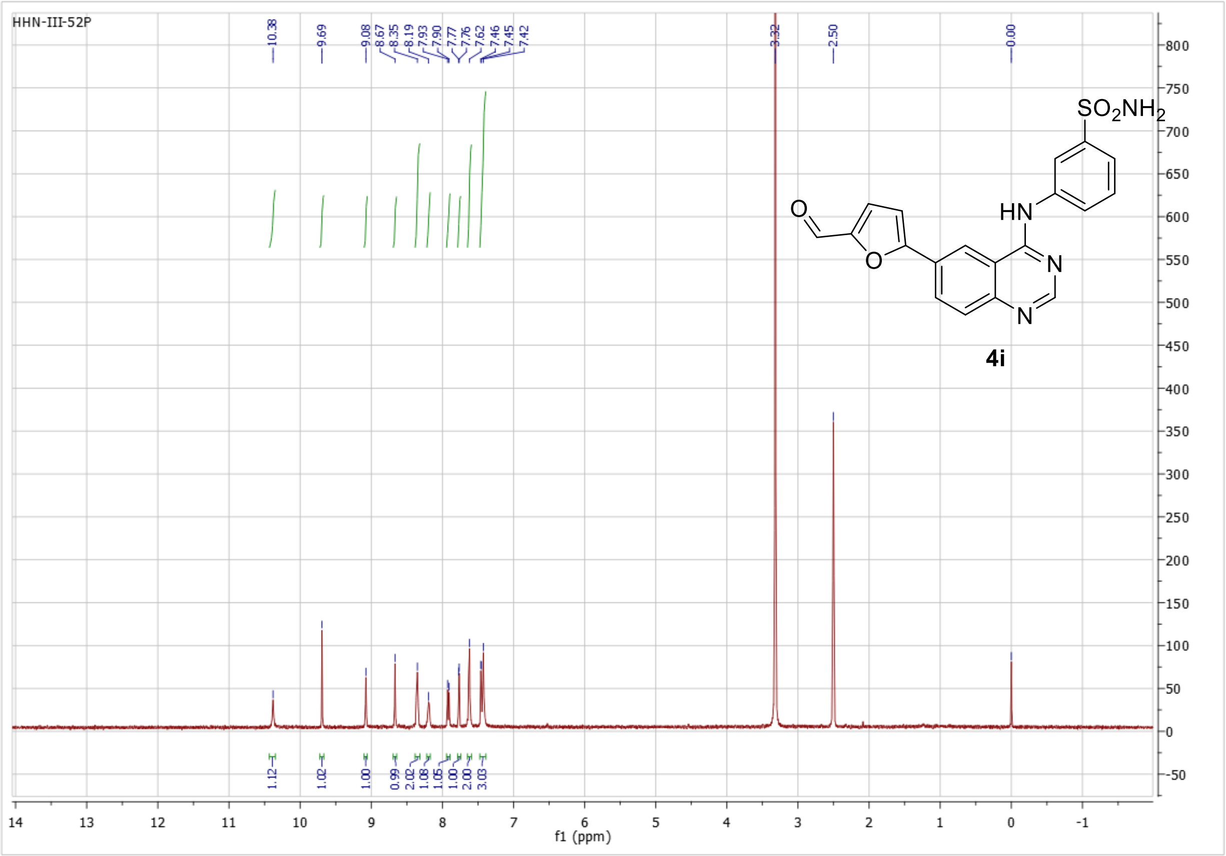
HHN-III-52P
10.38
9.69
9.08
8.67
8.35
8.19
7.93
7.90
7.77
7.76
7.62
7.46
7.45
7.42
3.32
2.50
0.00
1.12
1.02
1.00
0.99
2.02
1.08
1.05
1.00
2.00
3.03
SO2NH2
HN
O
O
N
N
4i
f1 (ppm)

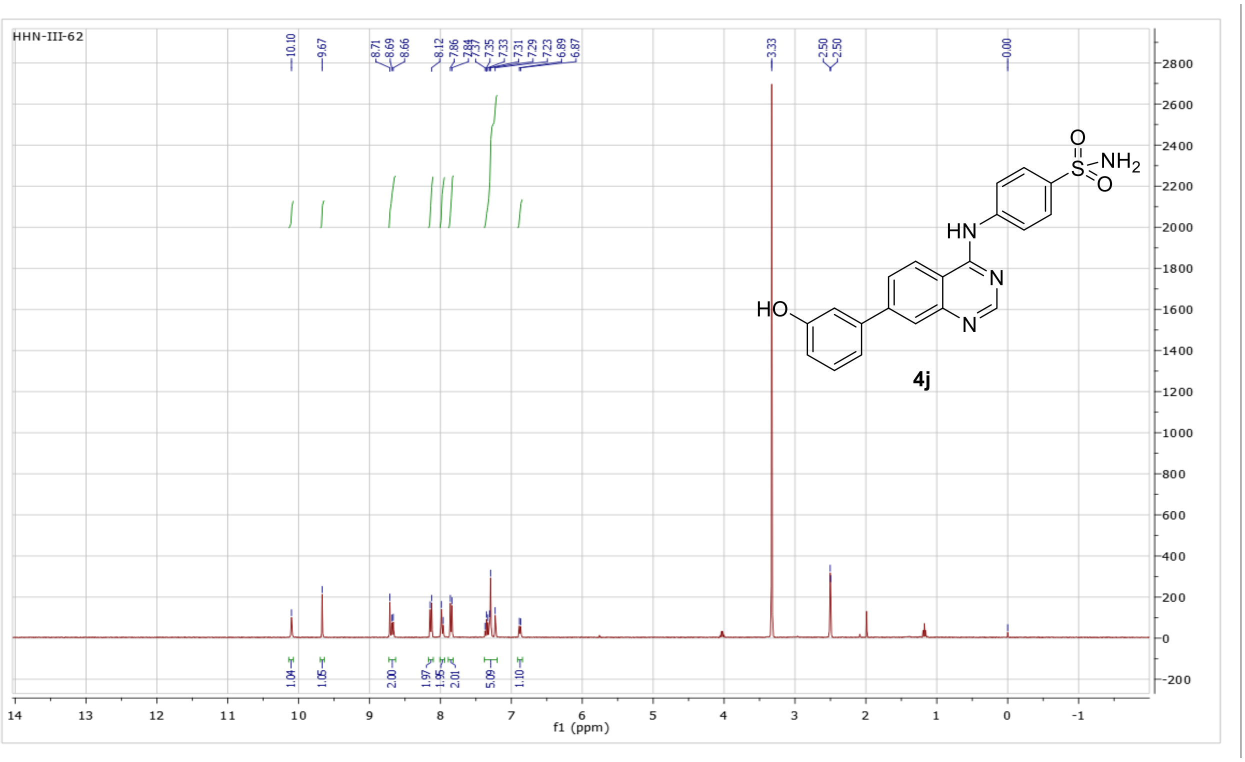
HHN-III-62
10.10
9.67
8.71
8.69
8.66
8.12
7.86
7.35
7.33
7.31
7.29
7.23
6.89
6.87
3.33
2.50
2.50
0.00
1.04
1.05
2.00
1.97
1.95
2.01
5.09
1.10
O
S
NH2
O
HN
N
HO
N
4j
f1 (ppm)

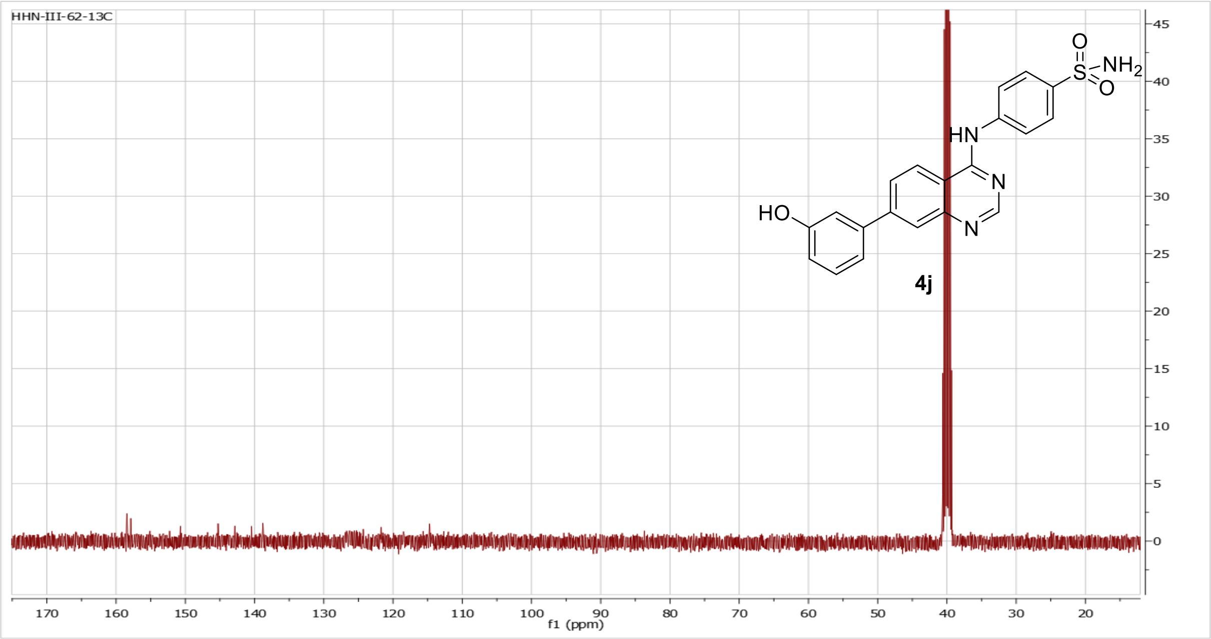
HHN-III-62-13C
f1 (ppm)
4j
HO
HN
N
S
O
NH2

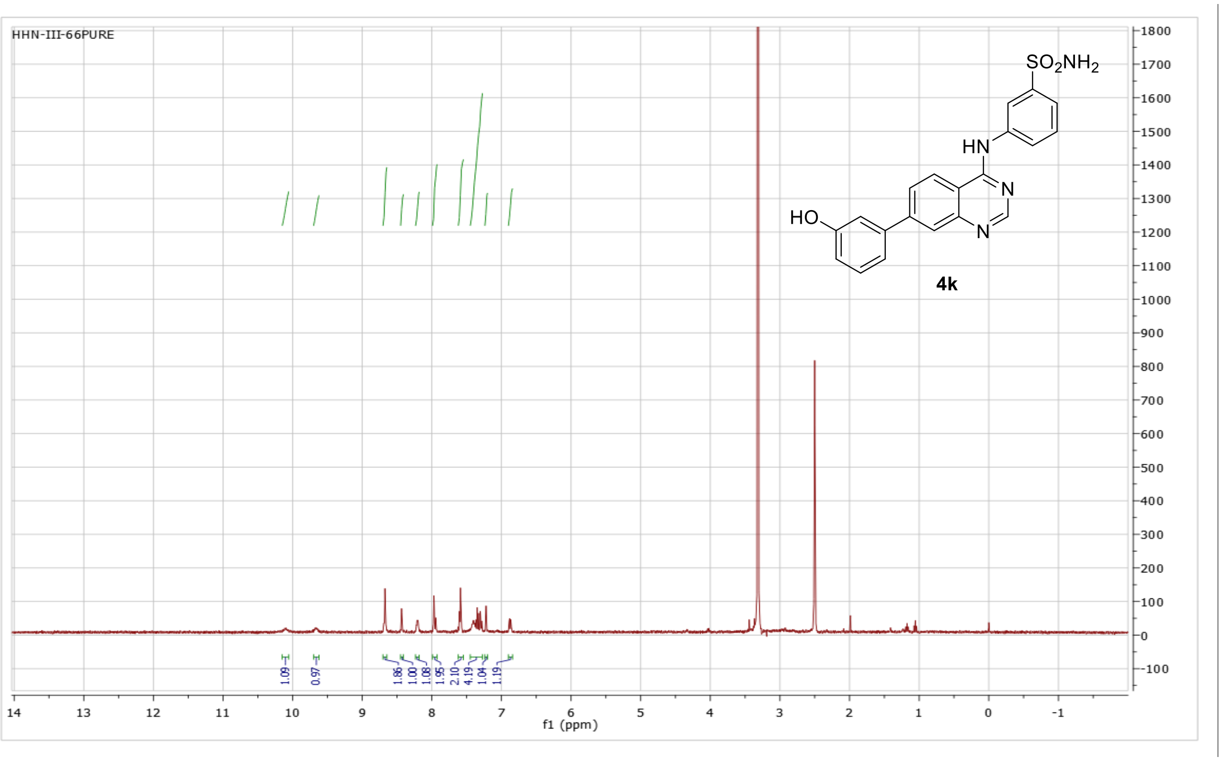
HHN-III-66PURE
f1 (ppm)
4k
HO
HN
N
SO2NH2
1.09
0.97
1.86
1.00
1.08
1.95
2.10
4.19
1.04
1.19

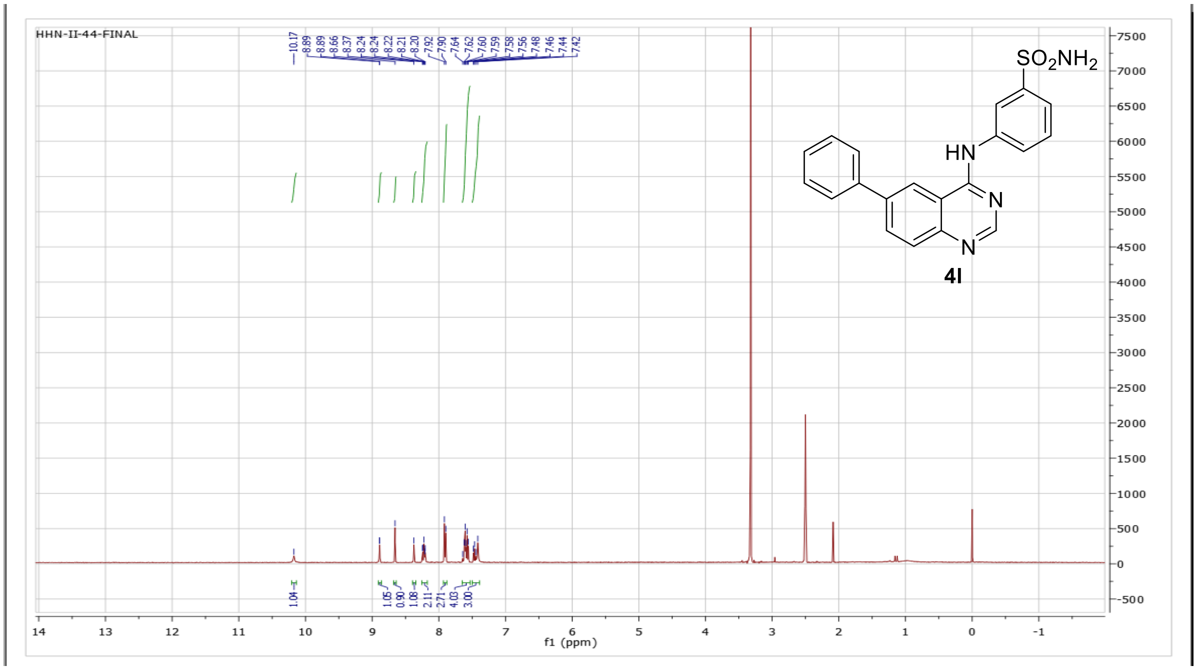

HHN-II-44-FINAL
10.17
8.89
8.89
8.66
8.37
8.24
8.24
8.22
8.21
8.20
7.92
7.90
7.64
7.62
7.60
7.59
7.58
7.56
7.48
7.46
7.44
7.42
SO2NH2
HN
N
N
4l
1.04
1.05
0.90
1.08
2.11
2.71
4.03
3.00
f1 (ppm)

2. Representative HPLC purity data of the compounds 3a–d and 4a–f

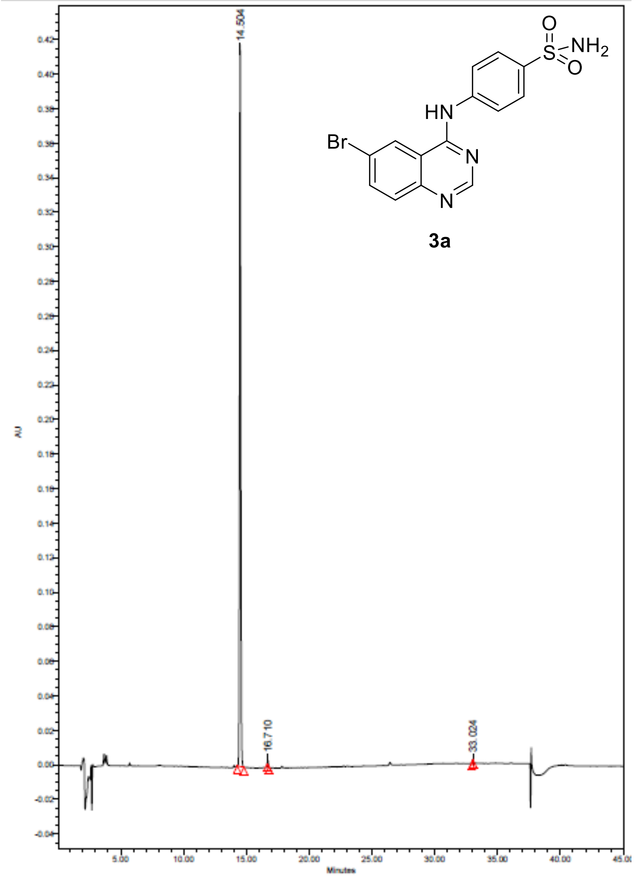

	Name	Retention Time (min)	Purity1 Angle	Purity1 Threshold	PDA Match1 Spect. Name	PDA Match1 Angle	PDA Match1 Threshold	PDA Match1 Lib. Name	Area (µV*sec)	% Area	Height (µV)	Int Type	Amount	Units
1		14.504							2814904	99.46	418996	bb		
2		16.710							11749	0.42	2587	bb		
3		33.024							3664	0.13	851	bb		

	Peak Type	Peak Codes
1	Unknown	
2	Unknown	
3	Unknown	

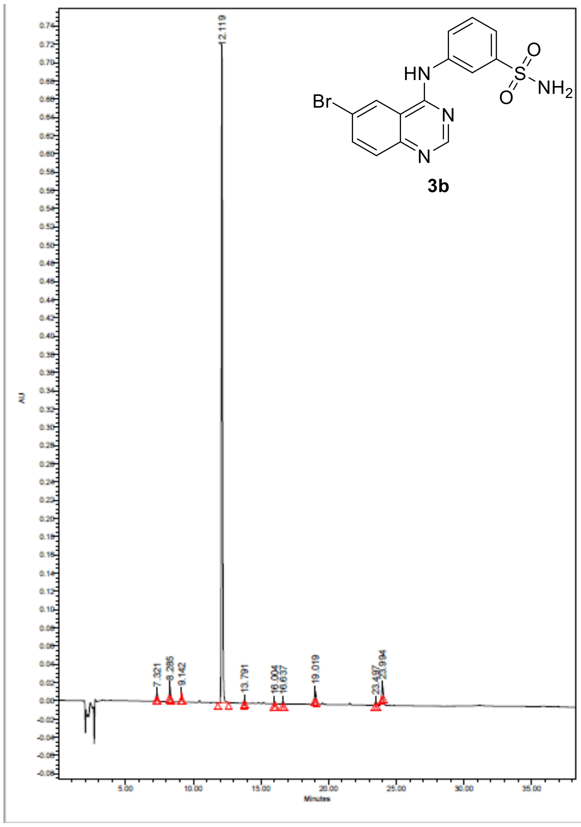

	Name	Retention Time (min)	Purity1 Angle	Purity1 Threshold	PDA Match1 Spect. Name	PDA Match1 Angle	PDA Match1 Threshold	PDA Match1 Lib. Name	Area (µV*sec)	% Area	Height (µV)	Int Type	Amount	Units
1		7.321							19355	0.44	4478	bb		
2		8.285							40947	0.93	9778	bb		
3		9.142							12329	0.28	3927	bb		
4		12.119							4200300	95.11	719106	bb		
5		13.791							4753	0.11	1532	bb		
6		16.004							7963	0.18	1941	bb		
7		16.637							9446	0.21	1950	bb		
8		19.019							46554	1.05	8646	bb		
9		23.497							13240	0.30	2192	bb		
10		23.994							61570	1.39	11239	bb		

	Peak Type	Peak Codes
1	Unknown	
2	Unknown	
3	Unknown	
4	Unknown	
5	Unknown	
6	Unknown	
7	Unknown	

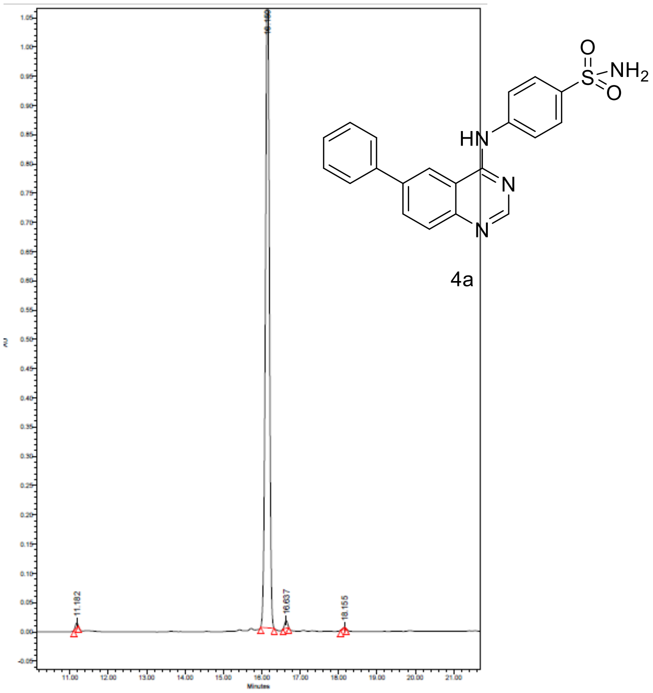

	Name	Retention Time (min)	Purity1 Angle	Purity1 Threshold	PDA Match1 Spect. Name	PDA Match1 Angle	PDA Match1 Threshold	PDA Match1 Lib. Name	Area (µV*sec)	% Area	Height (µV)	Int Type	Amount	Units
1		11.182							32878	0.41	8074	bb		
2		16.150							7916050	98.58	1157431	bb		
3		16.637							64760	0.81	12718	bb		
4		18.155							16762	0.21	3210	bb		

	Peak Type	Peak Codes
1	Unknown	
2	Unknown	
3	Unknown	
4	Unknown	

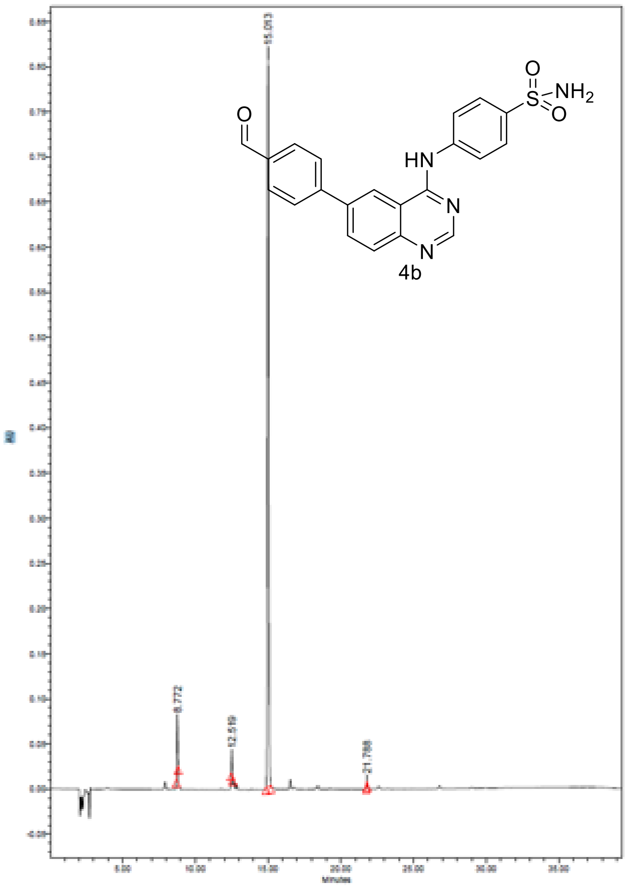

	Name	Retention Time (min)	Purity1 Angle	Purity1 Threshold	PDA Match1 Spect. Name	PDA Match1 Angle	PDA Match1 Threshold	PDA Match1 Lib. Name	Area (µV*sec)	% Area	Height (µV)	Int Type	Amount	Units
1		8.772							227402	4.30	57247	bb		
2		12.519							76296	1.44	21075	bb		
3		15.013							4983721	94.26	818680	bb		

	Peak Type	Peak Codes
1	Unknown	
2	Unknown	
3	Unknown	

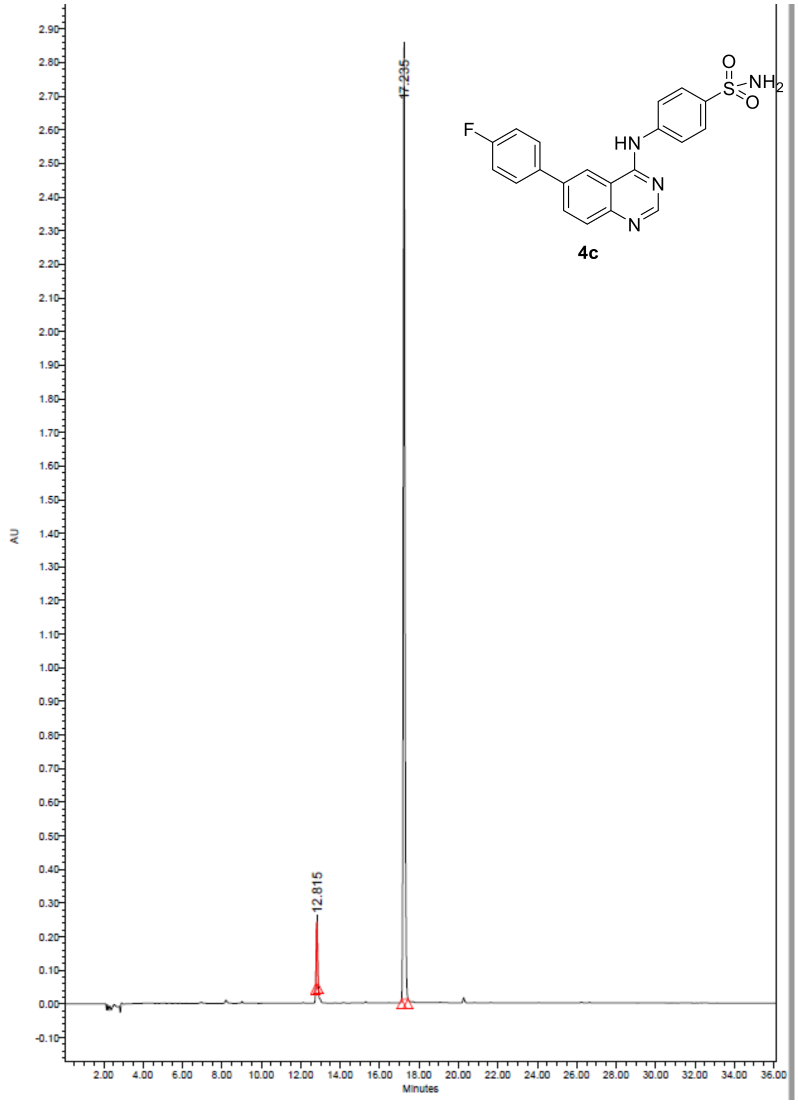

	Name	Retention Time (min)	Purity1 Angle	Purity1 Threshold	PDA Match1 Spect. Name	PDA Match1 Angle	PDA Match1 Threshold	PDA Match1 Lib. Name	Area (µV*sec)	% Area	Height (µV)	Int Type	Amount	Units
1		12.815							830104	4.81	185459	bb		
2		17.235							16416914	95.19	2801847	bb		

	Peak Type	Peak Codes
1	Unknown	
2	Unknown	

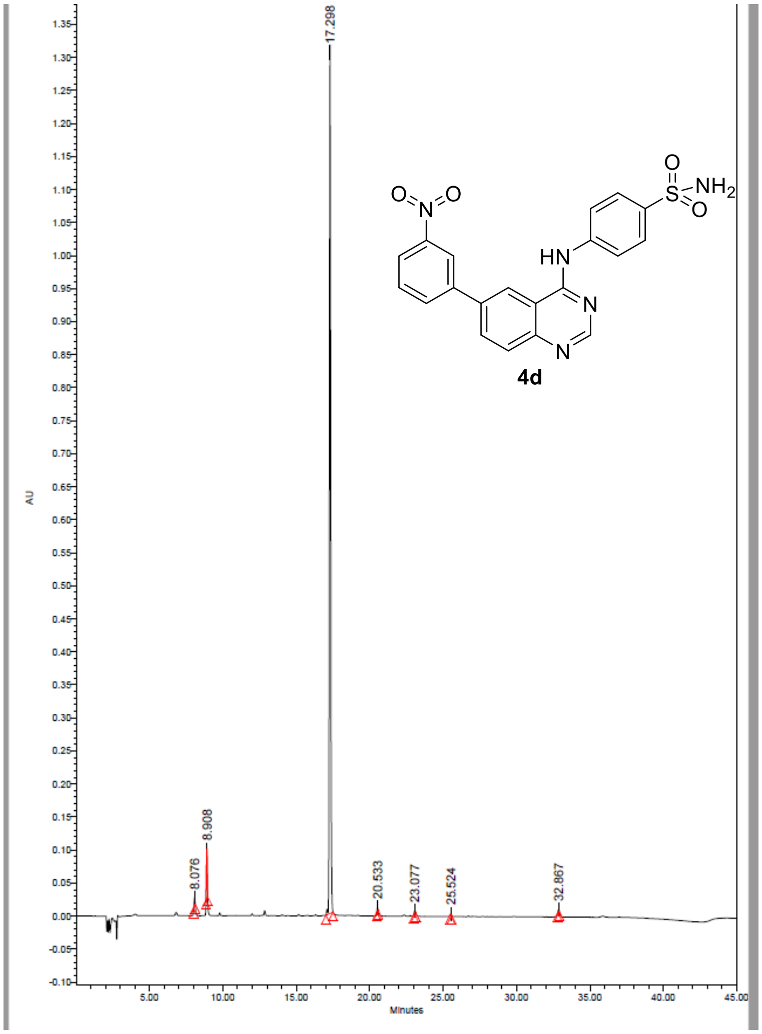

	Name	Retention Time (min)	Purity1 Angle	Purity1 Threshold	PDA Match1 Spect. Name	PDA Match1 Angle	PDA Match1 Threshold	PDA Match1 Lib. Name	Area (µV*sec)	% Area	Height (µV)	Int Type	Amount	Units
1		8.076							47044	0.57	12618	bb		
2		8.908							302062	3.68	75700	bb		
3		17.298							7806171	95.08	1311111	bb		
4		20.533							17098	0.21	5429	bb		
5		23.077							16161	0.20	3734	bb		
6		25.524							5168	0.06	1350	bb		
7		32.867							16824	0.20	3804	bb		

	Peak Type	Peak Codes
1	Unknown	
2	Unknown	
3	Unknown	
4	Unknown	
5	Unknown	
6	Unknown	
7	Unknown	

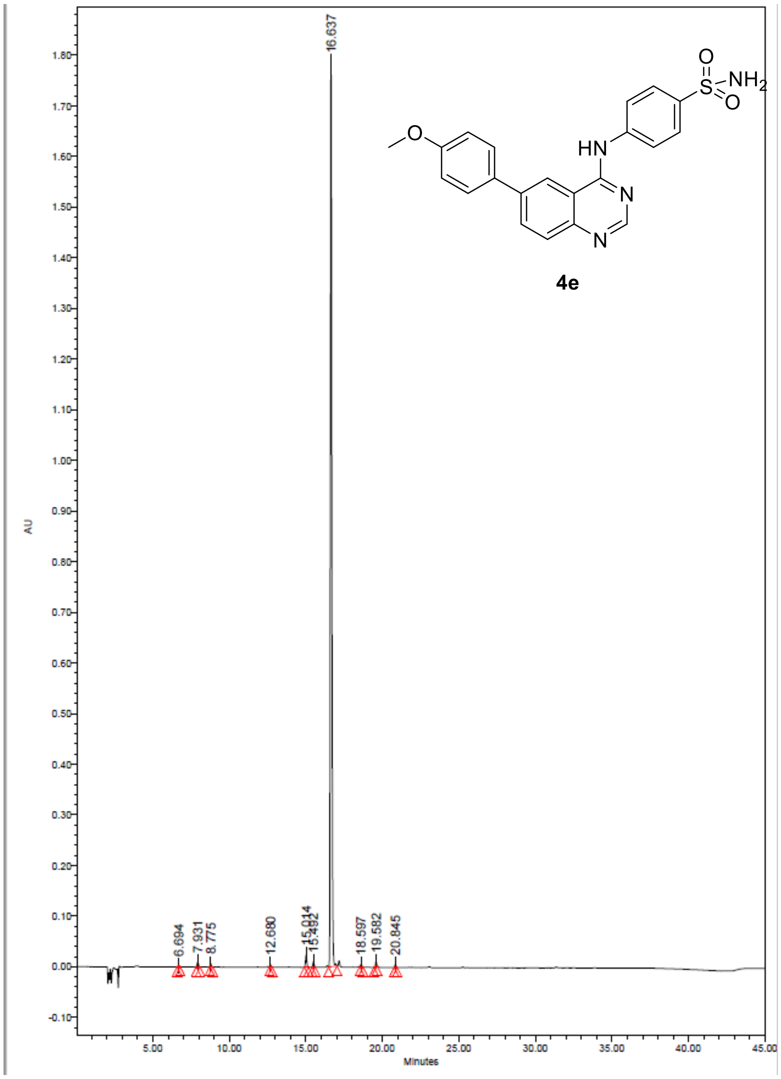

	Name	Retention Time (min)	Purity1 Angle	Purity1 Threshold	PDA Match1 Spect. Name	PDA Match1 Angle	PDA Match1 Threshold	PDA Match1 Lib. Name	Area (μV*sec)	% Area	Height (μV)	Int Type	Amount	Units
1		6.694							14357	0.12	2244	bb		
2		7.931							58103	0.47	9352	bb		
3		8.775							26627	0.21	5630	bb		
4		12.680							20602	0.17	4220	bb		
5		15.014							144110	1.16	24188	bb		
6		15.492							61469	0.49	11138	bb		
7		16.637							12017479	96.57	1812810	bb		
8		18.597							27416	0.22	4151	bb		
9		19.582							44484	0.36	9451	bb		
10		20.845							30110	0.24	5670	bb		

	Peak Type	Peak Codes
1	Unknown	
2	Unknown	
3	Unknown	
4	Unknown	
5	Unknown	
6	Unknown	
7	Unknown	

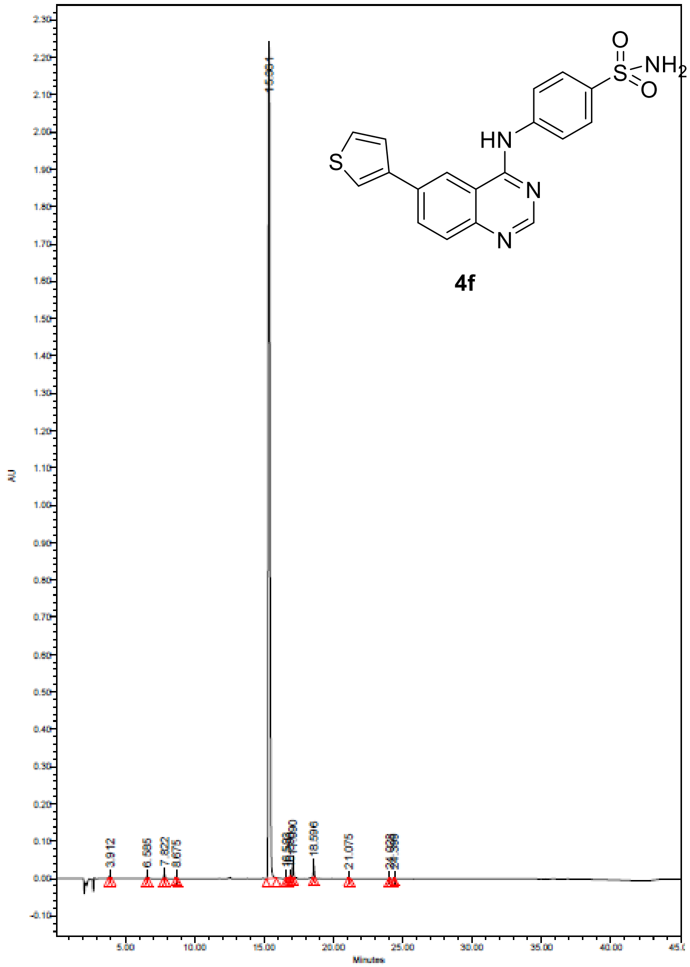

	Name	Retention Time (min)	Purity1 Angle	Purity1 Threshold	PDA Match1 Spect. Name	PDA Match1 Angle	PDA Match1 Threshold	PDA Match1 Lib. Name	Area (µV*sec)	% Area	Height (µV)	Int Type	Amount	Units
1		3.912							38329	0.19	4362	bb		
2		6.585							39898	0.19	5960	bb		
3		7.822							74062	0.36	11982	bb		
4		8.675							9762	0.05	2722	bb		
5		15.361							19995834	97.18	2252689	bb		
6		16.593							17410	0.08	4021	bb		
7		16.890							21119	0.10	5240	bb		
8		17.090							198514	0.96	37851	bb		
9		18.596							160684	0.78	30903	bb		
10		21.075							8154	0.04	1953	bb		
11		24.028							9243	0.04	1986	bb		
12		24.399							3054	0.01	1144	bb		

	Peak Type	Peak Codes
1	Unknown	
2	Unknown	
3	Unknown	
4	Unknown	
5	Unknown	

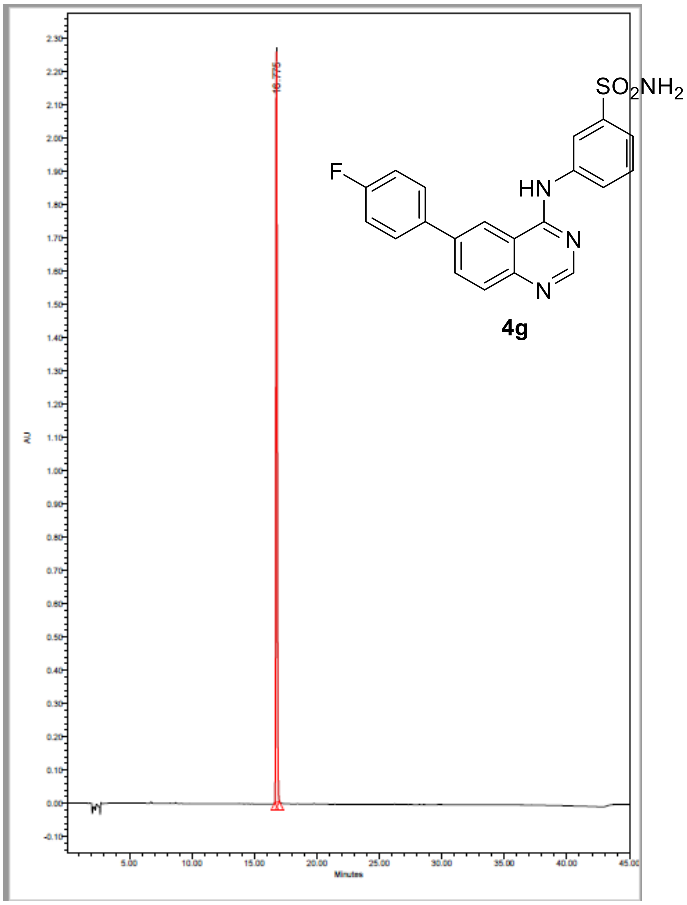

	Name	Retention Time (min)	Purity1 Angle	Purity1 Threshold	PDA Match1 Spect. Name	PDA Match1 Angle	PDA Match1 Threshold	PDA Match1 Lib. Name	Area (µV*sec)	% Area	Height (µV)	Int Type	Amount	Units
1		16.775							16022791	100.00	2262610	bb		

	Peak Type	Peak Codes
1	Unknown	

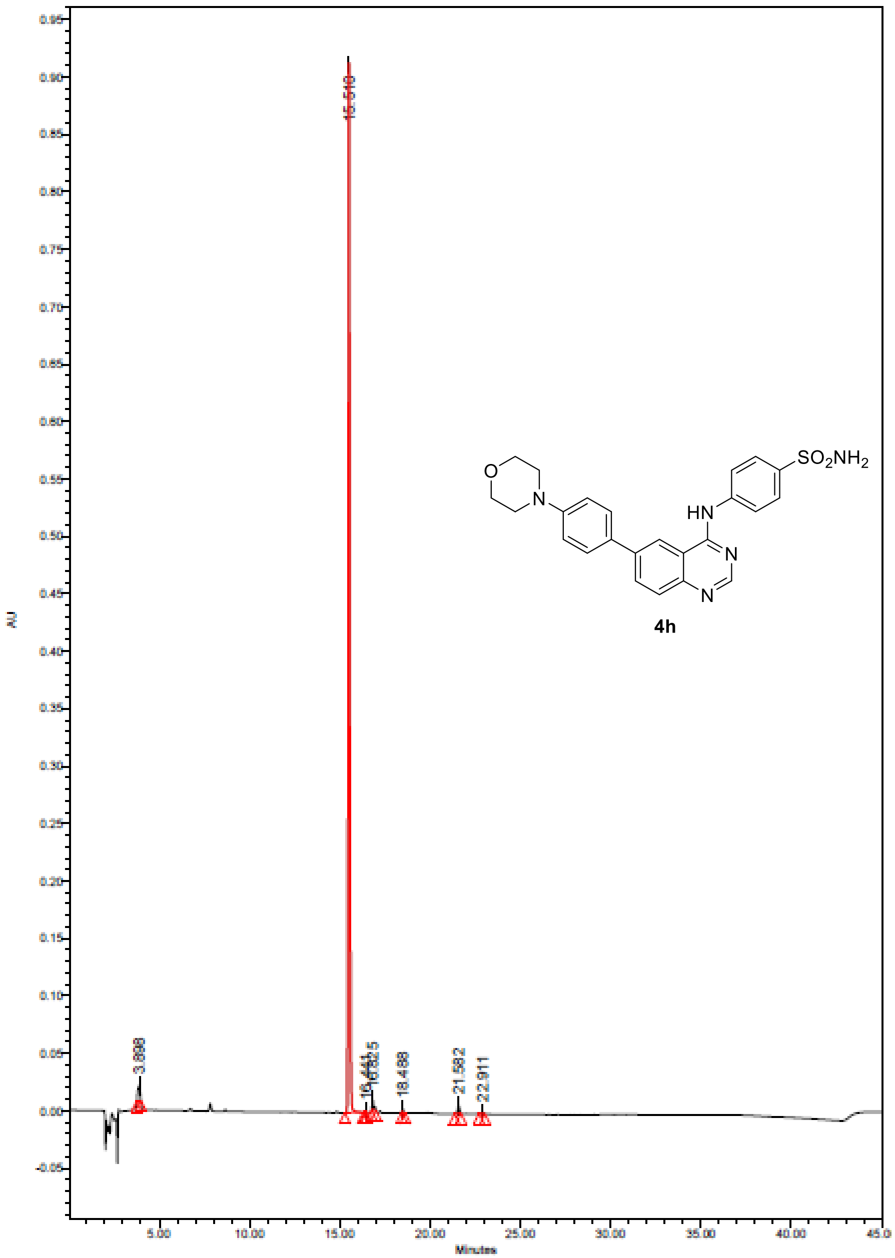

	Name	Retention Time (min)	Purity1 Angle	Purity1 Threshold	PDA Match1 Spect. Name	PDA Match1 Angle	PDA Match1 Threshold	PDA Match1 Lib. Name	Area (µV*sec)	% Area	Height (µV)	Int Type	Amount	Units
1		3.898							122192	2.23	13150	bb		
2		15.510							5214400	95.27	905048	bb		
3		16.441							3167	0.06	867	bb		
4		16.825							54410	0.99	8926	bb		
5		18.488							14350	0.26	2914	bb		
6		21.582							52919	0.97	6797	bb		
7		22.911							11878	0.22	1330	bb		

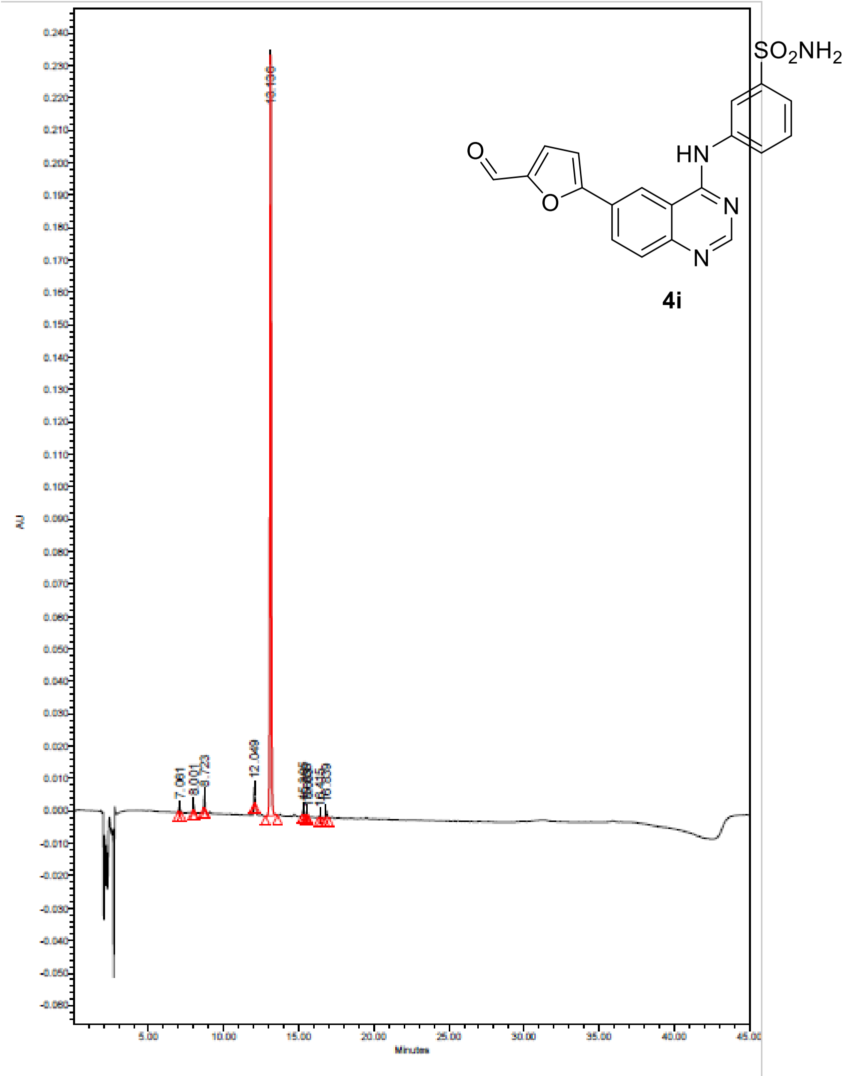

	Name	Retention Time (min)	Purity1 Angle	Purity1 Threshold	PDA Match1 Spect. Name	PDA Match1 Angle	PDA Match1 Threshold	PDA Match1 Lib. Name	Area (µV*sec)	% Area	Height (µV)	Int Type	Amount	Units
1		7.061							10398	0.74	1388	bb		
2		8.001							6112	0.44	1742	bb		
3		8.723							16055	1.15	4045	bb		
4		12.049							17560	1.26	5014	bb		
5		13.136							1329898	95.09	232034	bb		
6		15.305							6206	0.44	1452	bb		
7		15.538							4342	0.31	1215	bb		
8		15.603							0	0.00	0	tt		
9		16.415							1704	0.12	343	bb		
10		16.839							6239	0.45	1554	bb		

	Peak Type	Peak Codes
1	Unknown	
2	Unknown	
3	Unknown	
4	Unknown	
5	Unknown	
6	Unknown	
7	Unknown	
8	Unknown	I05
9	Unknown	
10	Unknown	

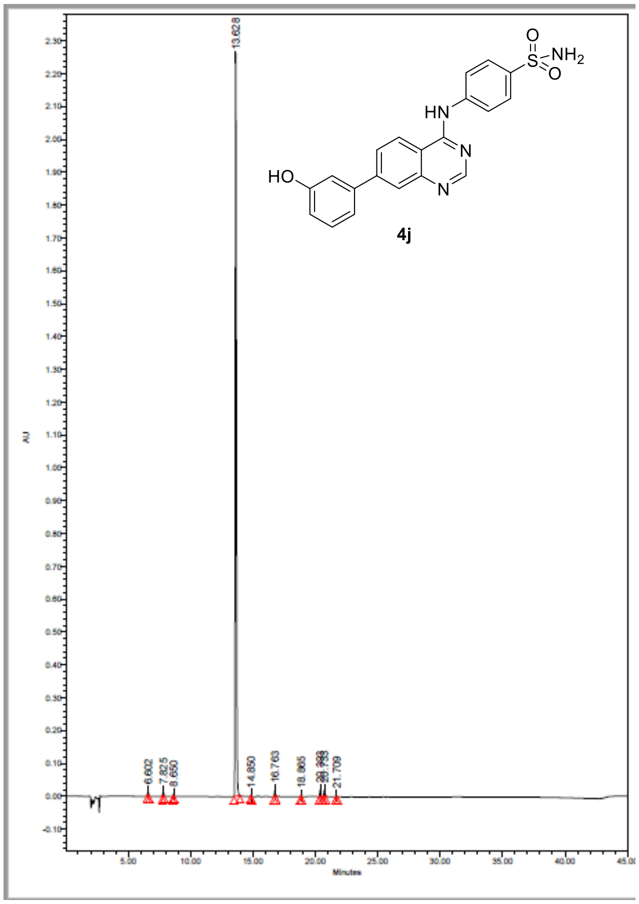

	Name	Retention Time (min)	Purity1 Angle	Purity1 Threshold	PDA Match1 Spect. Name	PDA Match1 Angle	PDA Match1 Threshold	PDA Match1 Lib. Name	Area (µV*sec)	% Area	Height (µV)	Int Type	Amount	Units
1		6.602							61572	0.40	10046	bb		
2		7.825							61218	0.39	11418	bb		
3		8.650							24893	0.16	5862	bb		
4		13.628							15014404	96.82	2285484	bb		
5		14.850							6524	0.04	1936	bb		
6		16.763							89713	0.58	17181	bb		
7		18.865							9345	0.06	2133	bb		
8		20.393							107303	0.69	19431	bb		
9		20.733							116863	0.75	19392	bb		
10		21.709							16226	0.10	3145	bb		

	Peak Type	Peak Codes
1	Unknown	
2	Unknown	
3	Unknown	
4	Unknown	
5	Unknown	
6	Unknown	
7	Unknown	
8	Unknown	
9	Unknown	
10	Unknown	

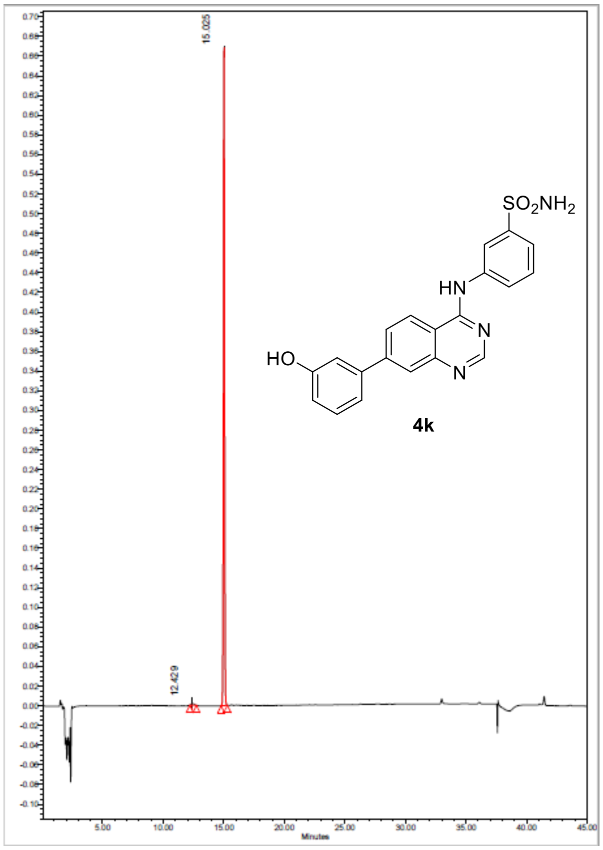

	Name	Retention Time (min)	Purity1 Angle	Purity1 Threshold	PDA Match1 Spect. Name	PDA Match1 Angle	PDA Match1 Threshold	PDA Match1 Lib. Name	Area (μV*sec)	% Area	Height (μV)	Int Type	Amount	Units
1		12.429							30824	0.68	1764	bb		
2		15.025							4493452	99.32	671385	bb		

its	Peak Type	Peak Codes
1	Unknown	
2	Unknown	

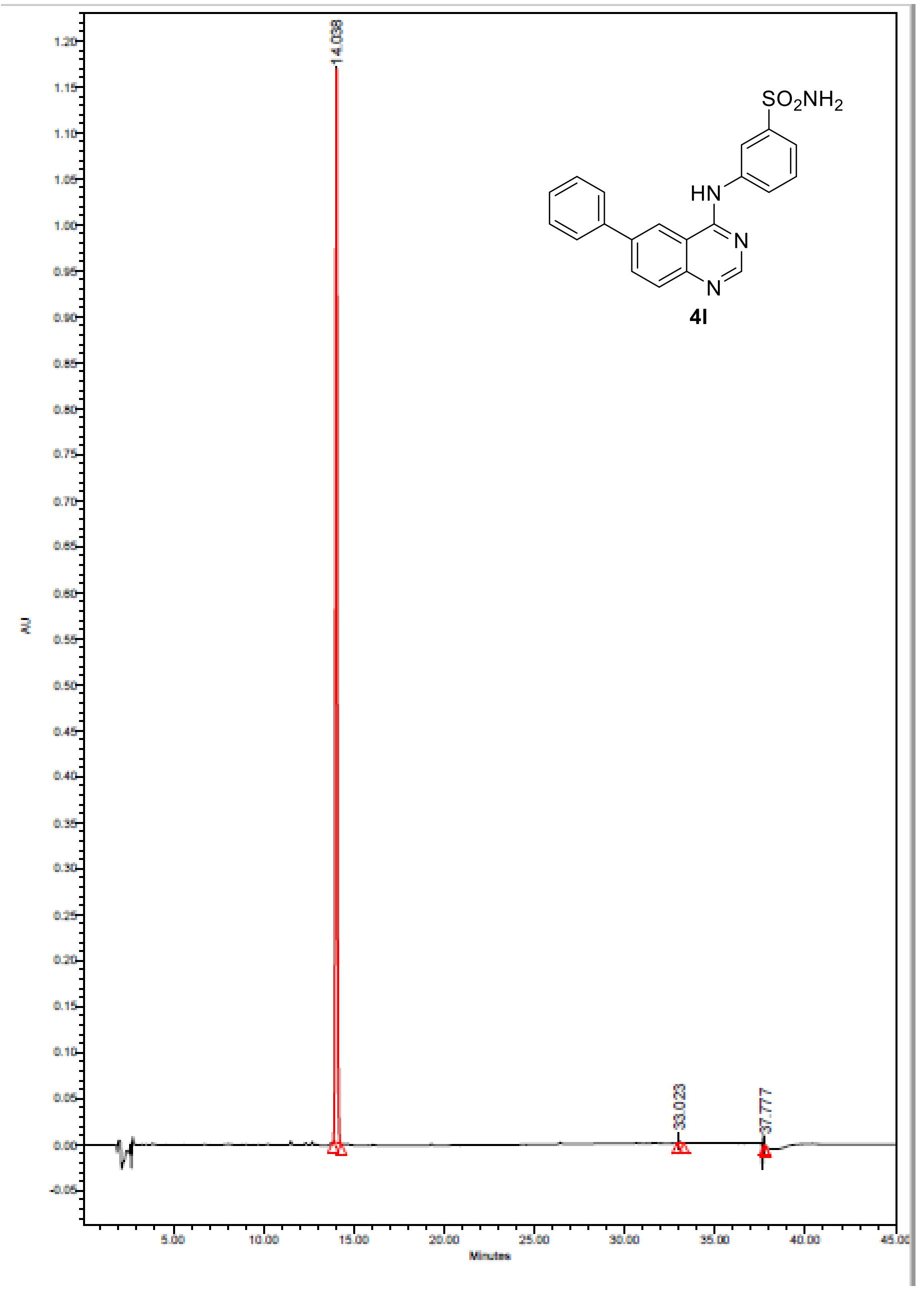

	Name	Retention Time (min)	Purity1 Angle	Purity1 Threshold	PDA Match1 Spect. Name	PDA Match1 Angle	PDA Match1 Threshold	PDA Match1 Lib. Name	Area (µV*sec)	% Area	Height (µV)	Int Type	Amount	Units
1		14.038							7876666	99.81	1168263	bb		
2		33.023							14625	0.19	1987	bb		
3		37.777							563	0.01	-225	bb		

	Peak Type	Peak Codes
1	Unknown	
2	Unknown	
3	Unknown	I08

3. Representative HRMS data of the compounds 3a–d and 4a–f

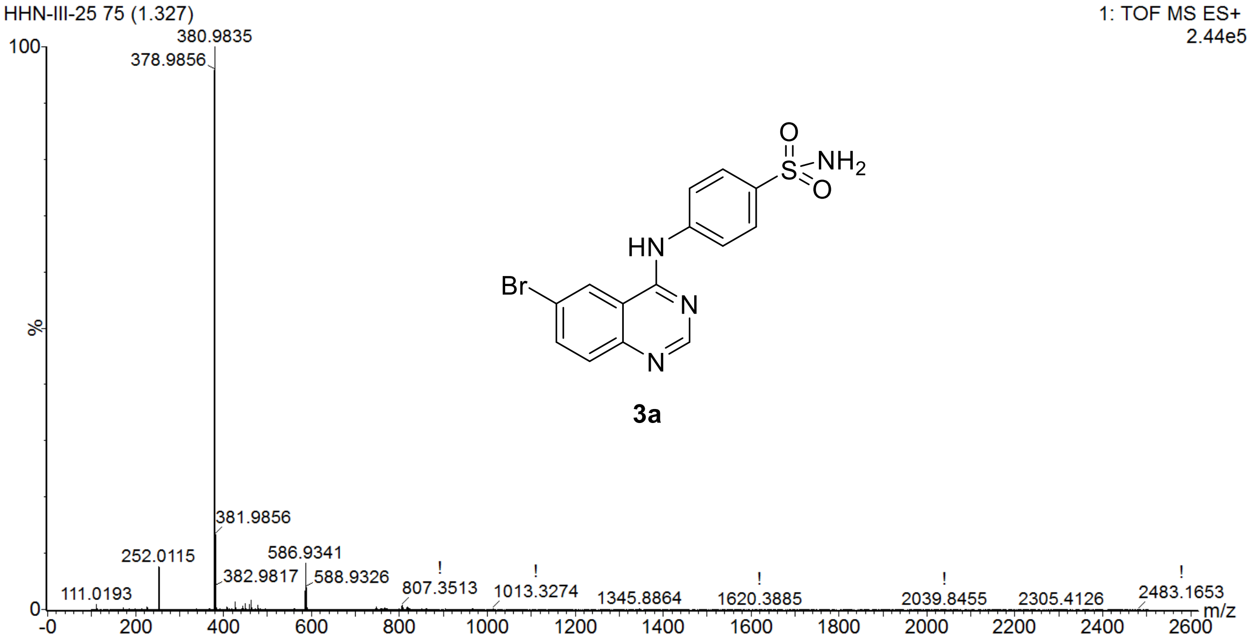

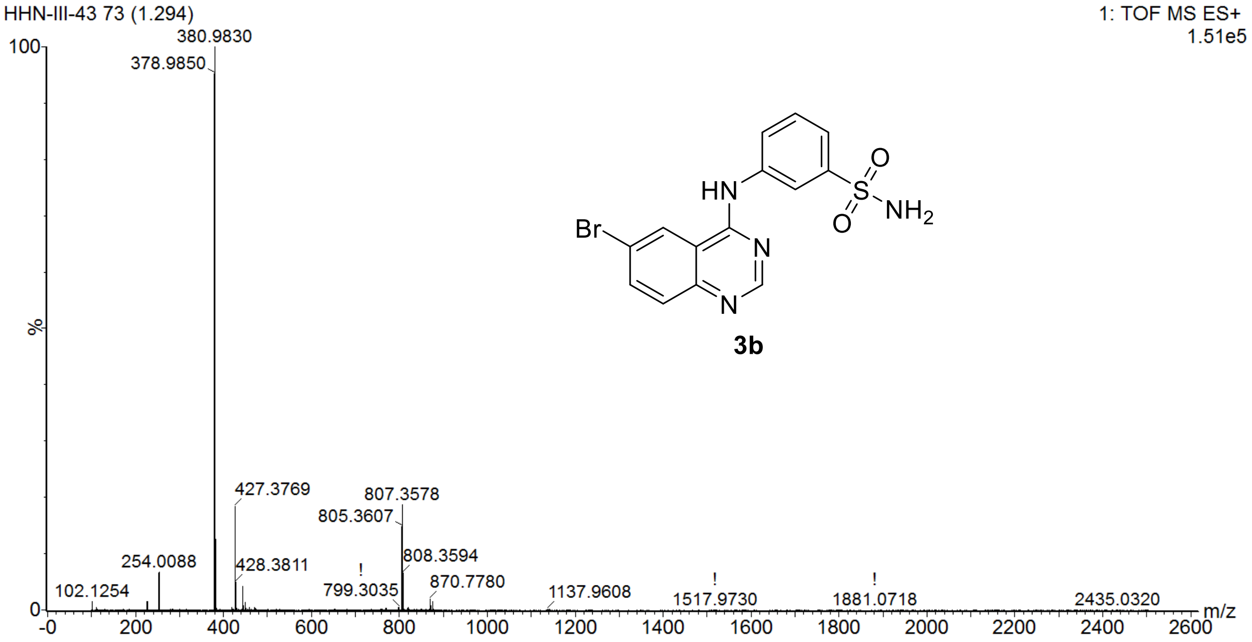

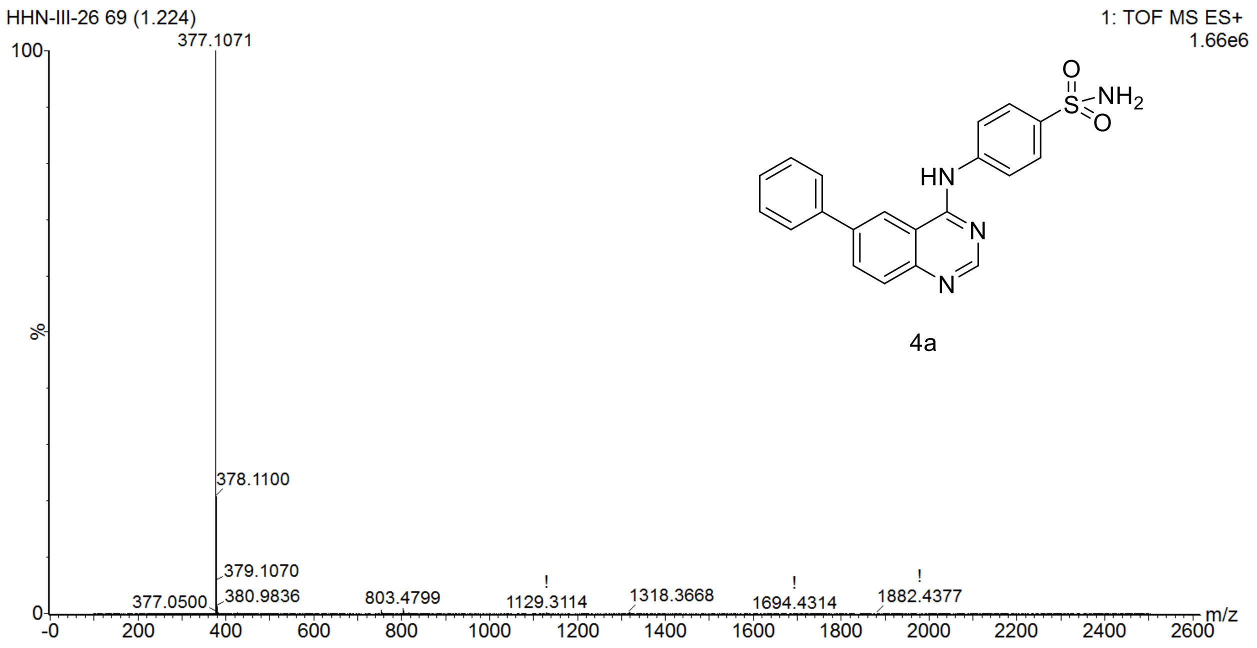
HHN-III-26 69 (1.224)
1: TOF MS ES+
1.66e6
377.1071
378.1100
379.1070
377.0500
380.9836
803.4799
1129.3114
1318.3668
1694.4314
1882.4377
%
m/z
4a

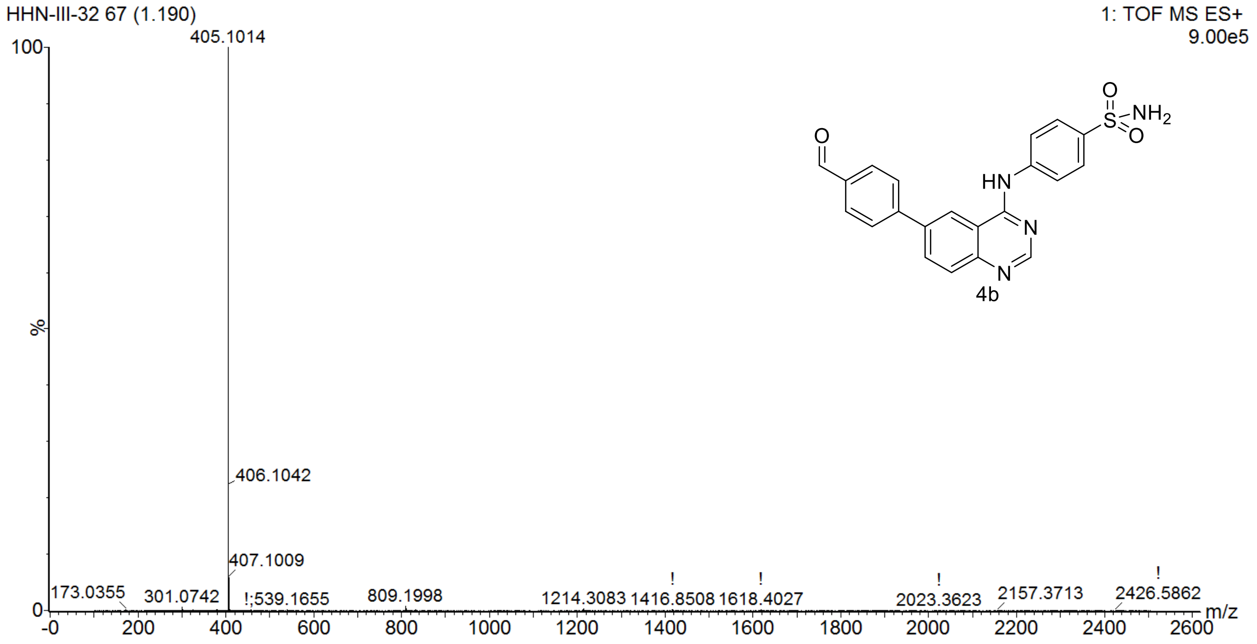
HHN-III-32 67 (1.190)
1: TOF MS ES+
9.00e5
405.1014
406.1042
407.1009
173.0355
301.0742
539.1655
809.1998
1214.3083
1416.8508
1618.4027
2023.3623
2157.3713
2426.5862
%
m/z
4b

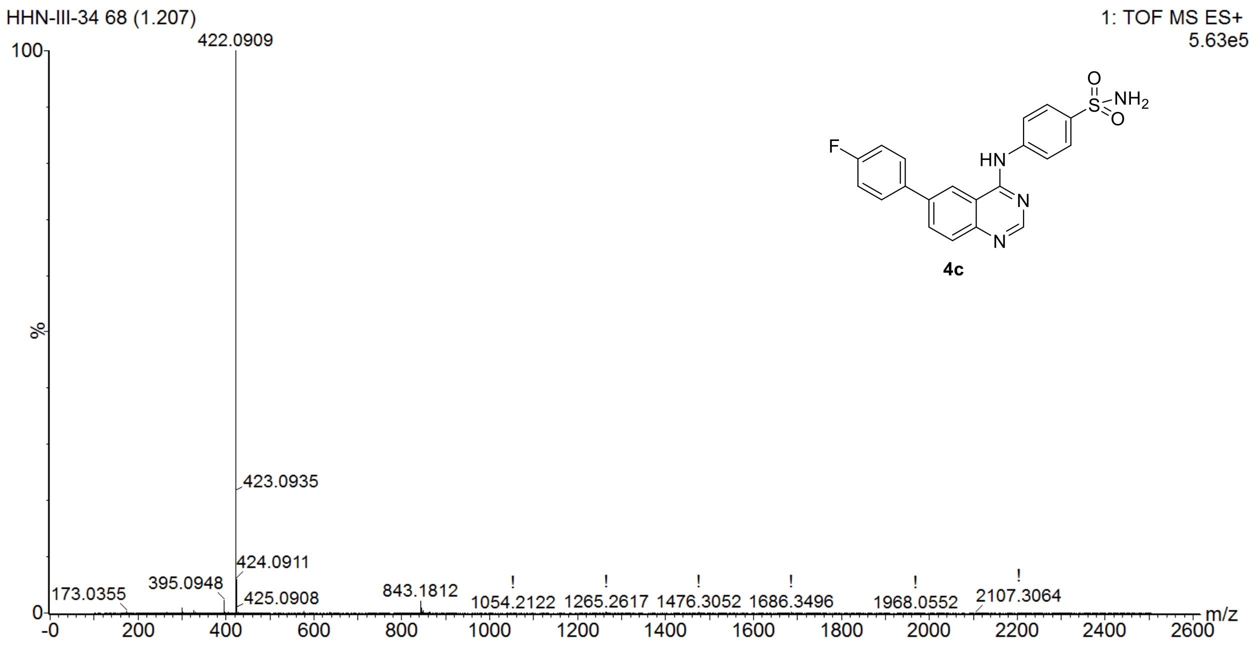
HHN-III-34 68 (1.207)
1: TOF MS ES+
5.63e5
422.0909
423.0935
424.0911
425.0908
395.0948
173.0355
843.1812
1054.2122
1265.2617
1476.3052
1686.3496
1968.0552
2107.3064
%
m/z
4c

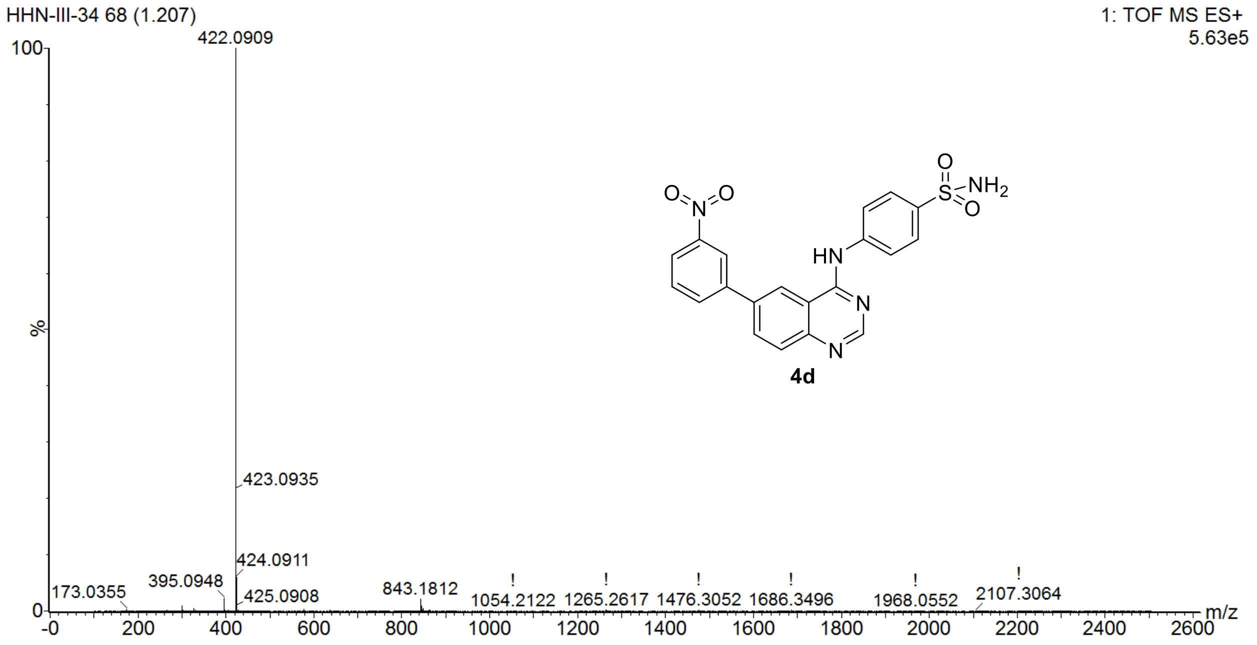
HHN-III-34 68 (1.207)
1: TOF MS ES+
5.63e5
422.0909
423.0935
424.0911
425.0908
395.0948
173.0355
843.1812
1054.2122
1265.2617
1476.3052
1686.3496
1968.0552
2107.3064
%
m/z
4d

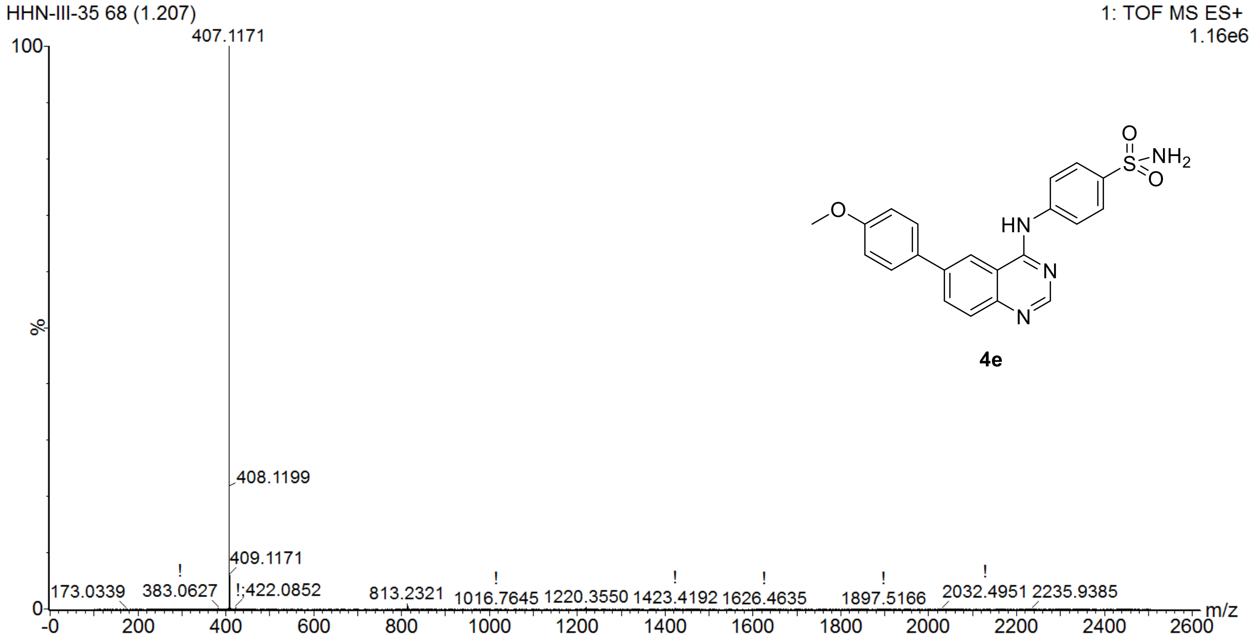
HHN-III-35 68 (1.207)
1: TOF MS ES+
1.16e6
407.1171
408.1199
409.1171
422.0852
173.0339
383.0627
813.2321
1016.7645
1220.3550
1423.4192
1626.4635
1897.5166
2032.4951
2235.9385
%
m/z
4e

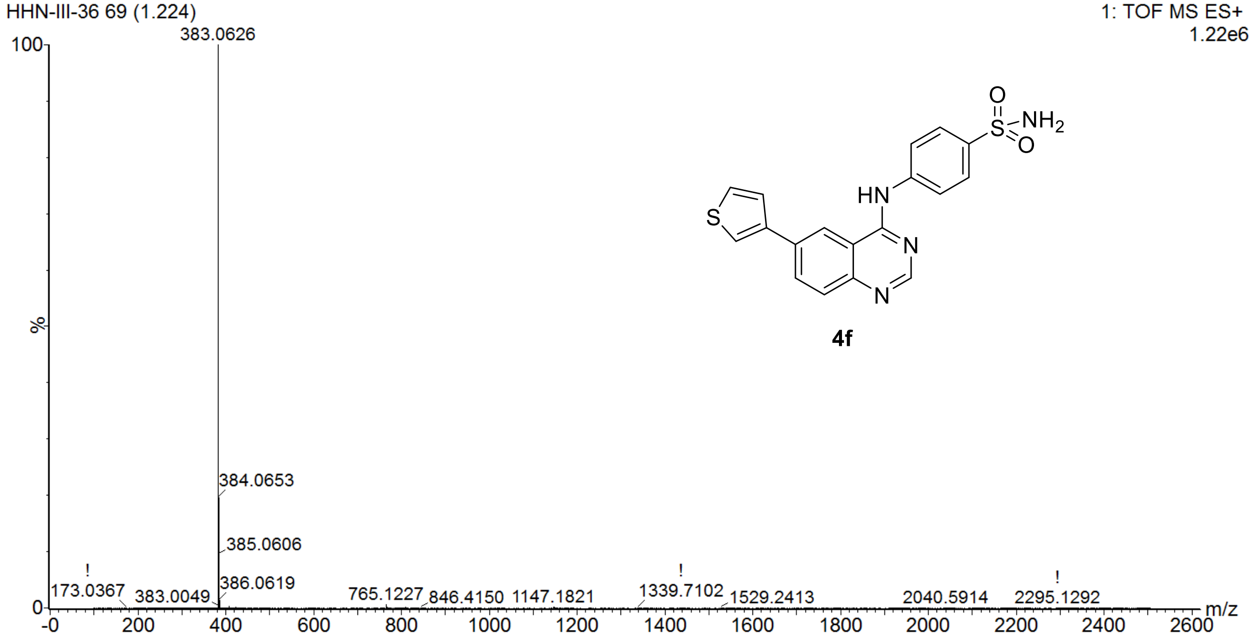
HHN-III-36 69 (1.224)
1: TOF MS ES+
1.22e6
383.0626
384.0653
385.0606
386.0619
173.0367
383.0049
765.1227
846.4150
1147.1821
1339.7102
1529.2413
2040.5914
2295.1292
%
m/z
4f

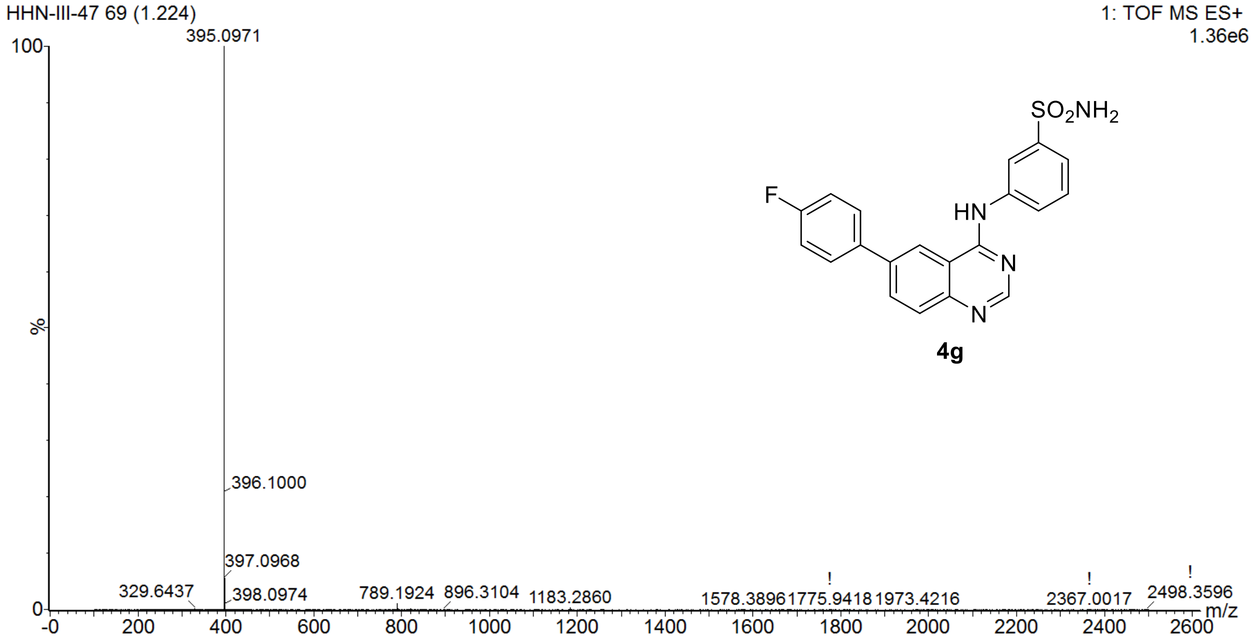
HHN-III-47 69 (1.224)
1: TOF MS ES+
1.36e6
395.0971
396.1000
397.0968
398.0974
329.6437
789.1924
896.3104
1183.2860
1578.3896
1775.9418
1973.4216
2367.0017
2498.3596
%
m/z
SO2NH2
F
HN
N
N
4g

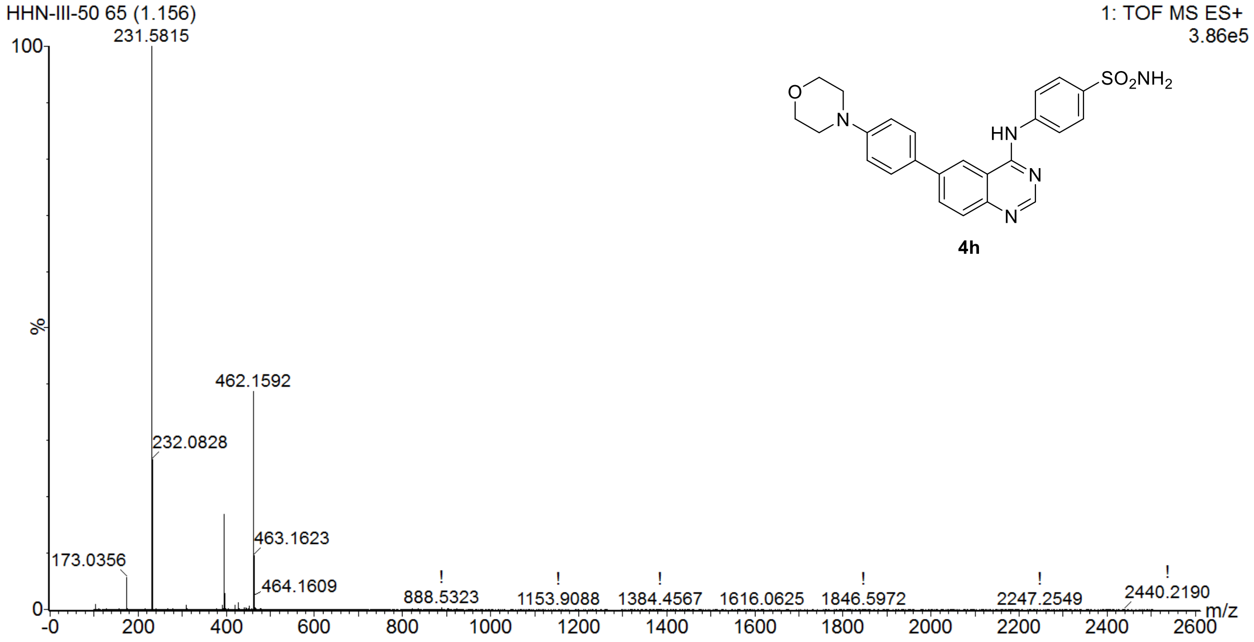
HHN-III-50 65 (1.156)
1: TOF MS ES+
3.86e5
231.5815
462.1592
232.0828
463.1623
173.0356
464.1609
888.5323
1153.9088
1384.4567
1616.0625
1846.5972
2247.2549
2440.2190
%
m/z
SO2NH2
O
N
HN
N
N
4h

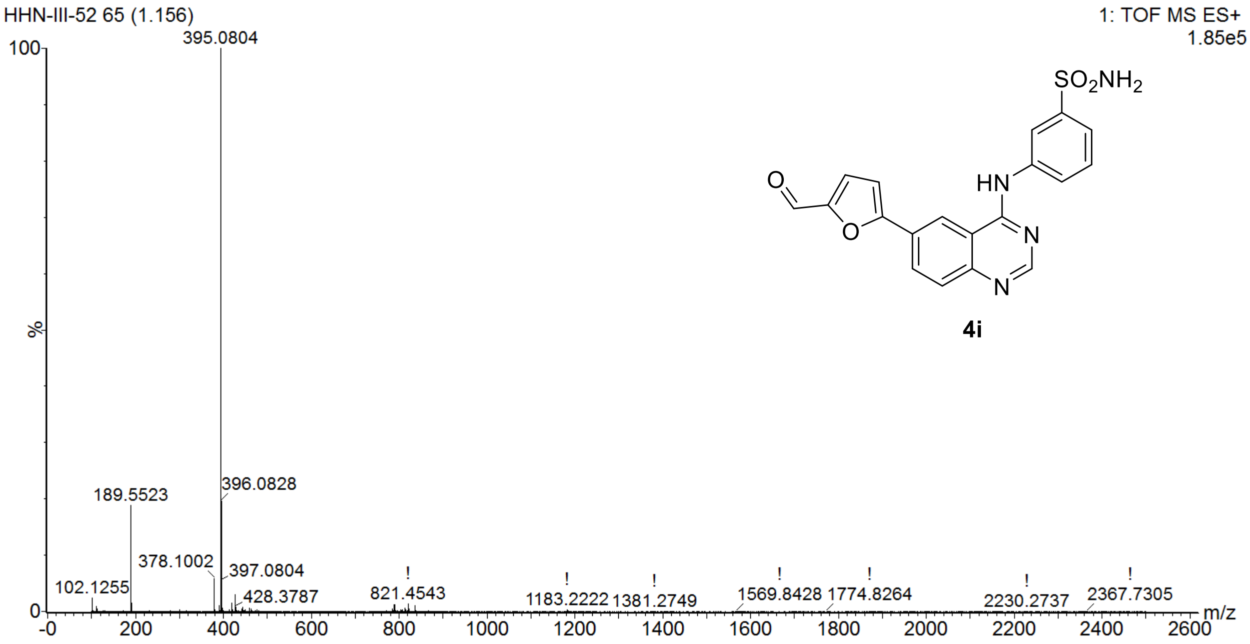
HHN-III-52 65 (1.156)
1: TOF MS ES+
1.85e5
395.0804
396.0828
189.5523
378.1002
397.0804
102.1255
428.3787
821.4543
1183.2222
1381.2749
1569.8428
1774.8264
2230.2737
2367.7305
%
m/z
SO2NH2
HN
O
O
N
N
4i

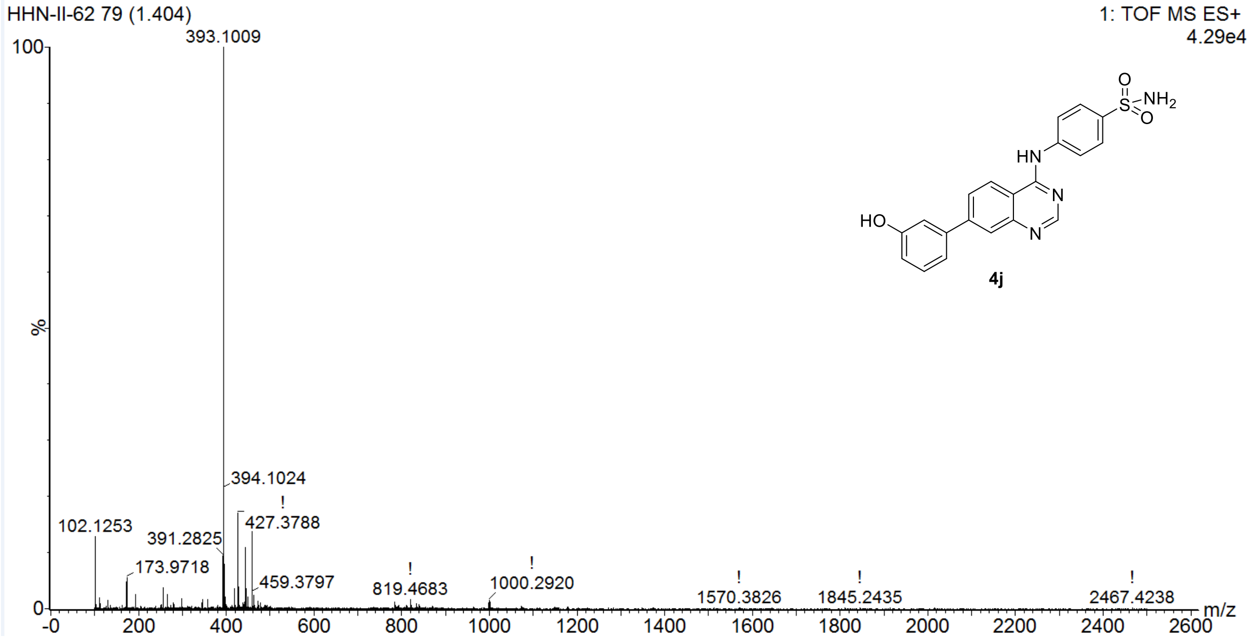
HHN-II-62 79 (1.404)
1: TOF MS ES+
4.29e4
393.1009
394.1024
427.3788
102.1253
391.2825
173.9718
459.3797
819.4683
1000.2920
1570.3826
1845.2435
2467.4238
%
m/z
O
S
NH2
O
HN
N
HO
N
4j

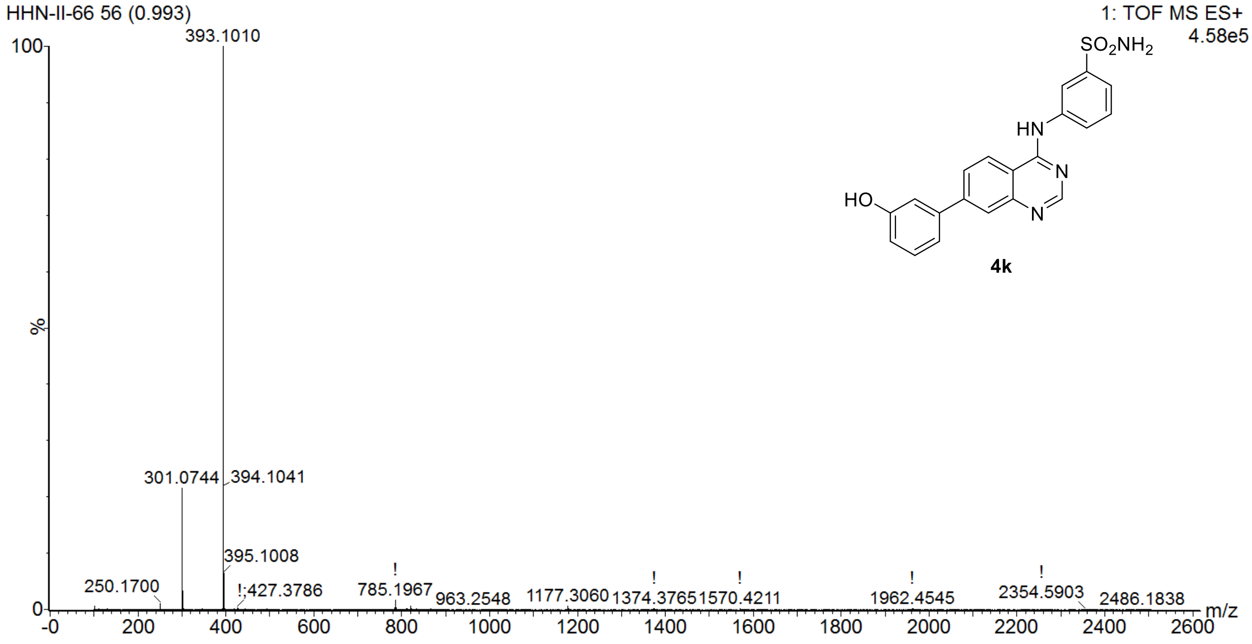
HHN-II-66 56 (0.993)
1: TOF MS ES+
4.58e5
393.1010
301.0744
394.1041
395.1008
250.1700
427.3786
785.1967
963.2548
1177.3060
1374.3765
1570.4211
1962.4545
2354.5903
2486.1838
100
%
0
m/z
-0
200
400
600
800
1000
1200
1400
1600
1800
2000
2200
2400
2600
SO2NH2
HN
N
N
HO
4k

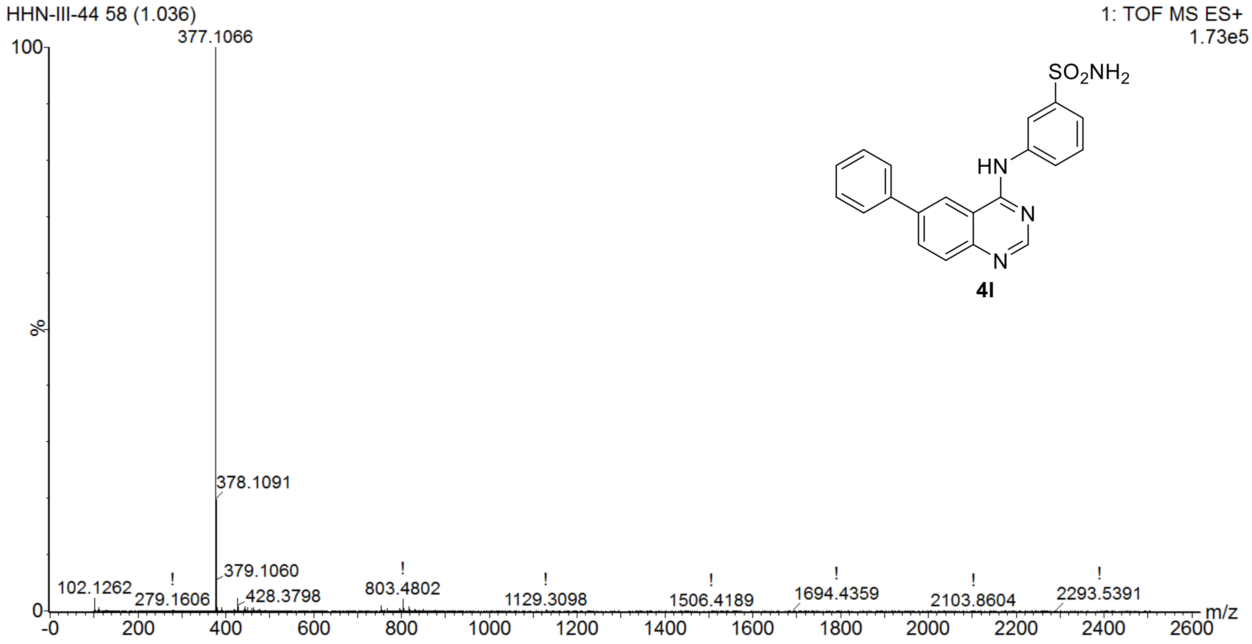
HHN-III-44 58 (1.036)
1: TOF MS ES+
1.73e5
377.1066
378.1091
379.1060
102.1262
279.1606
428.3798
803.4802
1129.3098
1506.4189
1694.4359
2103.8604
2293.5391
100
%
0
m/z
-0
200
400
600
800
1000
1200
1400
1600
1800
2000
2200
2400
2600
SO2NH2
HN
N
N
4l

4. Supplementary docking figures

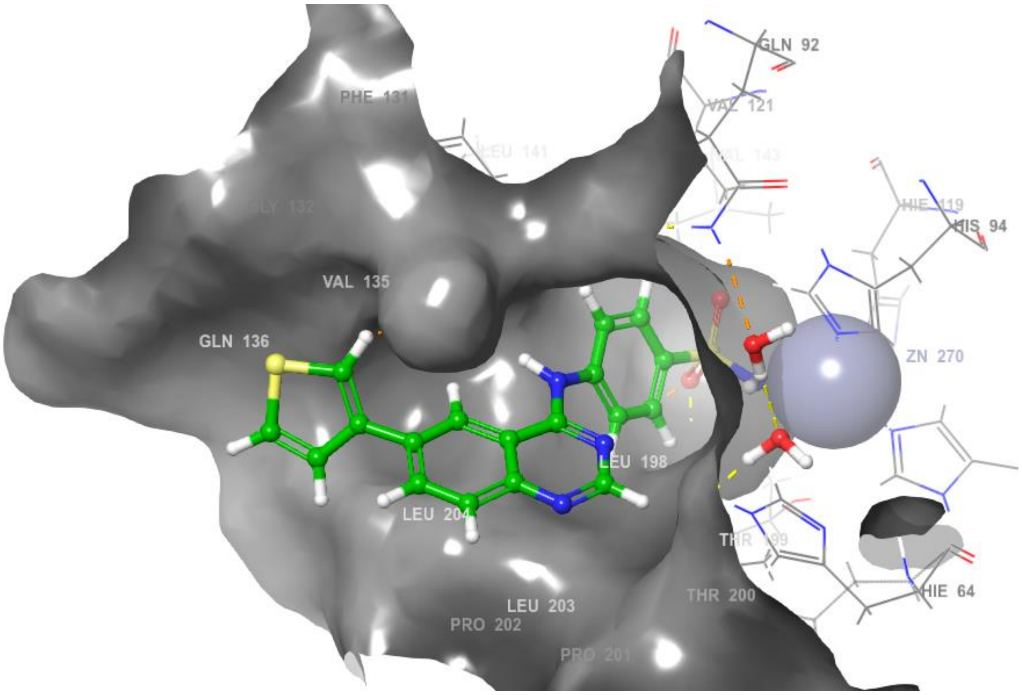

Figure S4.1. 3D model of the crystal structure of hCA II with compound **4f**.

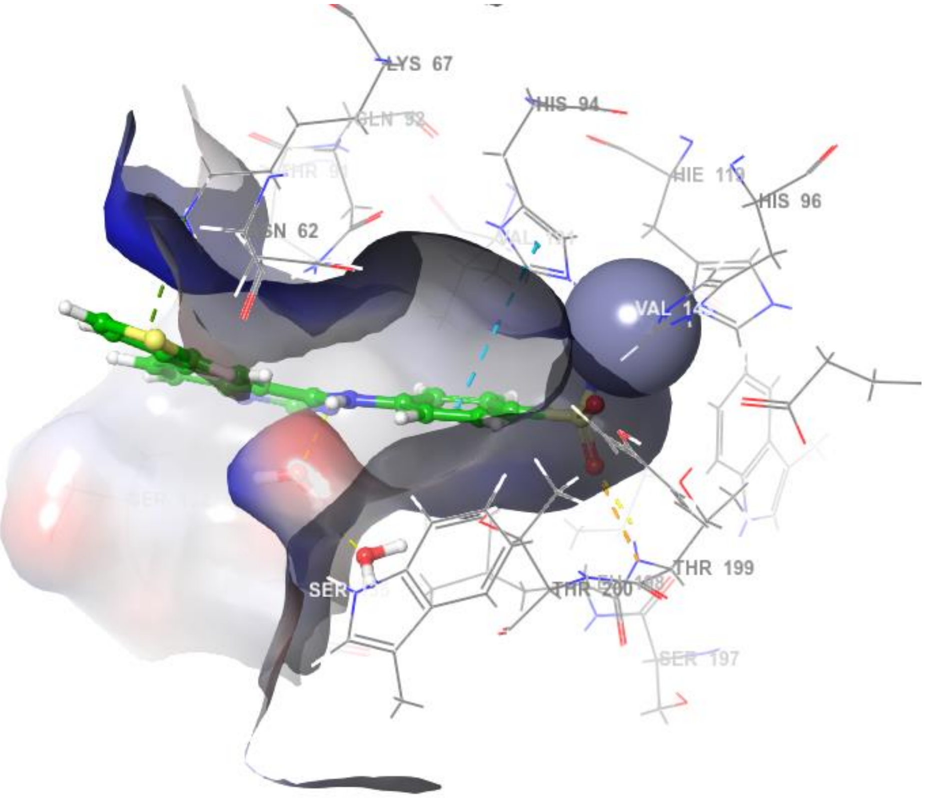

Figure S4.2. 3D model of the crystal structure of hCA XII with compound **4f**.

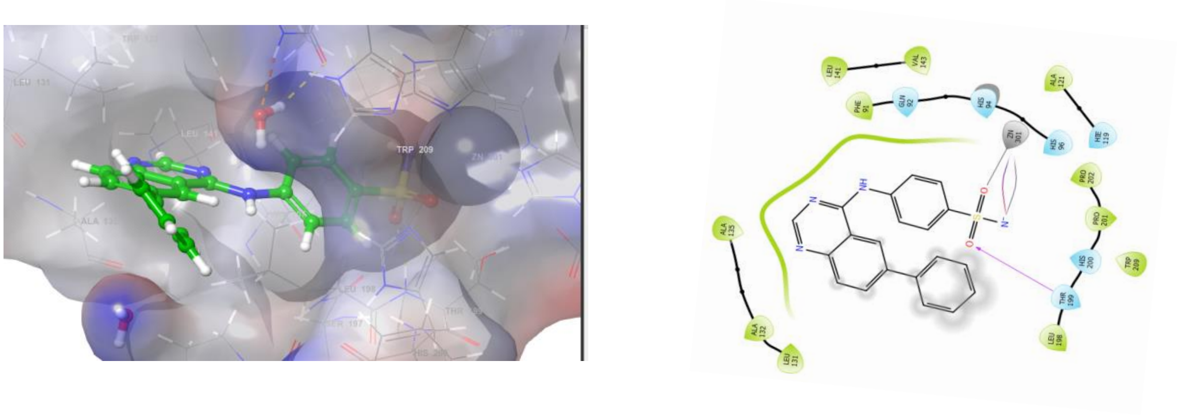

A B

Figure S4.3. The interaction patterns of compound **4a** in the active sites of hCAI (A) 3D model of the crystal structure of hCA I with compound **4a**. (B) 2D interaction pattern of compound **4a** with hCA I active site.

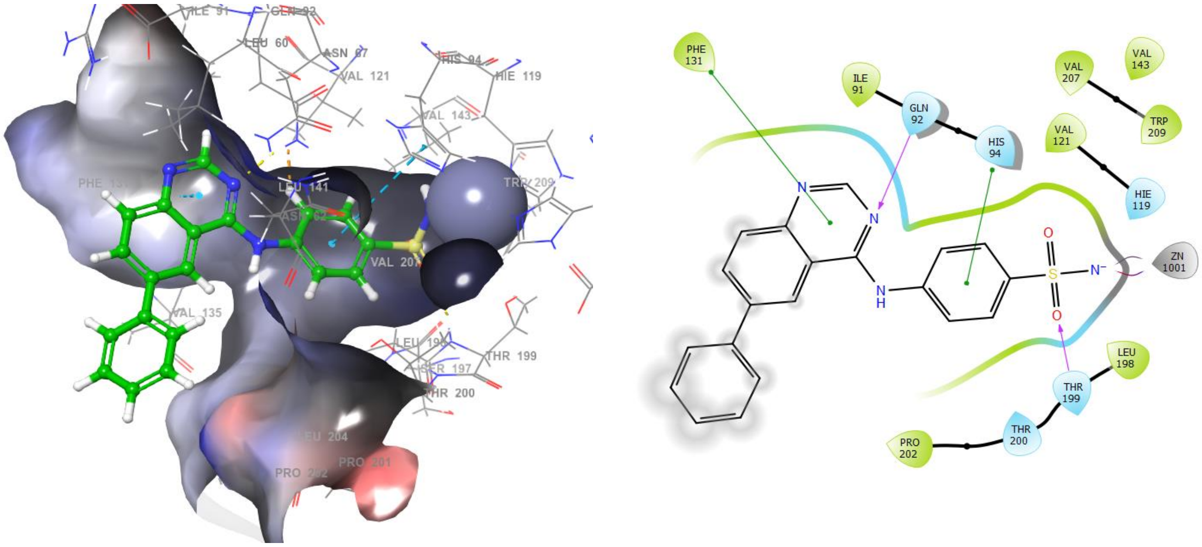

A B

Figure S4.4. The interaction patterns of compound **4a** in the active sites of hCA IX (A) 3D model of the crystal structure of hCA IX with compound **4a**. (B) 2D interaction pattern of compound **4a** with hCA IX active site.

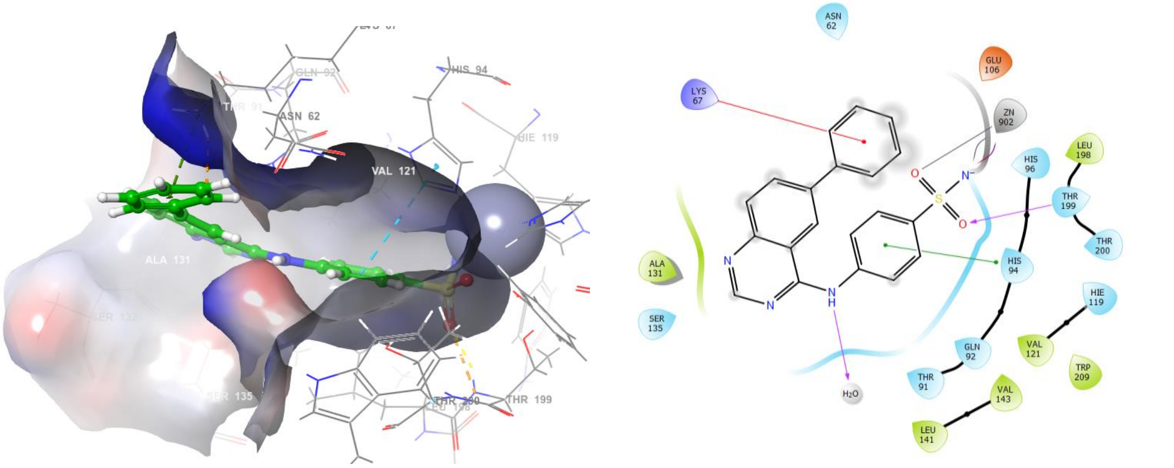

A B

Figure S4.5. The interaction patterns of compound **4a** in the active sites of hCA XII (A) 3D model of the crystal structure of hCA XII with compound **4a**. (B) 2D interaction pattern of compound **4a** with hCA XII active site.
